# Comprehensive review on endonasal endoscopic sinus surgery

**DOI:** 10.3205/cto000123

**Published:** 2015-12-22

**Authors:** Rainer K. Weber, Werner Hosemann

**Affiliations:** 1 Division of Paranasal Sinus and Skull Base Surgery, Traumatology, Department of Otorhinolaryngology, Municipal Hospital of Karlsruhe, Germany; 2 I-Sinus International Sinus Institute, Karlsruhe, Germany; 3 Department of Otorhinolaryngology, Head and Neck Surgery, University of Greifswald, Germany

**Keywords:** endoscopic sinus surgery, FESS, postoperative care after sinus surgery, nasal packing, outcome after sinus surgery

## Abstract

Endonasal endoscopic sinus surgery is the standard procedure for surgery of most paranasal sinus diseases. Appropriate frame conditions provided, the respective procedures are safe and successful.

These prerequisites encompass appropriate technical equipment, anatomical oriented surgical technique, proper patient selection, and individually adapted extent of surgery. The range of endonasal sinus operations has dramatically increased during the last 20 years and reaches from partial uncinectomy to pansinus surgery with extended surgery of the frontal (Draf type III), maxillary (grade 3–4, medial maxillectomy, prelacrimal approach) and sphenoid sinus.

In addition there are operations outside and beyond the paranasal sinuses. The development of surgical technique is still constantly evolving. This article gives a comprehensive review on the most recent state of the art in endoscopic sinus surgery according to the literature with the following aspects: principles and fundamentals, surgical techniques, indications, outcome, postoperative care, nasal packing and stents, technical equipment.

## 1 Principles and basics of endonasal sinus surgery

The present paper follows the traditions of the manuscripts written by Wolfgang Draf in 1982 [1] and Werner Hosemann in 1996 [2]. It will describe the current state of sinus surgery in consideration of the new developments that have taken place since 1996. It will show which evidence exists today (status July/August 2014) and which concepts and techniques are useful and helpful.

The paper is based on an extensive analysis of the literature, however, at the same time it is limited because the extreme and constantly growing number of literature as well as the limited time at disposition make it impossible to give a complete overview of the subject.

The assessment of new techniques and products must always bear in mind that economic considerations and marketing aspects might influence scientific publications. Also “premium” investigations with level I evidence must generally be questioned with regard to possible bias. Each footnote regarding the “Conflict of interest” must be carefully observed and well-known phenomena of reciprocity (reciprocity bias) must be considered.

The principle or objective of endonasal sinus surgery consist of the following aspects which may be achieved individually or in combination [3]:

1. Restoration or improvement of disturbed ventilation or drainage,
2. Removal of relevant foci of a disease (e.g. polyps, presumably irreversibly pathological hyperplastic mucosal foci, so-called osteitic bone trabeculae, accumulations of mucus, secretory concrements, tumors),
3. Preservation of the normal or only slightly altered mucosa,
4. The most possible protection of anatomical landmarks,
5. Realization of an approach to surgical therapy of a disease located beyond the paranasal sinuses, mostly a tumor (see complementary review written by Hosemann and Schroeder [4]) or a result of trauma (see complementary review written by Kühnel and Reichert [5]).

The indication to perform surgery of the paranasal sinuses is made in a synopsis of anamnesis with current complaints, combined with the findings of rhinoscopy and endoscopy as well as an adequate imaging (CT scan, CBT, if needed also MRI) [6]. Based on the individual extent of the disease, anatomy and other patient-specific factors an individual surgical strategy is developed. 

The requirements to perform surgeries in general and to indicate and perform endonasal sinus surgery in particular have significantly increased. Currently the following preconditions must be observed:

- Intensive medical consultation (counselling, informed consent), explanation of the surgical procedure, accompanying and postoperative treatment, complications and alternative options to surgery (Law of Patients’ Rights as of February 20, 2013, http://www.bmg.bund.de/glossarbegriffe/p-q/patientenrechtegesetz.html).
- Pharmacotherapy: see chapter entitled “Pre-treatment”.
- Sufficient surgical experience [7]. Surgical maneuvers exceeding limits of regular interventions should only be performed by surgeons who are specifically experienced in sinus surgery [8], [9].
- Sufficient equipment regarding instruments and technical devices to perform the planned intervention (see chapter on type of interventions and technical equipment). Any chief hospital manager has the obligation to equip the medical staff with adequate technical devices [10]. Preoperatively the question has to be answered if the objective of surgery, as it corresponds to the individual disease and anatomy and as it has been discussed with the patient, can be achieved with the resources at disposition.
- It is necessary to have at hand a current and appropriate CT scan (preferably ≥2 planes) for planning of the actual surgical project (see chapter on radiological diagnostics [3]).
- Profound knowledge of the specific endoscopic microanatomy. Endoscopic sinus surgery has induced numerous anatomical investigations on the anatomy of the paranasal sinuses and neighboring spaces. The exact knowledge of this field is essential for surgeons [11], [12] (see also complementary review on rhino-neuro-surgery [4]). The current nomenclature should be used in any operative report and discussions [12].
- When the indication for revision surgery is made, particular aspects must be taken into account: the current clinical impression of the patient should be focussed on, with the background information of original complaints; besides, the type, extent, and duration of the intercurrent conservative therapy as well as the actual findings of endoscopy and imaging (residual infection foci, micro-anatomical obstructions, or scars) [13], [14], [15], [16], [17], [18]. Most frequently, residual fronto-ethmoid cells are found, residues of the uncinate process, a lateralized middle turbinate, cicatricial stenoses in the frontal recess, a residual but obstructed natural maxillary ostium (so-called “missed ostium sequence”). It must be checked if the primary surgical objective has been achieved and if it is still relevant.

### 1.1 Pre-treatment

It is mandatory to indicate surgical interventions in non-emergency cases only after an adequate conservative (drug) treatment trial has proven to be ineffective [19]. This trial may be omitted if the patient explicitly does not agree to such a therapy – a fact which should be documented. The same holds true if the conservative trial seems definitively to be unpromising.

In cases of acute rhinosinusitis, conservative trials may include an intravenous antibiotic therapy with an appropriate antibiotic.

After the first infection episodes in cases of recurrent acute rhinosinusitis, the application of nasal steroids can be performed for prophylaxis, especially with simultaneous allergic rhinitis. However, the effectiveness is not proven. Reliable alternative medicamentous regimes for prophylaxis are not known.

In cases of chronic rhinosinusitis, often a so-called maximal pharmacotherapy is recommended and performed [19]. Up to know this regimen is not clearly defined based on evidence. The effectiveness of single drugs is critically discussed according to evidence-based criteria (see below). Apart from those limitations, a maximal medicamentous therapy of CRS currently consists of nasal steroids in higher doses, accompanying nasal rinsing with saline solution, antibiotic therapy for 2–3 weeks, and systemic steroids [19], [20], [21], [22], [23] [24]. Nasal steroids have a low potential of side effects and at least temporarily they are effective against CRSwNP [25], [26], however, less effective in CRSsNP [27], [28]. The direct application in the paranasal sinuses is more effective than the mere nasal application [28]. Nasal rinsing with saline solution is effective as accompanying therapy in all types of CRS, allergic rhinitis, acute rhinosinusitis, and for prophylaxis of frequent upper airway infections [29], [30], [31]. Irrespective to the high acceptance of antibiotic therapy in CRS as part of the recommended maximum [19], [32], the evidence of the respective treatment effectiveness is limited. In CRSwNP the application of doxycycline over 3 weeks leads to a little reduction of the polyposis; after 3 months, however, the symptoms were the same as at the beginning [33]. Patients with CRSsNP and low IgE who received roxithromycin observed a minimal reduction of their symptoms [34]. Antibiotic therapy is currently considered as an option [35]. It should be applied in all types of CRS revealing obvious purulent secretion – the choice of the specific drug, however, should be made after taking endoscopically guided swabs [6]. Macrolides seem to be effective due to their anti-inflammatory properties which is true especially for the subgroup of patients with low IgE. However, the actual range of effectiveness is limited and is often considered as clinically not relevant [36]. The long-term effectiveness of systemic steroids in CRSsNP as single therapeutic modality has not been evaluated or adequately proven up to now. Systemic steroids have always been part of a multimodal concept together with antibiotics and topical steroids. In most cases 10–60 mg are applied for 10–12 days. This is why they are only regarded as an option [19], [37], [38], [39] are recommended in individual cases only [6], [19], [37], [40], [41], [42]. Systemic steroids in CRSwNP are effective, but the duration of this effect is very limited [33], [42], [43]. Regularily, the short-term application is recommended [44]. In AFS, systemic steroids are effective but the duration of the treatment must inevitably be prolonged and thus often side effects must be expected [42]. The relevant incidence of different side effects of systemic steroids must be weighed up against their temporary effectiveness so that dose minimization is always aimed at and a specific informed consent should be taken prior to starting the therapy [42], [45], [46]. There is no consensus about an optimal cortisone dosage and duration of therapy. The applied dose is frequently determined by the packaging of the tablets at disposition. The following pharmaceuticals have been applied: e.g. prednisolone 50 mg for 14 days, methyl-prednisolone 32-16-8 mg for 1 week each, prednisolone 25-12.5 mg for 1 week each and 12.5 mg every two days in the third week [44]. In consideration of quality of life, risks, and costs, the actually first and only break-even analysis performed a calculation that CRSwNP requiring systemic application of cortisone every 2 years, CRSsNP and asthma every 12 months, and CRSwNP, asthma, and analgesic intolerance every 6 months do represent a borderline and beyond this limit surgery should be preferred [45]. Antileukotriene agents are also effective in cases of CRSwNP [47], [48]. An additional effect is possible with simultaneous application of nasal steroids in cases of symptoms of headaches and facial pains, itching and sneezing, postnasal secretion, and smelling disorders [47]. T or B cell defects should be excluded and treated if needed [49].

### 1.2 Radiological diagnostic measures

For careful indication and performance of sinus surgery it is obligatory that tomography is present. Only tomographic imaging allows the depiction and analysis of details that are relevant for surgery regarding anatomy, type and extent of the disease – due to this facts, imaging is indispensible [50], [51], [52].

The standard procedure for tomographic diagnostics of the paranasal sinuses is computed tomography (CT). The actual examination technique should be performed according to the recommendations of the working committee on head and neck diagnostics of the German Radiological Society [53] (http://www.drg.de/). Alternatively, more and more often the so-called cone beam tomography (CBT) is applied that ensures an excellent bony resolution in three planes with mostly lower radiation exposure – however, depiction of soft tissues is limited and the region of examination has to be somehow restricted [54], [55], [56], [57], [58], [59], [60]. Especially in CBT but also in CT scan attention must be paid that the display detail includes all clinically relevant anatomical areas.

Magnetic resonance imaging is recommended especially in cases of intracranial or progressive orbital complications of rhinosinusitis or also of malignant and special types of benign tumors [52], [53], [61], [62]. Regardless of a poor resolution of the bony structures, it is considered as reasonable to use magnetic resonance imaging in routine cases with limited extent of the disease or in children (radiation exposure) for diagnostics and also for surgical therapy. A problem of MRI that must be taken into consideration is that alterations of mucosal swellings and blood circulation in the context of the nasal cycle might be confused with inflammatory findings of acute or chronic rhinosinusitis.

The indication for CT/CBT and CT/CBT control examinations must be made very restrictively (ALARA principle: as low as reasonably achievable), frequent follow-up examinations should be avoided [63]. The radiation dose in modern imaging (CT/CBT) is low, however, the range of variation is apparently large, also according to data from different countries [64], dosage varies between 0.1–2 mSv [23], [57], [64]. Literature reveals a vast number of specific efforts to reduce radiation which are hard to be evaluated by non-radiologists. In comparison, the natural radiation exposure amounts to 3 mSv per year, the one of flying personnel is 5 mSv [23], [65], [66], On the other hand, the induction of tumors by radiation exposure is regarded as generally proven [67], [68], [69], [70], [71]. There are different estimation to which extent radiation exposure of CT diagnostics might induce tumor growth [68], [72], [73], [74]. Also the development of cataract increases with higher radiation exposure of CT diagnostics of the head and neck region, depending on the dosage [75].

CT/CBT is indicated when a relevant therapeutic decision has to be made. The more extended the disease is and the more difficult the anatomical situation presents, the more precise the respective CT scan should be, requiring imaging in 3 planes at the end. The decision if in a given case a CT scan of one plane before the intervention is sufficient must be made individually and depending on the complexity of the disease and the intervention. A systematic evaluation of the imaging is done in any patient to assess the extent of the disease as well as the individual anatomy and special anatomical variations that might be relevant for surgery. Table 1 (Tab. 1) gives an overview of existing evaluation systems and CT check lists [50], [51], [52], [56], [76], [77], [78], [79], [80], [81], [82], [83], [84]. Recommendations of literature try to restrict analysis of imaging to 5 defined general analytical steps [83].

An interpretation of the scans must generally take into consideration that CT scans reveal in up to 40% and MRI in >60% of the population irrelevant focal swellings of the mucosa and that any intercurrent acute infection needs several weeks to disappear radiologically [85], [86], [87], [88], [89], [90]. Those factors must be borne in mind, not only during evaluation but also when fixing an appointment for radiological examination.

### 1.3 Surgical checklists

The application of surgical checklists (e.g. WHO check list, [91]) is an established and recommended tool as part of a sytematic process to reduce surgery-related incidences of complications and mortality [92], [93], [94]. However, their benefit is limited if the check lists are filled out incomplete and routinely just to fulfill a daily duty [95]. Concerning sinus surgery the following aspects should be observed carefully:

- The CT scans of the patient should be present in the operative theater, current and the side orientation should be correct.
- If a navigation system is planned to be used, this system should be duly prepared and appropriate CT scans should be present.
- The drugs to be applied should be labelled correctly.
- Electro-coagulation should work properly, if needed grounding for monopolar coagulation should be installed properly.
- Suction should work properly.
- Gauze and swabs should be armed and counted [96], [97].

### 1.4 Preparation of the surgery site

Preparatory and anesthesiologic measures all pursue the objective to reduce bleeding during surgery as much as possible in order to increase the precision of the intervention, to minimize risks, to better achieve the planned result of the surgery, to reduce the duration of the intervention, to minimize the postoperative wound healing processes and granulation reaction and scaring, and to have a minimal blood loss [98].

This aspect becomes even more important as in a prospective study applying multivariate analysis, the only independent risk factor for the necessity of revision was intraoperative bleeding [99].

Beside an atraumatic surgical technique the following measures are appropriate and helpful according to recent randomized studies:

- Topical vasoconstriction, e.g. my means of application of adrenalin via gauze or comparable swabs (concentration: 1:1,000; in children or risk patients: 1:2,000 [100], [101], [102], [103], [104], [105]). Topical application causes significantly lower peak serum levels than injection [102], [105] increasing security. Contraindications and safety measures must be observed [103], [106]. Regarding the application of imidazoline derivatives, it is mandatory to observe the allowed maximum quantities because especially children might develop toxic reactions (cardiovascular, central nervous disorders) [107], [108]. Cocaine and adrenaline are similarly effective [109]. Injections with 0.25% bupivacaine with adrenaline 1:200,000 neither reduce intraoperative blood loss nor the duration of surgery but lead increased mean arterial blood pressure [110].
- Appropriate choice of anesthesia. Today TIVA (total intravenous anesthesia with propofol and remifentanil) is the preferred type and at least a small advantage can be seen in comparison to inhalation or balanced anesthesia [104], [111], also in children: [112]. However, the discussion is still open which individual drugs might cause the positive effect and which combination of drugs should be preferred [113], [114]. Reduction of the cardial “output” seems to be main target [96]. Specific additional drugs commonly used are beta blockers like esmolol [112], [115] or clonidine [116], [117], [118].
- Use of a laryngeal mask instead of an intratracheal tube [119], [120], [121], [122]. The protection of the airways in children was not worse than with intubation [123].
- Reduction of the ventilation pressure (positive endexpiratory pressure) [124].
- Reverse Trendelenburg position of the patient/operating table [125], [126], [127]. An inclination of 20–30° is recommended as being effective and safe [128], [129], [130]. Practical advice: the angle can be measured by means of a smartphone app.
- Infiltration of the fossa pterygopalatina with local anesthetic and adrenaline [131], which, however, was not confirmed in another study [132].
- Pre-treatment with topical steroids [133].
- Pre-treatment with systemic steroids [13], [83], [134], [135], [136], [137], [138]. The vast majority of US American ENT specialists applies preoperatively systemic steroids in cases of CRSwNP, although there is no clear evidence for effectiveness. Usually 30–50 mg (up to 1 mg/kg) prednisolone are given for 5–7 days [139]. The arguments of reduced inflammation, better intraoperative overview, and reduced intraoperative bleeding must be weighed out against the possible concealing of the true extent of the disease by artificially improvement with subsequent only short-term effective surgical therapy and possible side effects of systemic steroids, even in cases of short-term application, not to mention accumulations over lifetime [46]. Up to now, there is no evidence for the one or the other assumption. The following conclusion may be drawn: preoperatively applied systemic cortisone in appropriate doses can improve the conditions for surgery of CRSwNP, however, it cannot be considered as a must.
- Interruption/change of drug application affecting coagulation[128], [140]. This aspect concerns also numerous phyto-pharmaceuticals (2 weeks preoperatively). Regarding the perioperative handling with anti-coagulants and antiplatelet drugs the current literature has to be considered [141] (see also corresponding comprehensive review). Generally the difference is made between an emergency intervention and elective surgery. A balance must be found between the risk of thrombosis and the bleeding risk. Whereas for example the risk of bleeding complication during a surgical intervention was increased by 1.5 with maintained ASS medication, the degree of severity of bleeding complications and the perioperative mortality were not increased (possible exceptions: intracranial surgery, prostatectomy) [142]. Thus, numerous sinus interventions may be performed based on a given indication irrespective to the application of ASS.
- The insertion of pharyngeal tamponades does not lead to reduced postoperative nausea and vomiting (PONV), but increases the postoperative pains in the oral and pharyngeal space [143], [144], [145], [146], [147], [148], [149]. Hence it can be omitted in routine cases.
- The application of tranexamic acid, systemic or topical, leads to reduced bleeding by 30–40% [150], [151], [152], [153], [154]. The quality of the surgery site improves [154]. It is not yet definitively clarified if thrombo-embolic events are more frequent or not after application of tranexamic acid. As systemic application, a dosage of 1 g per day is sufficient [151]. As topical application, 100 mg as spray solution at the end of surgery have been applied endonasally [155].
- Intraoperative rinsing with hot water (49°C, 20 ml every 10 minutes) only led to a visible improvement of the surgery site after a duration of >120 minutes. The objective blood loss, however, was reduced also for shorter durations (1.7 ± 1.1 ml/min vs. 2.3 ± 1.0 ml/min) [156].

### 1.5 Antibiotic prophylaxis

The currently existing evidence does not support the routinely performed perioperative antibiotic prophylaxis in routine interventions of the paranasal sinuses [157]. According to a current meta-analysis, postoperative infection rates, symptoms, or endoscopy scores were not significantly improved after application of antibiotics [158].

While bacteremia was found in 7% of the patients with chronic rhinosinusitis at the beginning of endoscopic sinus surgery, it could no longer be proven at the end of the intervention without meanwhile performed antibiosis [159]. This led to the conclusion that routine application of antibiotics was not necessary [159].

In the context of more extensive surgeries, interventions at the skull base, and risk factors of infections, a perioperative antibiosis is appropriate and must be discussed individually regarding its necessity [160], [161]. The recommendations of the expert panel of the Paul Ehrlich Society lists pre-, intra-, postoperative, and patient-specific factors that may lead to an increased infection risk [161]. An increased rate of *Pseudomonas aeruginosa* and other gram-negative germs was found in patients with diabetes mellitus in the context of endoscopic sinus surgery for chronic rhinosinusitis, but not *Staph. aureus*, which has to be considered regarding therapy of possible infections [162].

For endoscopic skull base surgery, the application of an antibiotic for 24–48 hours was sufficient, independently from intraoperative CSF leakage [160].

If antibiotics are part of the following treatment concept of the original, e.g. inflammatory, disease, the first dose should be applied like the mere perioperative prophylaxis immediately before starting surgery.

The vast majority of authors perform perioperative antibiosis in the context of duraplasty [163]. It occurs as intravenous dose as long as nasal packings or lumbar drainage are in situ and should be sufficiently effective against *Staph. aureus* [163], [164], [165], [166]. There is no proven evidence confirming the benefit of long-term antibiosis going beyond this period of time [165]. Reports about complication-free endonasal duraplasties with use of nasal packing without antibiotic therapy have been published [167].

A routinely performed antibiotic prophylaxis is not indicated or recommended in cases of fractures of the frontal skull base with rhinoliquorrhea/dural lesion. The majority of the studies as well as a current meta-analysis could not reveal an advantage regarding the reduction of intracranial infections or mortality. In contrast, the risk of a selection of resistant bacteria increases [168], [169].

However, there is a clear indication for surgery of duraplasty in the case of fracture of the frontal skull base with rhinoliquorrhea/dural lesion (see chapter on duraplasty).

In summary, it is also true for endonasal endoscopic sinus surgery that a routinely performed antibiotic therapy is not required but a critical weighing up of the benefits and risks with consideration of well-known influencing factors. Prophylactic antibiotic treatment that is indicated in individual cases is usually applied only for a short period of time.

## 2 Type and technique of endonasal sinus surgery

### 2.1 Optical instruments

Today, endoscopy is considered as standard in diagnostics and therapy of most diseases of the paranasal sinuses [19], [170], [171], [172], [173]. The multitude of available endoscopes and technical equipment allows a diagnostic and therapeutic approach to nearly all regions. A previously performed investigation on the spatial handling security, the endoscope was at least equal with the binocular surgical microscope [174].

However, a more recent study revealed that the surgical exactness of performing different tasks was higher in unexperienced neurosurgeons using a microscope in comparison to using an endoscope. More experienced surgeons had an equal failure rate. The velocity in beginners and experienced surgeons was higher when they used a microscope [175].

Due to important technical development, the endoscope compared to the microscope is superior as optical device. It combines a very good overview due to wide angle technology with a very good detailed view due to HD technology, even in bloody sites. It allows looking around the corner by using angular optics under ergonomically favorable conditions due to video endoscopy. Only by means of endoscopy, a four-hand technique is possible. Even for education, training, and the control of surgical steps the endoscopic technique has more advantages. Even supervision of surgery is possible by means of teleconferencing [176].

If older systems are used, video endoscopy provides poorer images than the direct view through the endoscope [177]; the time-loss in a nasal training model (touching different hidden spots) was increased [178].

The use of modern HD video endoscopy leads to a significantly better image quality in comparison to older systems. Based on this fact, medico-legal consequences must be considered. **It is a major obligation of a hospital to provide the instruments that correspond to actual international standards **[10]**!**

It must be mentioned that an unimpaired, “binocular” view with the headlight allows the comparably most rapid and secure acting so that it may still be considered as acceptable to control certain minor intranasal manoeuvers [3], [174], [179].

In current surgery manuals, the application of the surgical microscope is no longer mentioned, apart from one exception [180]. The surgical technique with simultaneous use of microscope and endoscope, as it had been promoted by Wolfgang Draf for several years, was left by the majority of the surgeons.

Generally, the use of a microscope further leads to a more severe traumatization in the area of the nasal entry and the turbinates. Thus the application of the microscope alone can no longer be recommended.

### 2.2 Concept of endonasal sinus surgery

The concept of functional endoscopic sinus surgery is based on the publications of Messerklinger [181], [182], [183], according to which disturbed mucociliary clearance and narrow areas of the ostiomeatal unit are described as origin of recurrent and chronic rhinosinusitis. The concept of conventional FESS that is known and established since many years aims at treating inflammatory diseases of the maxillary and frontal sinus and the anterior ethmoid by resecting anatomical and/or inflammatory disturbing factors in the ostiomeatal unit and at the same time preserving the marginal mucosa and avoiding an extensive radical intervention [181], [182], [184], [185], [186], [187], [188], [189], [190].

The so-called minimally invasive sinus surgery (MIST, minimally invasive sinus technique) is understood as the further development of FESS. Promoters of MIST consider it sufficient to enlarge the narrow clefts of ethmoid [191], [192], [193], [194], [195], [196], even in cases of more extended disease. An essential part of the MIST concept is the use of the shaver that should increase the surgical precision. The single steps encompass: uncinectomy with exposure of the natural maxillary ostium, removal of the postero-medial wall of the agger nasi cells, if needed also mini-trepanation of the frontal sinus with rinsing, opening of the bulla ethmoidalis, repositioning of the middle turbinate (medialization is not defined in detail), if needed opening of the posterior ethmoid, if needed removal of polyps before the sphenoid ostium, if needed dilatation of the access of the sphenoid sinus. This concepts seems to be inconsistent in so far, as optionally a significant extension of the surgical measures is offered, the shaver as integral part has not proven to lead to superior results, and in contrast to the alternative contemporary concept of avoiding nasal packing the local insertion of nasopore, gel film, or merogel is performed. So there is no evidence for the superiority of MIST in comparison to other surgical concepts.

Today, FESS is the gold standard of surgical therapy of chronic rhinosinusitis [19], [173], [197], [198]. The extent of appropriate surgery, however, is still variable in actual concepts of FESS – the respective differences are not highlighted by specific evidence [197]. 

Since Messerklinger’s first descriptions, the knowledge of the detailed anatomy of the paranasal sinuses as well as the pathophysiology and therapy of CRS has significantly improved and enlarged [199], [200]. Apparently, CRS is caused by multiple factors and includes many subtypes, which is extensively described by Bachert in his complementary review to this present paper [201].

Generally, variations of microanatomy are not considered as the main cause of diffuse CRS [19], however, in single cases they may be meaningful, for example in cases of circumscribed forms of chronic rhinosinusitis [19], [202]. In recurrent acute rhinosinusitis, anatomical variations (such as a narrow infundibulum ethmoidale, spacious infraorbital cells) play a disease-promoting role [203].

Disturbed ventilation and drainage of the paranasal sinuses due to obstruction of the ostiomeatal unit is certainly important in part of the patients with CRS while others have a diffuse inflammatory process that is predominant and/or other factors contribute to persisting inflammation [19]. Also the importance of the mucociliary clearance regarding the results after endonasal sinus surgery is not definitively clarified [204].

Whereas current investigations found a significant correlation between obstruction of the ostiomeatal unit and a disease of the maxillary, anterior ethmoid, and frontal sinus in CRSsNP patients or a non-eosinophilic CRS, this could not be revealed for eosinophilic chronic rhinosinusitis or CRSwNP [204], [205], [206]. The creation of a large maxillary window did not influence the stenosis of the maxillary ostium caused by recurrent polyps [207]. 

Current concepts emphasize that

- pro-inflammatory cells and tissue parts (“inflammatory load”, polyposis with basally located T cells, biofilm, mucus retention with pro-inflammatory cytokines, altered bony areas) should be completely removed in order to achieve a better therapeutic result [204];
- the surgical interventions in advanced CRS should create optimal preconditions to allow local anti-inflammatory therapy;
- in cases of irreversible mucosal disease and proven disturbed mucociliary clearance a radical surgical procedure with removal of the irreversibly diseased mucosa as well as the creation of large drainage openings is necessary [208].

This leads to a surgical concept that includes the creation of larger openings (maxillary sinus: maximal middle meatal antrostomy, if needed variations of medial maxillectomy, canine fossa trephine approach; frontal sinus: type III) in cases of advanced disease (high CT score according to Lund-Mackay or Kennedy despite maximal medical therapy; eosinophilic CRS; bronchial asthma; analgesics intolerance; recurrence disease) and thus finally a unique cavity without relevant separations that can be accessed for local anti-inflammatory therapy [200], [204], [205], [208], [209].

A complete removal of the mucosa (“stripping”) should generally be avoided and the basal membrane should be preserved because it leads to fibrosis and osteoneogenesis [207], [209], [210], [211], [212]. On the other hand, polyps should be removed consequently down to the basal membrane [204] because the eosinophils are located at the base of the polyps [213] and residual polyps contain CD8-positive memory cells [214], [215], [216].

Especially for therapy of advanced diseases, usually surgery and drug therapy have to be combined whereby the topical therapy plays a crucial role because of effectiveness- and safety reasons [199]. A topical therapy of the paranasal sinuses is only sufficiently possible if open accesses to the paranasal sinuses are present which presupposes surgery [200], [217]. The topical therapy succeeds the better, the more those accesses are opened [218]. The maxillary ostium should have a width of at least 4–5 mm [217]. The frontal sinus can be best treated topically by application of type III drainage [218]. Nasal rinsing is better able to reach the paranasal sinuses compared to sprays, drops, or inhalations [209], [217].

The promoters of an extensive and radical surgical technique invoke a series of studies that report on very good results either in comparison with conservative surgery or in cases of therapy refractory rhinosinusitis after failed previous surgery – however, the majority of the respective literature reports are based on retrospective case series only:

- Better results in comparison with conservative sinus surgery in general:
  - [219], [220]: recurrent polyposis after 5 years 22.7% vs. 58.3%,
  - [221]: revision rate after 3 years 4% vs. 12.3%,
  - [222]: revision after up to 11 years 0/9 vs. 6/16, [223]: symptoms eliminated in >80% after >36 months.
- Treatment results in cases of therapy refractory maxillary sinusitis after previous surgery by means of medial maxillectomy (with partial resection of the inferior turbinate), Caldwell-Luc surgery, endonasal Denker’s surgery or canine fossa trephine (CFT):
  - [224]: 92% were successful after 6–61 months,
  - [225], [226]: prospective, improvement of the quality of life and of nasal obstruction and rhinorrhea after 2 years in 60–74%,
  - [227]: significant improvement of the symptoms after 2–5 years in 84%,
  - [228]: prospective comparison of CFT vs. only maxillary fenestration of the middle meatus, recurrence rate of 2/28 vs. 6/26, revision rate of 1/28 vs. 4/26,
  - [229]: retrospective case control study: symptoms, MRI, and endoscopic findings were better in CFT than in single maxillary fenestration of the middle meatus,
  - [230]: only 1/19 with postoperative persisting inflammation after 19.5 months,
  - [231]: 6/9 completely and 3/9 partially free of complaints after 4–86 months,
  - [232]: 72% were free of complaints, additional 8% after 35 months with additional drug therapy,
  - [233]: 74% were completely and 26% partially free of complaints after 11 months.
- Even one prospective randomized study could prove the superiority of more radical procedure in CRSwNP: symptoms, postoperative consumption of drugs were significantly lower, the degree of swelling in CT scans and the endoscopy score were lower than in patients who underwent surgery also via the inferior meatus in addition to maxillary sinus surgery [234].
- Therapeutic results in therapy-refractory rhinosinusitis with special consideration of the frontal sinus: comparing frontal sinus drainage type III to frontal sinus drainage type IIa, the revision rate after 12 months was 7 vs. 37% [24], so that a primary frontal sinus drainage type III can be taken into consideration if risk factors such as advanced CRSwNP, asthma, and a small frontal sinus access are diagnosed [8]. When frontal sinus surgery was performed in cases of clinically evident involvement of the frontal sinus in CRS, the revision rate amounted to 14.1% vs. 19 % after 5 years [235], [236].
- Better therapeutic results were obtained after pro-active partial resection of the middle turbinate [237], [238], [239].

Also in more extensive interventions, a general and maximized resection of the turbinates should be avoided in order to prevent subsequently side-effects like permanently eliminated mucosal function (see http://www.emptynosesyndrome.org/). 

On the other hand, radical endonasal surgery according to Denker does not seem to lead to empty nose syndrome or ozaena [225], [227]. Neither has empty nose syndrome been described for frontal sinus drainage type III [240], [241], [242], [243]. In a meta-analysis of 612 patients, relevant crust formation was found in <2% of the cases [244].

### 2.3 Classification of sinus surgery

There is no universal classification of sinus operations even if it was desirable, already with regard of quality management [245]. Different classifications have been elaborated, among them especially the classification of frontal sinus drainages (according to Draf) has been widely accepted. Details of such a classification would be: uncinectomy (infundibulotomy), maxillary sinus fenestration/maxillary sinus surgery, anterior ethmoidectomy, posterior ethmoidectomy, sphenoid sinus fenestration/sphenoid sinus surgery, frontal sinus drainage/frontal sinus surgery type I-III, and pansinus surgery [83], [240], [246], [247], [248].

Only an intervention that includes middle meatal antrostomy, anterior and posterior ethmoidectomy, opening of the sphenoid sinus, and frontal sinus drainage type IIa should be called “pansinus operation”.

### 2.4 Staging of chronic rhinosinusitis

To describe the extent of CRS, there are different classification systems. The following three are the most widespread, and in combination they allow at the same time an exhaustive description of the disease.

E+ and E- can be added in order to describe an increased or reduced tissue eosinophilia, respectively.

#### 2.4.1 Staging of chronic rhinosinusitis according to Kennedy based on the CT findings 

(see Table 2 (Tab. 2), [249])

#### 2.4.2 CT score in chronic rhinosinusitis according to Lund and Mackay 

(see [250], [251])

For each of the paranasal sinuses (maxillary sinus, anterior ethmoid, posterior ethmoid, frontal sinus, and sphenoid sinus) scores (0–2) are given for each side separately:

0 = no opacification

1 = partial opacification

2 = total opacification

Additionally scores (0 or 2) are given for the ostiomeatal unit of each side:

0 = no obstruction

2 = obstruction

Hence, these score may achieve values between 0 and 24. An average value in healthy people amounts to about 4.26 [88].

#### 2.4.3 Classification of nasal polyposis according to Malm based on nasal endoscopy 

(see [252])

Malm 0 = no nasal polyposis

Malm 1 = nasal polyps in the middle meatus not reaching the lower edge of the middle turbinate

Malm 2 = nasal polyps reaching deeper than the middle turbinate but do not touch the nasal floor

Malm 3 = nasal polyps reaching the nasal floor.

### 2.5 Technique of endonasal endoscopic sinus surgery

Regarding the technique of endonasal endoscopic sinus surgery, there are a series of current and well established monographs that will be mentioned in this section [83], [170], [12], [180], [247], [253], [254], [255], [256], [257], [258], [259], [260], [261], [262], [263], [264], [265], [266], [267], [268] [269], [270], [271], [272], [273], [274].

Indications for transoral/transfacial surgery of the paranasal sinuses has become very rare [275], [276]. They are not the topic of this review.

Usually, a patient is focused on his disease and is primarily interested in the possibly curative treatment, followed by aspects of function and post-therapeutic morbidity as well as finally aesthetic reflections. Any patient will sum up all the aspects mentioned when he chooses therapy and also the surgical approach following intensive counselling.

The extent of the intervention is individually adapted according to

- Type and extent of the disease,
- Type and extent of the complaints,
- The individual macro- and micro-anatomy
- As well as patient-specific factors.

Up to now it could not be satisfactorily clarified if and how it may be possible to find out before surgery which patient should undergo which type of surgery with the best cost-benefit ratio. It is hard to predict, for which patient a small intervention is sufficient, and when extensive surgery is justified and necessary. According to the general opinion, minor disease requires only circumscribed surgery. The extent of the intervention increases with the extent of the disease, especially in CRS. The “extent” hereby is unclear and controversially discussed.

#### 2.5.1 Uncinectomy

Apart from variations of the access to treat isolated diseases of the sphenoid sinus, nearly every sinus surgery starts with uncinectomy, at least in patients who had not undergone previous interventions. Only uncinectomy allows the precise identification of the natural maxillary ostium and the exposure of the infundibulum ethmoidale as natural drainage pathway of the anterior ethmoid and the frontal sinus.

If needed, surgical measures at the nasal septum and the middle turbinate may precede, in rare cases also at the inferior turbinate in order to achieve sufficient space to access the middle meatus.

Uncinectomy may be performed in anterior-posterior direction or retrograde from posterior to anterior. Using the anterior-posterior technique, the uncinate process is incised near the attachment at the lateral wall. The incision is extended in superior and inferior direction, expanding the infundibulum ethmoidale, which is located behind it, by medial movement of the instrument at the same time. After removal of the mobilized part of the uncinate process, the remaining horizontal part can be taken from its mucosal pouch and resected and the surplus mucosa is removed. Thus, the natural maxillary ostium is completely exposed and can be examined with regard to its size and possible mucosal swellings. Endoscopy of the maxillary sinus is partially possible. Up to this point, the mucosa of the maxillary ostium is still intact.

Regarding the posterior-anterior technique, the uncinate process is incised starting at the free edge from dorsal in anterior direction or punched out and from there the horizontal part is detached and the surplus mucosa is removed.

The swing door technique implies the additional incision and removal of the middle part of the uncinate process already at the beginning [277].

Complete uncinectomy with removal of the cranial part usually opens the view to the agger nasi cell.

A typical surgical risk with subsequent failure of the surgery is missing the natural ostium because of leaving a too big part of the uncinate process behind [16], [17] (occurring in 42 of 636 cases in anterior-posterior technique, [277]). A visible accessory ostium (prevalence around 10%) may mislead the surgeon taking this ostium as the primary one [16], [17], [278]. The anterior-posterior technique bears the specific additional risk of penetrating the lamina papyracea which is much smaller in the posterior-anterior technique [274], [277]. This is especially true for cases, where the uncinate process stands laterally or is retracted as for example in the case of a “silent sinus syndrome”.

#### 2.5.2 Maxillary sinus surgery

The basic principles of maxillary fenestration and middle meatal antrostomy were formulated many years ago and have not changed since then [211], [279].

First objective of maxillary sinus surgery is the precise identification and assessment of the natural ostium. This requires the use of optics with an angulated view [211]. Depending on the individual anatomy and type and extent of the disease, adapted extension is performed.

The optimal size for the middle meatal antrostomy is unclear [211], [279], [280]. There are recommendations to preserve sufficiently sized natural ostia in certain cases [211], [279], [281] (e.g. in cases of recurrent acute maxillary sinusitis, dental maxillary sinusitis), and to enlarge other ostia in more severe disease or in need of more intensive surgical measure are or have to be performed in the maxillary sinus itself. A rough classification differentiates between preservation (grade 1), moderate, and extended (maximal) enlargement (grade 2 and 3, respectively; [83], [274]). A moderate enlargement for example is recommended when surgical measures in the maxillary sinus are necessary, like suction of secretion or removal of mucosal structures [83], [274]. A tendency of about 50% stenosis must be calculated [281], [282]. In cases of severe disease (CRSwNP, recurrences, eosinophilic rhinosinusitis, allergic fungal sinusitis) usually a maximal enlargement of the maxillary sinus via the middle meatus is recommended [1], [209], [263], [265], [274], also in order to create favorable conditions for postoperative rinsing whereby the maxillary opening should be at least 4–5 mm [217].

The permanent opening of the maxillary sinus is bigger if the natural ostium was additionally enlarged intraoperatively after uncinectomy [281], [283]. Furthermore, better eradication of eosinophils in the mucosa was observed [284] as well as a lower Lund-Mackay score in the CT scan [283] after enlargement of the ostium. However, the patients’ complaints were equal regardless of the size of the opening [281], [283].

An accessory ostium should always be connected to the natural ostium in order to avoid recirculation [279], [285]. Infraorbital cells might narrow the natural ostium and should be removed [211], [279].

The statements that a big opening would favor the development of biofilms and cause desiccation [286] have not been proven. Regarding maxillary sinuses that bulge out in medial direction, however, it is recommended to create only smaller openings or to remove the medial wall in dorsal direction in that way that the airflow is not directed into the maxillary sinus [211]. Based on the according anatomy, the secretion might be drained from the anterior ethmoid and frontal sinus into the maxillary sinus [274]. A larger maxillary sinus opening leads to a reduced concentration of nitrogen monoxide [287]. An association between large openings and recurrent infections or between reduced concentration of nitrogen monoxide in the maxillary sinus and resulting disease is not proven up to now [288].

The lymphatic drainage of the maxillary sinus mainly occurs via the mucosa of the natural ostium. That is why after surgery postoperatively new (!) mucosal swellings develop temporarily at the maxillary sinus ostium. In order to minimize those swelling, the mucosa should be preserved, for example at the anterior edge [289].

The transportation of coloring agents showed that in cases of severe disease of the maxillary sinus with accordingly disturbed mucociliary clearance the drainage might occur through an opening in the inferior meatus [290].

It is possible because of adverse anatomy that a relevant part of the maxillary sinus cannot be overseen despite the use of angular optics and that curved/angled instruments do not reach it via the enlarged opening in the middle meatus [291], [292].

If a complete removal of polyposis, a fungus ball, antro-choanal polyp, or other benign process via a middle meatal antrostomy is needed, the intervention has usually to be extended and an additional access must be chosen. There are several options:

##### 2.5.2.1 Extended middle meatal antrostomy (postlacrimal approach or grade 4 operation)

**Figure 1:**
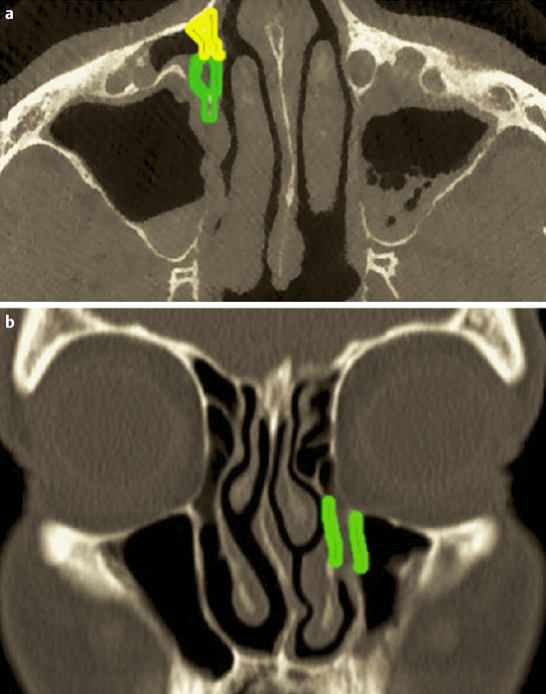
Extended maximal middle meatal antrostomy grade 4 (postlacrimal approach, [293]): Resection of the bone (green) medially, dorsally, and laterally of the nasolacrimal duct in order to mobilize it and to improve the insight into the maxillary sinus. Resection of the bone in case of prelacrimal access (yellow). a) axial CT scan, b) coronal CT scan.

Hereby the bony canal of the nasolacrimal duct is removed in medial, dorsal, and lateral direction, as well as the transition to the base of the os turbinale which is directly adjacent at the dorso-caudal part where mostly thicker bone is found (Figure 1 (Fig. 1)). In this way, the very robust nasolacrimal duct can be mobilized in anterior and medial direction and thus the insight into the anterior part of the maxillary sinus (pre-lacrimal recess, alveolar recess, anterior wall of the maxillary sinus) can be improved. In many cases, additional morbidity like numbness in the area of the infraorbital nerve due to transoral approaches may be avoided. In contrast to and to differentiate from the pre-lacrimal access, no separate anterior incision is performed at the lateral nasal wall (Figure 1 (Fig. 1)). This surgical step is a variation of the (partial) medial maxillectomy and clearly different from the middle meatal antrostomy grade 3. It holds immanent coding and reimbursement aspects. It is suggested to describe this surgery in continuation of the existing classification of grade 1–3 as fenestration of the maxillary sinus grade 4 or as postlacrimal approach (Figure 1 (Fig. 1), [293]).

##### 2.5.2.2 Fenestration via the inferior meatus

This approach has nearly been completely left in favor of the one performed via the middle meatus [279]. The insight into the maxillary sinus remains difficult also via this approach and the surgical options are limited. In cases of severe CRSwNP, the combination of inferior and middle meatal antrostomy could achieve improved surgical results which was interpreted as improved passive drainage and extended removal of the polyposis [234].

In individual cases, it seems to be reasonable to open and marsupialize a maxillary sinus mucocele via the inferior meatus, if previous surgeries had been performed and the topographic location is appropriate.

##### 2.5.2.3 Medial maxillectomy

Classical medial maxillectomy implies the resection of the inferior turbinate and the nasolacrimal duct beside the complete removal of the medial wall of the maxillary sinus [294], [295].

Therapy refractory maxillary sinusitis or dysfunctional maxillary sinusitis [210] obviously include the fact that the mucociliary clearance does not work satisfactorily despite surgically successful re-ventilation and further drainage via the middle meatus and that the maxillary sinus needs drainage depending on gravitation which is achieved by creating larger maxillary “windows” that also include the inferior nasal meatus in addition to the middle meatus which led to the development of different variations of medial maxillectomy [230], [231], [232], [233]. It is not clarified to what extent the hereby always mentioned partial resection of the inferior turbinate is necessary.

The preservation of the inferior turbinate may be important because of functional reasons. So alternative techniques allow temporary detaching and re-inserting of the inferior turbinate and thus its preservation if it is not affected by the disease process [296], [297], [298], [299]. Other variations of medial maxillectomy preserve the nasolacrimal duct [297], [298], [299], [300].

**Figure 2:**
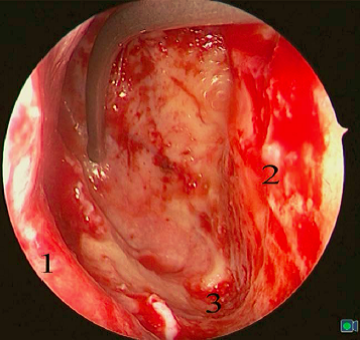
Prelacrimal approach: endoscopic view into the left maxillary sinus. The suction device points at the posterior wall of the maxillary sinus. 1 = nasolacrimal duct, 2 = anterior wall of the maxillary sinus, 3 = alveolar recess.

The pre-lacrimal approach to the maxillary sinus [301], [302], [303], [304], [305] allows both, a complete overview of the whole maxillary sinus, including the pre-lacrimal recess and all other recesses (applying optics with angled views and also angled instruments) together with the preservation of the inferior turbinate and the nasolacrimal duct (Figure 2 (Fig. 2)). It can be used as mere approach to the maxillary sinus, to the orbit, and to the retromaxillary space or it may be expanded to sound medial maxillectomy. The mucosa is removed from the lateral nasal wall with presentation of the os turbinale by placing an incision from the frontal process of the maxilla via the base of the inferior turbinate to the nasal floor. The base of the os turbinale is chiseled and usually the nasolacrimal duct is reached automatically. The duct is medialized and detached from its bony canal. Depending on the anatomy, the maxillary sinus is entered in front of or laterally to the nasolacrimal duct. The opening is enlarged step by step until the piriform aperture is reached, if needed also resecting parts of the anterior wall of the maxillary sinus, and the nasal floor until the complete maxillary sinus can be examined endoscopically. This procedure corresponds to former endonasal Denker’s surgery [306], [307] or Canfield-Sturman surgery, however with preservation of the inferior turbinate and the nasolacrimal duct. At the end of the surgical intervention the inferior turbinate is repositioned and fixed with 1–2 sutures. A sensation of numbness must sometimes be expected in the area of the terminal branch of the infraorbital nerve in up to 6.3% of the cases (Zhou 2014, publication in preparation).

##### 2.5.2.4 Transoral surgery 

##### Trepanation of the canine fossa (canine fossa trephine, CFT)

See [229], [274], [308], [309], [310], [311], [312], [313], [314]. 

Through a drill hole in the canine fossa, the pathological process is removed under endoscopic control, for example by means of 70° endoscopy via the middle meatus or the anterior opening, and if needed by using a microdebrider. Applying CFT, soft tissue processes in the maxillary sinus can be removed more rapidly and completely than via the middle meatus [228], [315]. In cases of dental maxillary sinusitis, the results were independent from the access via the middle meatus or CFT [316]. Temporary buccal swellings and numbness in the area of the infraorbital nerve are often observed [274], [316]. Persisting side effects must be expected in 0–3–5%, also in cases of optimized puncture technique (optimum target area: intersection of the horizontal line through the nasal floor with the vertical line through the middle of the pupil) and endoscopic control, especially numbness [228], [274], [313], [316], [317]. The temporary lesion of the buccal space of the facial nerve occurs very rarely [318]. Apparently, the development of the maxillary sinus is not impaired by CFT in children [319].

##### Caldwell-Luc surgery or osteoplastic surgery of the maxillary sinus

Currently only few indications exist for Caldwell-Luc surgery [317], [320] or osteoplastic surgery of the maxillary sinuses [276], [317], [320]. The canine fossa trephine and the post- and pre-lacrimal approaches have replaced this approach nearly completely.

If the usual landmarks are missing because of previous interventions, for example the nasolacrimal duct with the frontal process of the maxilla, the inferior turbinate, and the lamina papyracea with the orbital floor provide anatomical orientation [321]. The maxillary sinus

- can be opened via the posterior fontanel and the opening is completed in frontal direction to the nasolacrimal duct,
- can be depicted by exposing the nasolacrimal duct [83]. Scars, residual bony parts in the area of the natural or enlarged ostium can be clearly developed and removed, and the duct can be skeletonized if needed,
- can be opened directly via the base of the inferior turbinate and below the orbital floor with a curved instrument in an acute angle in caudal direction,
- single cases of maxillary sinus mucoceles for example after Caldwell-Luc surgery sometimes require an individual approach via the inferior turbinate.

The use of a navigation system may be helpful in complicated cases. The more dorsal the opening of the maxillary sinus is performed and the more caudal it is in relation to the inferior turbinate, the more probable is a lesion of a branch of the sphenopalatine artery [322] with associated bleeding – anticipating this event, the mentioned piece of mucosa may be coagulated as a precaution.

In most cases, a maximal enlargement of the maxillary sinus fenestration via the middle meatus requires the opening of the ethmoid bulla. This is part of anterior ethmoid sinus surgery.

As fenestration of the maxillary sinus is the most frequently performed intervention of the paranasal sinuses that is often not as simple as it seems [211], it must be emphasized that the essential first step is the identification and assessment of the natural ostium of the maxillary sinus by using optics with an angular view. This is the indispensible first step for enlarging the natural ostium if needed and to perform further surgical steps and to avoid the occurrence of a so-called “missed ostium sequence” (MOS) [16].

**Figure 3:**
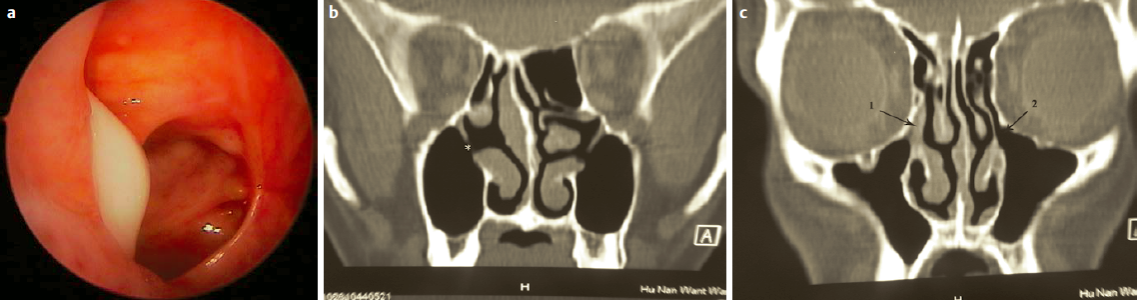
Missed ostium sequence. a) Typical secretion drop directly behind obvious remnants of the uncinate process in MOS of the right side after previous surgery. b) In the coronal CT scan a larger opening of the maxillary sinus is seen in the posterior part of the middle meatus (*). c) In the area of the natural ostium, however, remnants of the uncinate process and soft tissue are revealed (mucosal swelling, scars (=1) with obstruction of the natural ostium in contrast to free drainage on the left side (=2)).

MOS describes the situation that in dorsal direction of the natural maxillary ostium and anatomically separated a second opening to the maxillary sinus is created and that the obstruction in the area of the natural ostium leading to primary surgery was not removed. Because of genetic determination of the mucociliary transportation, the blockage of the mucosal transport out of the maxillary sinus remains leading to the classical clinical symptoms of recurrent acute inflammation, persistent mucus plug, or persisting secretion (Figure 3 (Fig. 3)). MOS is a negative predictor regarding the successful outcome of surgery [323] and it is often found in revision surgeries [16], [324], [325]. Usually, a partly preserved uncinate process, an infraorbital cell, scar tissue, and osteoneogenesis are found endoscopically or by computed tomography. Those finding have to be removed which is sometimes very difficult because of hard tissue. The obstruction of the natural ostium is considered to be the most frequent reason of persisting postoperative problems of the maxillary sinus, followed by residual disease of the ethmoid and/or frontal sinus and resistant bacteria [17].

In summary, modern endonasal endoscopic surgery of the maxillary sinus includes a nearly continuous spectrum of surgical interventions starting with the mere identification of the natural maxillary ostium via partial uncinectomy up to complete (classical) medial maxillectomy with enlargement by resecting the piriform aperture and parts of the medial anterior wall of the maxillary sinus and enlargement of the approach (operating angle) by transseptal approaches.

The nasolacrimal duct and the inferior turbinate can often be preserved (pre-lacrimal approach), apparently an impairment of the surgical success does not occur.

The more extended the intervention and the extent of bone resection is in direction of the nasal floor and the piriform aperture or the anterior wall of the maxillary sinus, the more frequent a lesion of the terminal branches of the infraorbital nerve or a externally visible depression of the lateral nasal base may occur.

A further improvement of the access to the maxillary sinus and the infratemporal fossa can be achieved by transseptal approaches [326], [327], [328], [329], [330]. The maximal endoscopic medial maxillectomy with resection of the nasolacrimal duct may lead to an additional range of instrumental action of an average of 20° [329]. A maximally enlarged access for rhino-neurosurgical indications is achieved by performing anterior maxillotomy with resection of the maxilla from the piriform aperture to the canine fossa [331].

#### 2.5.3 Ethmoid sinus surgery

Ethmoid sinus surgery starts with uncinectomy, whereby the ethmoid infundibulum is opened (=infundibulotomy). 

The next and first step of anterior ethmoidectomy consists of opening the wall of the ethmoid bulla most safely at the caudal medial part and removal of its wall in cranial direction and to the edges. If no supra-bullar recess is found, the skull base presents in cranial direction. If no retro-bullar recess is present, the basal lamella of the middle turbinate is depicted in dorsal direction.

**Figure 4:**
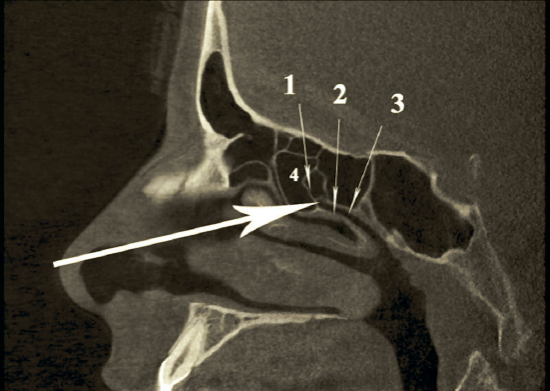
Sagittal CT demonstrating the surgical strategy to open the posterior ethmoid. After opening the basal lamella of the middle turbinate (1) directly above the horizontal part (2), the superior meatus (3) is reached. (4) = ethmoid bulla.

The posterior ethmoid sinus surgery starts with perforation of the basal lamella of the middle turbinate at the medial inferior part, directly above the horizontal part of the basal lamella (Figure 4 (Fig. 4)). The roof of the maxillary sinus is another helpful landmark for a safe surgical procedure. Remaining below the level of the maxillary sinus roof, a lesion of the dorsal ethmoid roof is actually not possible. It is recommended to previously analyze the topographic relation of the posterior roof of the ethmoid sinus and the roof of the maxillary sinus in the coronal CT scan. Furthermore, the preparation should be performed in horizontal anterior-posterior direction, for example in combination with a 0° optic.

After perforation of the basal lamella directly above its horizontal part, immediately the superior nasal meatus is reached. From the first opening, the basal lamella can be completely removed step by step and the few cells of the posterior ethmoid sinus can be exposed and removed if needed.

Attention must be paid to the presence of a spheno-ethmoid cell with possibly prominent or exposed optic nerve.

Afterwards, interventions of the sphenoid and the frontal sinuses may be performed.

#### 2.5.4 Sphenoid sinus surgery

The access to the sphenoid sinus can be performed by means of an exclusively trans-ethmoid, trans-ethmoid-trans-nasal, exclusively trans-nasal, trans-septal, or trans-pterygoid approach [332]. The individually most appropriate way is mainly determined by the individual microanatomy as well as the type and extent of the disease.

Important anatomical landmarks for safe opening of the sphenoid sinus are the natural ostium, the superior turbinate, the choanae, the nasal septum, the sphenopalatine artery, and the roof of the maxillary sinus.

In 98–100% the natural ostium is found medial to the base of the superior turbinate [333], [334], [335], [336], [337], [338], [339]. The distance to the choanae in caudal direction amounts to 21 ± 6 mm [338] or 2–15 mm [334]. The distance to the nasal septum in medial direction is only few mm, to the inferior edge of the posterior part of the superior turbinate is mostly less than 10 mm [334], [338]. Safe opening of the sphenoid sinus is possible at the level of the inferior edge of the preserved superior turbinate [337].

An imaginary parallelogram may help to find a safe way during transethmoidal sphenoidotomy: the medial vertical line is represented by the vertical lamella of the superior turbinate, the lateral line by the medial orbital wall. The superior horizontal line is represented by the skull base and the inferior line by the horizontal lamella of the superior turbinate. The best area for sphenoidotomy is the inferior-medial quarter of the parallelogram mentioned [336], [340].

The level of the medial roof of the maxillary sinus provides a safe orientation for presentation of the natural ostium of the sphenoid sinus. The roof of the maxillary sinus is always located inferior to the roof of the sphenoid sinus [341], [342]. A level at the height of the medial maxillary roof parallel to the nasal floor is located 2.8 ± 2.8 mm below the ostium and 12 ± 3 mm below the roof of the sphenoid sinus [343]. The opening on this level is performed in the lower third of the sphenoid sinus. The ostium is located nearly in the middle of the anterior wall of the sphenoid sinus and in cases of poor pneumatization it is nearer at the skull base [334], [344]. In 80% the ostium of the sphenoid sinus is slit-shaped and in 20% round or punctiform [334]. It can be securely palpated and penetrated 10–12 mm above the choanae with a blunt instrument [271], [334], para-septal and medial to the base of the superior turbinate.

Many authors resect few mm or the caudal third of the superior turbinate and consider this as unproblematic [275], [333], [340], [345], [346] even if a discrete interference with the sense of smell cannot be excluded theoretically [340]. There is just one scientific study addressing this problem. Resection of the inferior part of the superior turbinate (inferior third or fourth) turned out not to be associated with smelling disorder even if in a sixth of the specimens olfactory tissue could be found. On the other hand, no olfactory tissue was found in the specimens of all patients with relevant postoperative smelling disorder [347].

**Figure 5:**
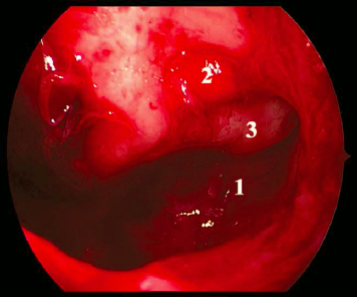
Transpterygoid approach to the left sphenoid sinus with view into the lateral recess (1), the maxillary nerve that is partly not covered by bone (2), and a part of the middle cranial fossa (3).

Regarding the choice of the approach, the following reflections have to be made, especially with the objective to perform safe and sufficient opening of the sphenoid sinus and to avoid strictly any endangering of the internal carotid artery or the optic nerve:

- The transethmoidal approach is useful in cases of broad contact surface between sphenoid sinus and posterior ethmoid and if ethmoid sinus surgery is performed at the same time. In this context, possibly also the branches of the sphenopalatine artery can serve as landmark by opening the anterior wall of the sphenoid sinus directly above or behind the posterior nasal artery (Daniel Simmen, personal conversation).
- The transnasal approach is often preferred in revisions and missing middle and superior turbinates [321], [334]. An intact middle turbinate is usually fractured and destabilized in transnasal procedures. The merely trans-nasal access may be difficult or even impossible in cases where the posterior nasal cavity is very narrow and crowded.
- In the context of the transethmoid-transnasal approach, the anterior wall of the sphenoid sinus is exposed via the superior meatus after transethmoidal perforation of the basal lamella of the middle turbinate and posterior ethmoidectomy. The opening is performed safely and the resection of the inferior part of the superior turbinate can be avoided if the removal of the anterior wall of the sphenoid sinus is performed consequently in an “L-shape” pattern on the left side and as a mirrored L on the right side (sphenoidotomy “around” the attachment of the superior turbinate). The middle turbinate is not lateralized and destabilized for this approach.
- The transseptal approach is recommended especially in cases of isolated diseases of the sphenoid sinus and/or narrow transnasal access and possible simultaneous septum surgery. Hereby the posterior part of the septum can additionally be removed in order to create a broad opening transnasally to the sphenoid sinus because the postoperative care may be rather difficult according to our experience. In analogy to surgeries of the maxillary and frontal sinuses, a “sphenoid drill-out” is described with removal of the complete anterior wall of the sphenoid sinus and the septum of the sphenoid sinus and the adjacent nasal septum in therapy resistant chronic sphenoid sinusitis [248], [348].
- In cases of straight septum and (nonetheless) narrow local anatomy, a transseptal approach via a hemi-transfixion incision or via a separate dorsal incision may be reasonable and less traumatizing than a transethmoid one. The postoperative care in the depth of a narrow nasal cavity is generally difficult which has to be taken into account when surgical openings are created.
- The transpterygoid opening of the sphenoid sinus is discussed for example for dural lesions or meningoceles in the lateral recess of a well pneumatized sphenoid sinus [349]. After the removal of the posterior wall of the maxillary sinus and exposition of the pterygopalatine fossa the content of the pterygopalatine fossa is lateralized and possibly preserved [350]. The bony delineation between the pterygopalatine fossa and the sphenoid sinus with the base of the pterygoid process and its medial lamella are removed until a sufficient exposition of the dural lesion is achieved and a preferred working with the straight optics (0°) is possible [351]. Transillumination of the sphenoid sinus can facilitate orientation. The foramen rotundum and the pterygoid canal represent important landmarks, the nerves should be protected [350], [352] (Figure 5 (Fig. 5)).

#### 2.5.5 Frontal sinus surgery

The particular difficulty of frontal sinus surgery is due to the complex anatomy of the preceding anterior ethmoid [12], [249], [257], [262], [269], [274].

The drainage pathway of the frontal sinus is formed by the cells of the anterior ethmoid which narrow or shift this pathway individually in very different ways (the following statements refer to actual nomenclature):

- The frontal sinus outflow tract is lined anteriorly by the agger nasi cell, which is the first cell in the frontal CT scan that depicts the key structure on the way to the frontal sinus [353], and the cranially above-lying frontoethmoidal cells [354], [355].
- Dorsally the ethmoid bulla is located and the bulla cells (supra bulla cell or frontal bulla; [256]).
- Medially there are the cells in the nasal and frontal sinus septum (intersinus septal cells).
- Laterally and posteriorly there is the supraorbital recess [356].

General landmarks for revision surgery of the frontal sinus are the frontal process of the maxilla, the lamina papyracea laterally, the roof of the ethmoid sinus posteriorly and possibly the non-affected healthy contralateral side [321].

According to recent refinements in terminology, all cells that narrow the frontal recess are named anterior ethmoid cells unless they do not reach into the frontal sinus itself. Otherwise they are called frontoethmoidal cells [12].

The frontal recess as drainage space below the imaginary “ostium” of the frontal sinus is delineated in dorsal direction by the ethmoidal bulla, in anterior-inferior direction by the agger nasi, in lateral direction by the lamina papyracea, and in inferior direction by the terminal recess of the ethmoid infundibulum (or it leads into the ethmoid infundibulum if the uncinate process inserts at the skull base or medially) [12].

The precise preoperative analysis of the anatomy and the drainage pathway of the frontal sinus by means of CT scan in three planes, e.g. using the box model with color coding of the pathway [274], [357], [358], facilitates the operative procedure. During surgery, the step-by-step technique consisting of preparing cell by cell according to the obvious gaps and clefts and removing them specifically, has been established as surgical technique. Removal (“scoopin out”) of the (mostly) last bony shell at the transition of the frontal sinus to the frontal recess was called “uncapping the egg” [247], [248], [268].

The classification of frontal sinus surgeries according to Draf with types I, IIa, IIb, and III has been internationally established [242], [248], [358], [359] even if some weak points and gaps of the concept have been identified because the anatomical variety of the anterior ethmoid and the frontal sinus are not sufficiently taken into account.

**Figure 6:**
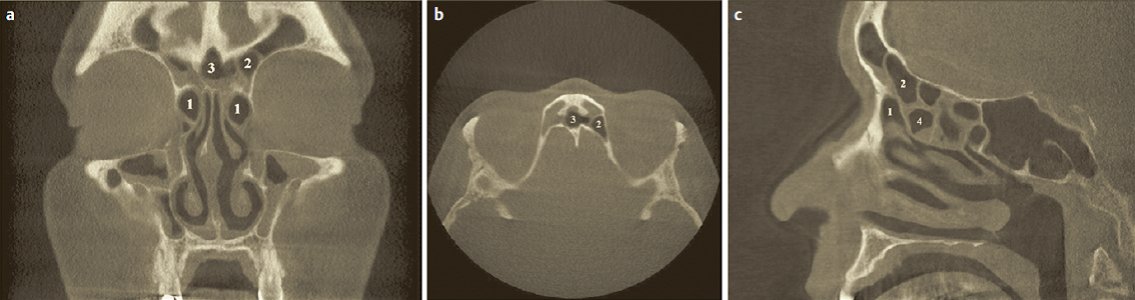
Frontal sinus drainage type I according to Draf = complete resection of the uncinate process and resection of parts of the medial lamella of the agger nasi cell and the anterior wall of the ethmoid bulla if needed [242, 246, 248, 359]. A different postoperative situation results depending on the individual anatomy: on the right isolated agger nasi cell, on the left side additional posterior frontoethmoidal cell (frontal bulla), intersinus septal cell; 1 = agger nasi cell, 2 = posterior frontoethmoidal cell (frontal bulla), 3 = interfrontal sinus septal cell, 4 = ethmoid bulla; a) coronal CT scan, b) axial CT scan, c) sagittal CT scan.

The definition of frontal sinus drainage type 1 is not clearly defined with relation to the extent of manipulations and to the expected results (Figure 6 (Fig. 6)). It is an intervention at the inferior border of the frontal recess and includes the complete resection of the uncinate process and if needed also the resection of parts of the medial lamella of the agger nasi cell and the anterior wall of the ethmoid bulla. Each further manipulation in the cranially located frontal recess should be avoided in order to prevent scarring. Depending on the insertion of the uncinate process and the number or configuration of the anterior ethmoid cells differently wide and configured drainage pathways result. This individual anatomy complicates an exact analysis of the performed resections including their influence on the drainage – especially if parts of the anterior ethmoid cells have been additionally resected [360].

According to the “all or nothing principle”, further partial surgeries in the frontal recess should not be performed [274], which means based on Draf’s classification that either frontal sinus drainage type I or type IIa is performed. The rationale is, that manipulations in the narrow clefts of the anterior ethmoid cells (may) lead to the development of scars and osteoneogenesis and thus the surgical objective is not only missed and, moreover, the postoperative situation might even be worse than the preoperative one. Even if there are no data on the incidence of iatrogenous postoperative frontal sinus problems [361], the significant incidence of postoperative disorders of the drainage in the “surgically touched” frontal recess as reason of revision surgeries seems to confirm the mentioned statement [15], [362], [363].

The frontal sinus drainage type IIa includes the removal of all above-mentioned ethmoid cells that impair the drainage. In the English literature, often the term of “frontal sinusotomy” is used, however, it is not clearly defined and corresponds most likely to frontal sinus drainage type IIa. At most thin pointed parts and ridges of the floor of the frontal sinus are removed with the frontal sinus punch. Care must be taken to preserve as much intact mucosa as possible in the “ostium area” in order to prevent stenosis due to scarring and osteoneogensis. For anatomic orientation, the agger nasi cell is considered as being a very important landmark [353], its medial lamella is often prominent (“vertical bar” [364]), and in dorsal direction there is the ethmoid bulla [365]. The special technical demands of a sufficiently frontal sinus drainage type IIa leads to the recommendation that only experienced surgeons should perform this intervention [361].

An improved access to the entrance of the frontal sinus can be achieved by punching down the attachment of the anterior middle turbinate at the lateral nasal wall, the so-called axilla. It corresponds to the anterior wall of the agger nasi cell (if present). The creation of a local medially pedicled mucosal flap (so-called “axillary flap” measuring about 8x8 mm) should lead to improved exposure of the frontal sinus entrance (96%, [366]) and to controlled scarring. The better the exposure is, the more easy is working with a 0° optic or a 30/45° optic which is more simple and associated with less failure than working with a 70° optic [367]. The “axillary flap” is repositioned at the end of the surgery around the middle turbinate [366]. Lateralization of the middle turbinate, synechia with the lateral nasal wall or an impossible endoscopic inspection of the frontal sinus ostium is observed in 14.5%, 11.6%, or 12% of the cases after 3–9 months. After more than 9 months the rates amount to 17.4%, 11.4%, or 12.7%, respectively [368].

If larger openings are necessary, this can be achieved by resection of the floor of the frontal sinus in medial direction and in the sense of a frontal sinus drainage type IIb in anterior direction. This procedure requires the resection of the anterior part of the middle turbinate in front of the level of the posterior wall of the frontal sinus and usually the application of a drill system [369], [370]. Only rarely, advanced frontal sinus surgery can be successfully performed only with punches [371].

A maximal opening of the frontal sinus, frontal sinus drainage type III (median drainage, “modified Lothrop procedure”, “frontal drillout”; [242], [359], [372]) is achieved by performing this surgical step on both sides and resecting at the same time the adjacent nasal septum and the septum of the frontal sinus (as far as possible). In cases of frontal sinus drainage type IIb and III, often the frontal process of the maxilla has to be removed (thinned put) as an additional surgical step. The surgical objective consists of creating a maximally wide access. The wound surfaces are usually not increased when the bone is thinned but the opening surface becomes disproportionally bigger! The limits of maximal resection are the external periosteum of the skin above the frontal process and the anterior glabella, in lateral direction the periorbit as well as possibly the dura, the frontal “T” (following resection of the superior nasal septum, the vertical arm of the “T” refers to the dorsally limiting lamina perpendicularis ossis ethmoidalis, both short arms correspond to the medial skull base/lamina cribrosa), and the first olfactory fiber or the anterior nasal artery as terminal branch of the anterior ethmoid artery in dorsal direction [242], [274], [373], [374], [375]. In any case, a smooth transition into the nasal cavity and the ethmoid sinus should result.

Different modifications are possible exceeding classical type IIa drainage and still not representing typical type III surgery with bilateral removal of the floor of the frontal sinus, the widest possible resection of the frontal sinus septum, and the resection of the adjacent nasal septum as well as including the resection of parts of the middle turbinate [242], [243], [359], [376], [377], [378], [379], [380].

**Figure 7:**
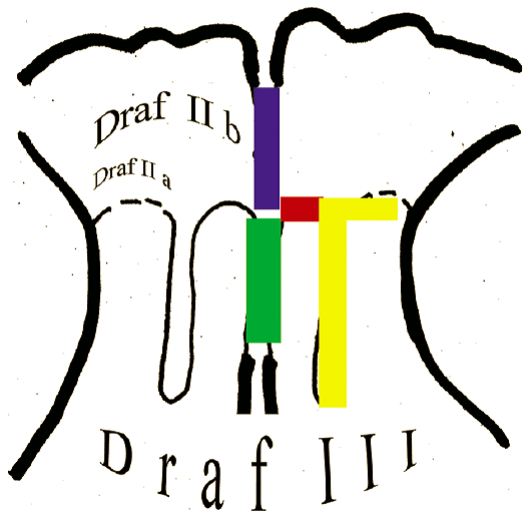
Extent of the resection of endonasal endoscopic frontal sinus drainage type IIa, IIb, III according to Draf. Type IIa = resection of all anterior ethmoid cells obstructing the frontal sinus drainage pathway. Type IIb = type IIa + resection of the ipsilateral floor of the frontal sinus + the ipsilateral middle turbinate in front of the level of the posterior wall of the frontal sinus. Advanced type IIb = type IIb + resection of the frontal sinus septum (blue). Modified type III = type IIb + resection of the nasal septum (green) (+ resection of the contralateral medial floor of the frontal sinus (red) + resection of the complete contralateral floor of the frontal sinus and the contralateral middle turbinate in front of the level of the posterior wall of the frontal sinus if needed (yellow) (if present) + resection of the frontal sinus septum (blue) if needed). Type III = bilateral type IIb + resection of the adjacent nasal septum + resection of the frontal sinus septum.

Isolated or also in a combined mode, resection of the anterior middle turbinate, the floor of the frontal sinus, the nasal septum, and the frontal sinus septum may be done or opted out in correlation to the individual anatomy and the type and extent of the disease (Figure 7 (Fig. 7)). In the literature, new names are coined for these procedures – currently a completely new classification does not yet exist. It is reasonable to define frontal sinus drainage type III via the resection of the nasal septum – only this resection allows a bilateral, unidirectional intraoperative working and a corresponding postoperative care. Complementation of the IIb drainage by mere resection of the frontal sinus septum may then be called an “advanced type IIb drainage”. Endoscopic frontal sinus surgeries going beyond type IIb and creating an endonasal transseptal bilateral access, may than be called type III surgeries. If they do not include the maximally possible drainage, the term of modified type III intervention would be appropriate. In all cases, the size of the opening should be mentioned in any operative report as significant measure of the surgical success and scientific questions [241], [274].

To overcome the limits of the classification system according to Draf we propose a modified classification of frontal sinus operations (= FSO) (Table 3 (Tab. 3)). Each FSO is different due to the resection of a defined and relevant anatomical structure. In this way this classification describes a complete and consistently step-by-step surgical approach to the frontal sinus.

Frontal sinus drainage type III can be performed, depending on the personal experience, the used instruments (0°, 30°, 45° optics; type of drilling system) and the individual anatomy, as anterior transnasal approach, via the depiction of the roof of the ethmoid sinus as trans-ethmoid approach, as transseptal approach, and via the contralateral side [242], [274], [373], [374], [381], [382]. The access from the anterior-inferior direction should allow a good overview of the surgical site and bear also timely advantages (“outside-in-approach”, [373]).

After completing type III drainage, covering of bony surfaces with thinned mucosa in the sense of free transplantations, e.g. of the nasal septum that has to be resected [383], [384], [385] or by positioning pedicled mucosal flaps [386], [387] seems to lead to a wider persisting neo-ostium and thus better surgical results [388]. Additionally, this leads to a significant reduction of the postoperative morbidity of the patients and a relevantly facilitated postoperative care for the patient and his physician, especially when it is combined with a so-called occlusive postoperative treatment [384]. The wound heals more rapidly, less crusting is observed, painful treatments can be minimized [384], [389], [390].

In 85–92% the frontal sinus “neo ostium” remains open after frontal sinus drainage type IIa [361], [362], [391], [392], [393]. Its size may be reduced naturally to 31% because of wound healing processes during the first 12 months [282] or within 6 months to 65% [394]. Whereas after one year the accesses were open in 90% of the cases, the patency rate decreased to 67% after 6 years [395]. The probability of postoperative stenosis decreases with the initial size of the intraoperative opening [396]. A diameter of around 5 mm is considered as critical as the rate of stenosis significantly increases [361], [396]. An important correlation exists between the rate of stenosis and the size of the frontal sinus drainage if it measures in width or depth less than 2.7 mm [361]. An increased shrinking was further observed in CRSwNP and intolerance to analgesics [8], [361], [396].

Patients with obstructed ostium and residual disease complain more often from symptoms and persisting infection [361]. Asthma, CRSwNP, advanced disease (Lund-Mackay score >16), and an obstructed frontal sinus ostium (<4 mm) are additional negative predictors regarding surgical failure in cases of frontal sinus drainage type IIa and the necessity of revision surgery consisting of frontal sinus drainage type III so that in those cases it must be discussed if primary frontal sinus drainage type III is appropriate [8].

At the same time this means that a narrow frontal sinus ostium is not (mandatorily) a contraindication for frontal sinus drainage type III, morover: anticipating the arguments mentioned, it may specifically justify this procedure.

Numerous factors influence possible re-stenosis: the underlying disease (CRSwNP, CRSsNP, revision because of scarring, revision because of preserved frontoethmoidal cells), preservation of mucosa under modern optimal conditions (HD visualization, cutting instruments etc.), and postoperative care.

Frontal sinus drainage type III is most often applied in cases of chronic rhinosinusitis, followed by mucoceles, more rarely in traumatology and tumors, especially in rhino-neurosurgery [245]. The data of a meta-analysis revealed a rate of long-term open accesses of 81%, a rate of stenosis of 15%, and a rate of complete obstruction of 4%, an improvement of the symptoms in 82%, and a revision rate of 14% (among those type III revision was performed in 80%). As this data material is very heterogeneous, it can only provide a rough result. Detailed analyses show very different data:

- The rates of open accesses varies between <70% and 90% [8], [240], [241], [243], [359], [372], [384].
- The rates of revisions varies between 5% and 32% [240], [241], [242], [359], [397], [398], [399].
- The rates of clinical success are more stable with improvements around 80% [241], [243], [244] and can be compared to those of osteoplastic frontal sinus surgery [400].

On the one hand, there is a general tendency of shrinking of around 30% within the first year [384], [401] that increases in the second year and should stop afterwards [241]. On the other hand, in a major part of the patient the access remains open whereas significant stenosis of 50–60% occurs in 30–40% of the patients requiring revision in 20–40% of the cases [401], [402], whereby the reason is actually unclear. Impairing factors are allergic fungal sinusitis [241] or eosinophilic rhinosinusitis [401], surgery of tumors or mucoceles [403]. The intraoperatively created dimensions of the neo-ostium significantly influences the postoperative neo-opening and thus the rate of re-stenosis [401], which may be an explanation for the clearly poorer success rate if a smaller intraoperatively opening is created (revision rate of 30%, extent of the general stenosis of 47%; [403]). Apparently those reflections are not true for a current series of Chinese patients. The very large intraoperative opening shrank by 50% after one year, and the percentage of neo-osteogenesis contributing to the reduction of the opening surface amounted to 21% [404].

Part of the patients with obstructed frontal sinus access remains without further complaints [401], [402] so that this fact alone does not automatically justify revision surgery.

The objective of surgically performed frontal sinus drainage type III must be to anticipate the non-predictable significant tendency of stenosis in about one third of the patients. This means:

- A maximal opening must be created [401]. This maximized concept replaces former recommendations of target neo-ostia measuring of at least 16x8 mm [397], 15–20x10–15 mm [242] (width x depth) or 10 mm [405] (anterior-posterior).
- The development of granulations and scars must be minimized by covering the bare bone with mucosa (free transplantations or pedicled flaps; [383], [384], [386], [387], [388]).
- The conditions for wound healing must be optimized during postoperative care (see chapter on postoperative care) [242], [384], [389].

Regarding the optimum extent of surgery of the frontal recess and the frontal sinus there are different actual concepts starting with very reluctant interventions that mainly focus on the middle meatus, based on the principle of FESS [182], [184], [195], and ending with consequent performance of frontal sinus drainage type IIa when a proven pathology of the frontal sinus remains after drug therapy [24], [248], [361]. Current data indicate that an early, perhaps even primary frontal sinus drainage type III may be favorable in selected patients with advanced chronic inflammatory diseases [8], [24].

For better visualization of the drainage pathway of the frontal sinus during endoscopic frontal sinus drainage type IIa or type III, several authors perform frontal trepanation (“frontal trephine”) with application of fluorescein solution in the frontal sinus to color-code the drainage pathway [274], [318], [366], [392], [406], [407], [408], [409]. Another advantage of the minimized additional external acces may be that for some days postoperative rinsing can be performed, for example with cortisone solution [410]. Frontal trepanation can also be performed to allow surgical manipulations [411], [412]. In the line between the medial eyebrow delineations, a stab incision of the skin is performed 1 cm paramedian, a drill-hole is created, and a cannula is inserted. The careful analysis of the preoperative CT scan regarding sufficient pneumatization of the frontal sinus and possible bony dehiscence to the orbita is required. Complications occur in 6% of the cases, most frequently local infections [406]. Dura lesions and the jetting of the frontal sinus content into the orbita are described as rare complications [406], [413]. The first author himself has never performed this technique.

Alternatively, a puncture and mini-endoscopy via a small opening of the anterior wall of the frontal sinus was described in cases of difficult anatomical orientation during primary frontal sinus surgery or during surgery for mucoceles after external previous surgery in order to identify the optimal endonasal area to open a completely obstructed frontal sinus or to demonstrate the physiological drainage [414], [415].

The complementary application of intraoperative navigation is recommended in particular in cases of difficult anatomical situation in revision surgeries [416], [417].

If the frontal sinus access is stenosed or obstructed by a lateralized middle turbinate, the lateral part of the mucosa of the middle turbinate can be mobilized and preserved. After resection of the lateralized turbinate this flap is positioned on the resection surface in medial direction to the skull base in order to achieve optimal epithelialization of the frontal sinus opening (“frontal sinus rescue procedure”, [412], [418], [419]). An extension (“extended frontal sinus rescue”) could be a variation which is performed in cases of lateralized middle turbinate with obliteration of the frontal recess [43]. The turbinate is preserved caudally but the opening through the turbinate near the skull base is created and the same procedure is applied. Generally, this approach can be recommended, however, it is technically very demanding and challenging. Success rates of 56% after one, 78% after 2, and 91% after 3 interventions have been reported [412].

Frontal sinus mucoceles after fat obliteration of the frontal sinus can undergo endonasal endoscopic surgery by means of frontal sinus drainage type II-III, if the mucocele can be easily reached from an inferior approach (“frontal sinus unobliteration”, [410], [420], [421], [422]). To guarantee a permanently open drainage and regular epithelialization, today the coverage of bone with mucosal transplantations is recommended in addition to maximizing the opening [410], if needed with the additional insertion of soft silicone foils [42]. The clinical success rate after such (type III) surgery is given with around 90% [410].

**Figure 8:**
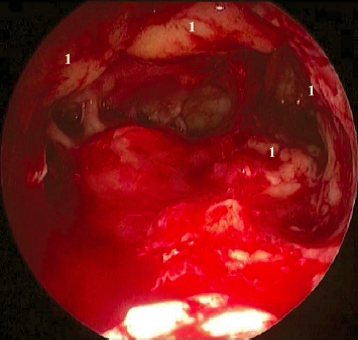
Condition after frontal sinus drainage type III with maximal opening, smooth transition and coverage of the bare bone with free mucosal transplants (=1)

In summary, also endoscopic frontal sinus surgery encompasses a meanwhile nearly continuous spectrum of surgical techniques starting with improvement of the drainage at the inferior border of the frontal recess up to maximally possible enlargement of the drainage opening as frontal sinus drainage type III. In single cases, small accesses from the outside as frontal trepanation might be useful or necessary in order to complete or secure the achievement of the surgical objective. Technical developments are required in order to overcome existing deficits regarding overview, insight, and manipulations in the frontal sinus [423], [424], [425]. Few cases still represent an indication for an external approach [275], [276], [359], [426]. Osteoplastic surgery with fat obliteration is considered as *ultima ratio* if permanent ventilation and drainage cannot be realized despite frontal sinus drainage type III revision [359], [403], [427], [428] (Figure 8 (Fig. 8)).

#### 2.5.6 Middle turbinate

The middle turbinate is an important landmark in endonasal sinus surgery. A normal middle turbinate should not be routinely reduced [429]. If the middle turbinate is part of the disease (e.g. polyposis, “osteitis”) or if it apparently impairs surgery or the outcome because of its size (e.g. concha bullosa) and shape (e.g. lateralization, paradox curving), this diseased part should be resected [429]. 

The question if and when a (partial) resection is appropriate, required, or allowed, can only be answered individually.

Numerous retrospective [430], [431], [432], [433], [434] and prospective [238], [239], [395], [435], [436], [437], [438], [439], [440] case series at least draw the conclusion that partial resection of the middle turbinate does not impair the nasal function and does not cause intra- and postoperative complications (however, more orbita and dura lesions are described in [441]) and the surgical outcome is not poorer but rather better [238]. A possible and perhaps very rare development of empty nose syndrome by partial resection of the middle turbinate is not definitely clarified [442], [443], [444]. 

Preferential development of postoperative frontal sinusitis (75% vs. 45% in a retrospective case control study, CRS or RARS, [445]) could not be verified in other studies [432], [435], [439]. Olfaction is not impaired [237], [438].

Prospective randomized studies reveal less synechiae, a lower rate of revisions or less postoperative complaints, and improved patency rates of the maxillary sinus ostium if the middle turbinate was partially resected [237], [446].

Thus it seems to be sufficiently secured that partial (anterior-inferior) resection of the middle turbinate with preservation of landmarks function is at least acceptable. It may be a surgical alternative to techniques of medialization in cases of extended traumatization [429] and even under narrow anatomical conditions it appears to be appropriate [429], [447]. Studies indicate improved therapeutic results in advanced disease without the risk of complications in the sense of empty nose syndrome [225], [237].

#### 2.5.7 Lateralization/medialization of the middle turbinate

Lateralization of the middle turbinate with potentially undesired obstruction of the accesses to the maxillary sinus, anterior ethmoid and frontal sinus because of scarring due to wound healing [389] occurs in 10–40% [448]. It can impair the surgical outcome and is often mentioned as reason for necessary revisions [15], [197], [362]. Measures and techniques to avoid or reduce those aspects are:

- Atraumatic, “non-contact” surgical procedure, preservation of the medial lamella of the ethmoid bulla and the horizontal part of the basal lamella
- The partial resection in anterior-inferior direction of the middle turbinate can be performed without expecting negative consequences [429], [433], [436], [440], [449].
- Transseptal suture [368], [450], [451], [452], [453]. The medialization by means of transseptal suture reduces the extent of synechia between the turbinate and lateral nasal wall (3% vs. 11%) and significantly reduces the incidence of lateralization (5% vs. 16%; [368]). Applying the axillary flap technique, however, the transseptal suture did not influence the incidence of lateralization of the middle turbinate [368]. In 85% to more than 95% it is described as successful [368], [451], [452], [454], [455], [456]. Synechiae between the middle turbinate and the lateral nasal wall were observed in 5–12% of the cases [454], [457], undesired medial synechia occurred in single cases. An impaired olfaction due to medialization could not be observed [452], [458], [459], however, it seems to be possible in single cases.
- The targeted injury and induced development of scars between the middle turbinate and the nasal septum (“Bolgerisation”) [458], [460], [461].
- Fixation with clips [455] or the insertion of absorbable implants [462].
- So-called “relaxation incision” of the basal lamella between the sagittal and frontal level is expected to enlarge the middle meatus and to facilitate the medialization of the middle turbinate [463].
- Medialization by means of packing materials in the middle meatus or ethmoid would only be successful if they remain for several weeks (!) [461], which cannot be recommended [464].

Crushing of the concha media bullosa must be considered as insecure procedure regarding its short- and long-term effects [465], [466]. Aspects like residual or newly occurring inflammatory foci in the crushed concha, insufficient realization of free drainage of the anterior ethmoid and frontal sinus have not been investigated sufficiently up to now.

In contrast, endoscopic turbinoplasty with resection of the lateral part of the middle turbinate leads to synechiae medially only in 6% and laterally only in 2% [467]. 

#### 2.5.8 Synechiae in the middle meatus/stenting in the middle meatus

The prophylaxis of synechiae in the middle meatus seems to be important as the presence of synechiae or of a lateralized middle turbinate was correlated with a poorer surgical outcome [454], [468], [469]. However, it is still not clarified if the poorer outcome was a consequence of drainage-impairing synechiae, a consequence of a hereby caused less sufficient topical therapy, or the expression of a generally more advanced disease or at least in less advanced cases only of “cosmetic” nature [468]. The according implication for surgical therapy would then be very different.

Synechiae in the middle meatus and lateralization of the middle turbinate are often associated so that measures and techniques to avoid lateralization of the middle turbinate are generally also appropriate for prophylaxis of synechiae. The following procedures are described especially for prophylaxis of synechiae:

- An appropriate postoperative care is useful to reduce the incidence of synechiae (see chapter on postoperative care, [470]).
- It is effective to insert a molded tamponade for 5 days [471] or a splint for 10–14 days [472]. Other authors recommend an application of tamponades for up to three weeks with PVA tamponade in smooth sheath [461], [473].
- According to a series of studies, the insertion of stents or special materials/systems/spacers with or without drugs should be effective [464], [474], [475], [476], [477], [478], [479], [480], [481], but a clear evidence could not be given – except in one study (see below).

Generally, a prolonged local anti-inflammatory therapy in the middle meatus for reduction of postoperative undesired reactions and for therapy of the underlying disease of CRS is reasonable and preferable. At least an inert carrier medium is necessary in addition to an anti-inflammatory substance that is integrated into the system and pharmacokinetic conditions that allow predictable and clinically appropriate drug application. Currently such a system does not exist, many questions on this issue are still not clarified.

There are currently 3 meta-analyses on the application of absorbable/non-absorbable stents/spacers in the middle meatus that should avoid synechiae and influence the postoperative course [474], [475], [480]. Spacers tend to be considered as more appropriate, especially non-absorbable ones (only one included study!) [474]. Differences between non-absorbable and absorbable materials were not found unless the absorbable material had been left in place for at least 2 days [480]. Regarding the assessment of cortisone-containing spacers, there was no sufficient evidence [480]. Two prospective studies and one summarizing meta-analysis were published on a grid of absorbable polysaccharide polymer matrix with cortisone impregnation that should be inserted into the middle meatus/ethmoid entrance [475], [482], [483]. The meta-analysis with positive results, however, only states that the grid-shaped stent with cortisone impregnation leads to better results than the same stent without cortisone (adhesions, lateralization of the middle turbinate, incidence of revision, recurrent polyposis). A possible alternative interpretation of the data could be that an increased tissue reaction on the foreign body, the stent, (edema, granulation etc.) was balanced by the additional corticosteroid, an aspect that would explain the significant effect, whereby the proof of superiority regarding a best alternative remains open. A randomized comparison of interventions without stent does not exist. During the follow-up, a topical therapy with cortisone applied as spray or rinsing was not admitted. The general benefit or a possible detriment are not clarified [484]. Furthermore, the question is not clarified if the frontal recess and the entrance of the frontal sinus that cannot be accessed because of long-term spacers obstruct more or less often by scars.

It must be mentioned that in all publications on those stents the authors mentioned a financial interest regarding the manufacturer.

The assessment of stents to keep the entrance of the ethmoid open, to avoid lateralization of the middle turbinate, and to reduce adhesions and synechiae is difficult and the present meta-analyses are not sufficiently helpful because a significant part of the materials included in the studies are generally not recommended because of unfavorable side effects [464]. At the same time, the materials are different to an extent that a meta-analysis is problematic [474], [480].

The only material that is known up to now to reduce synechiae in the middle turbinate in randomized studies and that turned out to be effective in all in-vitro and in-vivo studies is a chitosan dextran gel [282], [485], [486], [487]. Confirmation by other study groups would be desirable.

Local therapy of CRSwNP or of recurrences by means of cortisone-eluting systems/stents is described in single publications [488], [489], [490], [491], [492], however, many questions still have to be clarified (topical or systemic effect? Extent of absorption?) [490], [492] before a sound recommendation can be given.

Another system for local cortisone application led to injury of the orbita in one case caused by the ethmoid implantation inserted in the ethmoid bulla. Another system was accidentally left in place [493], [494].

#### 2.5.9 Mitomycin C

The topical application of Mitomycin C (= MMC) reduces the risk of synechiae in the middle meatus and stenosis of the maxillary sinus opening in the middle meatus by 70% within a follow-up interval of less than 3 months [394], [495], [496], [497], [498], [499], [500], [501], [502], [503]. Especially patients below the age of 40 and patients undergoing first surgery benefit from the application. A long-term effect is not known. Up to now, no severe side effects have been described [500], [502], however, there are no correlating long-term data [500]. The recommendation consists of applying a solution of 0.4–0.5 mg/ml for 5 minutes and subsequent rinsing of the application site with saline solution [500]. A repeated application (done in rabbits after 3 days [504] and in humans after 4 weeks [499]) may enhance the effect. In the area of frontal sinus surgery, a positive outcome in a non-controlled study was reported [505].

A routine application, however, does not seem to be indicated despite the positive results of a meta-analysis because long-term data are missing and possibly unknown risks may appear [506]. At the same time, the absolute risk of major stenosis of the maxillary sinus opening seems to be low if the appropriate surgical technique is applied (see chapter on outcome). Regarding synechiae in the middle meatus, a series of alternative techniques are at disposition (see chapter on middle turbinate).

### 2.6 Special aspects of endonasal endoscopic sinus surgery

#### 2.6.1 Ergonomics

It is recommended to check the camera orientation on the monitor and macroscopically every now and again in order to avoid and correct accidental rotation and resulting false orientation. It is useful to have always a camera orientation in upward direction.

Also ergonomic aspects should be considered, especially in long lasting interventions [507]. According to the experiences from laparoscopic surgeries, the following recommendations are mentioned among others:

- The screen should be positioned in a distance of 80–120 cm (in case of big screens perhaps more) directly in front of the surgeon, in lateral direction, the angle may amount to 15°, downwards up to 20°.
- The table height should allow that the hand is in a similar level as the elbow (± 10 cm).
- The arms should be a little abducted and inwardly rotated.
- Flexion, bending, and rotation of the wrist should amount to less than 15°.
- Surgeons who stand should slightly lean at the table and use gel pads or other ergonomic pads if needed.
- Unnecessary devices with pedals should be avoided and manually controlled instruments should be preferred.

#### 2.6.2 4-hands-technique

The 4-hands-technique does not only allow the surgeon to work bimanually while the assistant controls the endoscope and the suction device [508], [509], [510]. The concept of “two minds” expresses the complementary competence of two surgeons working together [83], [247], [509]. A reduction by 21% of the duration of surgery could be achieved in the surgical therapy of CRS [509]. Especially for endonasal tumor surgery and generally for extended endonasal sinus surgery the 4-hands-technique is not only useful but often it is necessary.

#### 2.6.3 Endonasal endoscopic sinus surgery and rhinoplasty

The simultaneous performance of rhinoplasty and endonasal sinus surgery is considered as being safe and effective [511], [512], [513], [514]. The more extended the intervention or the more advanced rhinosinusitis is, the higher seems to be the risks the patients would have to be informed about in the individual case. The present literature, however, does not confirm the theoretical objections like for example poorer esthetic outcome, increased rate of postoperative bleeding, difficulties in finding the location of bleeding, masking of orbital complications, insufficient instruments for local postoperative care.

A prolonged postoperative swelling may be expected in the area of the nasal tip and the nasal bridge. In advanced rhinosinusitis, the infection risk seems to be higher, whereby the definition of “advanced” is not clear and in particular it applies for purulent rhinosinusitis.

Sinus surgery should be performed before rhinoplasty.

#### 2.6.4 Exostoses of the paranasal sinuses

**Figure 9:**
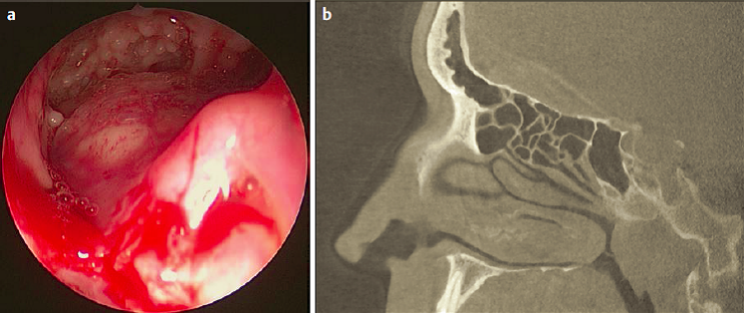
Exostoses of the frontal sinus, endoscopic view (a) and CT scan (b)

Exostoses of the paranasal sinuses have been described in patients after sinus surgery of the maxillary sinus, ethmoid sinus, and sphenoid sinus [515], [516]. In analogy to exostoses of the auditory canal, they are considered as consequence of nasal rinsing with too cold water. This stands in contrast to a case observed by the first author with exostoses of the frontal sinus that were diagnosed in the context of initial surgery of the paranasal sinuses and that had not developed because of local cold stimulus (Figure 9 (Fig. 9)).

#### 2.6.5 Endoscopic sinus surgery and growth of the facial skull

While endoscopic sinus surgery does not lead to growth disturbances of the infantile facial skull [517], [518], ethmoidectomy in adults may lead to narrowing of the ethmoid cavity that amount to >2 mm and that depend on the extent of surgery [519] (and probably on the degree of aggressive removal of the mucosa).

#### 2.6.6 Cleaning/rinsing systems for endoscopes

The problem of soiling of the tip of the endoscope is technically met, for example by mounted cleaning systems (Endoscrub^®^, Clear vision^®^, sleeve technique, K-endosheath [520], [521]). The advantages of cleaning the device in situ must be weighed out against the limitations: success rate of cleaning <100%, meanwhile necessary cleaning of the rinsing system, purchase and non-recurring costs, increased set-up time, increased thickness of the endoscope with possibly reduced flexibility. In general, the application of those systems is more useful and also necessary, the more and intensive the expected contaminations are, as for example in intensive drilling, tumor surgery etc.

#### 2.6.7 Heat development by endoscopes

Endoscopes develop heat, mostly at the tip [522], depending on the diameter and type of optic (0°, angular optic) as well as the use of the source of light [522], [523], [524], [525]. Up to 95.5°C have been measured (30° endoscope 4 mm, 300 W Xenon 100%) or in 33% light intensity of a 300 W Xenon source of light 44.3°C (0° endoscope 4 mm) [525]. The light cable at the outlet becomes even hotter [523], [524]. In a distance of 5 mm, the body temperature is no longer exceeded. After turning off the light source, the temperature also decreased below body temperature within 2 minutes [525], [526]. Cooling can be achieved by using saline solution rinsing, suction devices, or endoscopic sheaths [525], [526].

It is recommended to avoid direct tissue contact with the tip of the endoscope and to consequently cool by suction and/or by rinsing near critical (neuro-vascular) structures [525].

#### 2.6.8 Histological examination

The routinely performed examination of specimens taken from sinus surgeries is recommended by nearly all authors [448], [527], [528], [529], [530], [531], [532], [533], [534], [535], [536] because

- histologic parameters (e.g. eosinophils, neutrophils, extent of the inflammation) may be relevant for prognosis and therapy [533], [537].
- in up to 1.1% of the cases another diagnosis is found than pre- or intraoperatively suspected which significantly influences therapy. Most frequently, those are inverted papillomas, more rarely systemic inflammations and malignant tissue growth. Irrespective to the fact, that there is no answer to the question, if there is an influence on the outcome, in patients revealing small (!) lesions that are found incidentally and could not allocated topographically [538], those patients need clinical control in narrow intervals in order to early identify for recurrent tumor growth and to treat it adequately.

The incidence of unexpected histological findings in endoscopic sinus surgery for CRSwNP is given with 0.36% and for financial reasons routinely performed histological examinations are questioned [539]. This point of view, however, neglects the significance of histological parameters for the therapy of the underlying disease.

Special attention is required in case of unilateral findings, endoscopically suspect appearance, and bleeding events [528], [531], [536], [539].

The use of a shaver does not impair histological examination provided the tissue is collected [540], [541].

#### 2.6.9 Reimbursement issues

The current reimbursement catalogues do not mirror the state of modern endoscopic sinus surgery so that an appropriate discussion is not possible. It is required to adapt the honoraria to the significantly improved and time- and cost-intensive equipment of endoscopic sinus surgery which should lead to an according increase. Standard HD video endoscopy was not listed in the original calculation. The expenses (set-up time, purchase and maintaining costs, single use material) for the application of special devices and instruments (navigation, shaver, balloon, special nasal packing) must be taken into consideration. Extensive interventions with higher technical and time-consuming, sometimes staff-related, expenses (4-hands technique) require an additional significant remuneration.

#### 2.6.10 Outpatient or inpatient sinus surgery

The vast majority of interventions is performed on an inpatient basis, surgery itself is mostly performed under general anesthesia, with only few exceptions [542], [543], [544], [545]. Only 17% of the patients would have preferred to go home after surgery [544]. In 7–9% of the cases, relevant perioperative bleeding must be expected [544], [546].

In cases of outpatient sinonasal surgery of selected patients, unplanned inpatient admission occurred in 0.8–2.65% [543], [547] or need for emergency care in 5%. The rate of inpatient re-admission after septoplasty and possibly simultaneously performed turbinate surgery amounted to 5–13.4% [548], [549], [550]. The most frequent reasons were pain, bleeding, fever, and nausea [542], [546], [547], [551].

For outpatient performance of endonasal endoscopic sinus surgery, most likely patients are considered with low anesthetic risk [544], [552], [553] in combination with a planned intervention of little extent and duration [544], [553], [554] and without particular risk factors. Social factors and the possibility to quickly reach a hospital in an emergency, must also be considered.

Argumentation in favor of inpatient admission may consider that possible complications occurring in the postoperative course with threatening emergency situations can be treated immediately:

- Dural lesion with the risk of intracranial infection. Indicating symptoms are for example severe postoperative headaches or changes of vigilance, severe nausea and vomiting.
- Orbital phlegmons with the risk of blindness that may occur with and without orbital injury. The authors knows of 3 cases of foudroyant bacterial inflammation during the first postoperative night that required immediate action.
- Orbital hematoma with the risk of blindness.
- Postoperative bleedings from branches of the ethmoid and sphenopalatine arteries.

It must be called back into mind, that even severe complications are often not recognized intraoperatively by the surgeon.

Vasovagal reactions occurring in outpatient endoscopic interventions must be expected in 0.16% of the cases [555].

## 3 Complications

Type and incidence of complications in the context of endonasal sinus surgery, influencing factors, avoiding and treatment of arising complications, aspects of process and structural quality as well as training and medico-legal questions have currently been described in an encompassing way [448].

Endoscopic sinus surgery can be considered as safe surgery with a rate of 0.5–1% of severe and 5–7% of light complications [448], [556].

The rates of severe complications are lower in the context of endoscopic technique compared to microscopic or classical endonasal surgery [557].

The complication rates in pediatric endonasal sinus surgery are significantly lower according to reports on cohorts with no skull base lesions in >3,000 interventions [558], [559].

Regarding an increased complication rate, the following risk factors are mentioned [448], [556], [558], [560], [561]:

- Low conceptual/manual expertise of the surgeon,
- Extent of the intervention/the disease,
- Previous interventions (not mentioned in [558]),
- Existing comorbidities,
- Higher age (>40 years [560], >65 years [558]),
- Anatomic abnormalities/missing landmarks,
- Increased intraoperative bleeding,
- Surgery of the right side (in case of right-handed surgeons),
- Use of a navigation system which was explained by the situation that the navigation system was possibly applied in difficult cases or led to a false intraoperative sense of security [558],
- Insurance status (Medicaid, USA [558]).

Especially severe complications were often described for experienced surgeons [562], [563]. A low incidence of complications for more experienced surgeons [7], [553], [564] was expected to be due to an increased, however not sufficiently matured expertise.

For minimization of complications the following measures are recommended among others [448], [565], [566], [567]:

- Precise anamnesis regarding known tendency of bleeding, drug intake, previous operations with possible complications, accidents,
- Precise anamnesis regarding vision and olfaction, possible documentation,
- Previous treatment in cases of advanced inflammation for improvement of the intraoperative site (see chapter on previous treatments),
- Precise analysis of the CT scan/CBT in order to identify risk situations (see chapter on preparation of the surgery site),
- Exploit the perioperative and anesthesiologic measures to optimize the intraoperative site (see chapter on preparation),
- Identify appropriate surgical techniques (working alongside anatomical structures such as the lamina papyracea or skull base and other landmarks, avoiding of anterior-posterior penetration of bony septa),
- Secure removal of the uncinate process without penetrating the orbit,
- Penetration of the basal lamella of the middle turbinate in inferior direction and medially directly above the horizontal part of the basic lamella. It is recommended to orientate both the endoscope (0°) and the instruments in a horizontal plane parallel and inferior to the roof of the ethmoid. The superior nasal meatus is directly reached,
- Secure mobilization and punching of bony lamellas,
- L-shaped enlargement of the sphenoid sinus ostium on the left side (or mirrored L on the right side).

## 4 Indications of surgery beyond chronic rhinosinusitis

### 4.1 Indications of surgery in acute rhinosinusitis

- Persistence or increase of acute complaints, especially headaches and/or facial pain, fever, purulent secretion and/or disturbed general condition despite sufficient intravenous antibiotic therapy [568]. This concerns also isolated acute sinusitis of single paranasal sinuses,
- Impending or definitive complications (orbital, endocranial, involvement of cranial nerves, migration) despite maximal drug therapy; in those cases the indication for surgery becomes urgent,
- Acute sphenoid sinusitis with accompanying impaired vision requires intravenous antibiotic therapy and emergency surgery [569], [570], [571], [572].

#### 4.1.1 Orbital complications of acute rhinosinusitis

- Orbital edema (“preseptal edema”, “periorbital inflammation”, Chandler I and II; [573]) with normal vision that does not improve or even increases after 24–48 hours with sufficient (intravenous) antibiotic therapy [19], [574].
- Subperiostal abscess (Chandler III) with normal vision unless a conservative approach is not chosen having in mind the below-mentioned exceptions.
- In cases of primarily conservatively treated subperiostal abscess: urgent need for intervention is given when the complaints do not improve within 24–48 hours and also if any deterioration is observed [19], [575]. Immediate surgery is needed in any case of reduced vision, intraocular pressure elevation, or ophthalmoplegia [575].
- Intraorbital abscess (Chandler IV) or orbital phlegmons. Hereby urgent indication for surgery is established. For surgery of the affected paranasal sinuses, the periorbita is endoscopically slit for pressure relief and drainage of the inflammation. If needed the abscess is relieved and perhaps a drainage tube is inserted [576]. A medially located orbital abscess is relieved preferably by endonasal endoscopic surgery – an external access may be in need only in a lateral abscess.

There is no imperative need to treat subperiostal abscesses surgically, they can also completely heal without impairment with conservative therapy (intravenous antibiosis) [575], [577], [578], [579]. Important criteria for decision are: age of the patients, size of the abscess, location, ocular symptoms and findings (vision, intraocular pressure, eye muscle function).

An age below 6–9 years, abscess volume <0.5–1.25 ml, extension of the abscess in longitudinal direction <17 mm, abscess width of <4–10 mm, medial location as well as normal vision (vision, color vision, afferent pupil reflex) are criteria that indicate positive response to drug therapy [19], [574], [575], [578], [580], [581], [582], [583], [584], [585], [586], [587], [588]. The calculation of the volume can be made according to the formula 4/3π x height x width x length [583].

In case of primary conservative therapy of a subperiostal abscess, short-term clinical control examinations are necessary because the further course cannot be predicted [581]. There are no clearly defined criteria regarding time intervals. Control examinations performed initially every hour, later every 2 (4) hours seem to be reasonable in order to be able to react immediately in cases of impaired vision. The control of color vision is helpful because an impaired color vision, especially of the red color, may be an alarming signal for upcoming impaired vision [83], [589]. Lying and sleeping with upright head of the bed is recommended because of the generally poor lymphatic drainage of the orbit [581].

The primarily conservative approach requires readiness of the surgical expertise for possible emergency intervention in case of acute deterioration. The operations itself must be expected to be difficult due to the narrow anatomical conditions in children and a bloody operative field because of a severe acute inflammation so that surgery should possibly be performed under favorable conditions and – unless there are compelling reasons to do otherwise (e.g. acute blindness) – during regular office hours. The reported recurrence rate of subperiostal abscesses of 25% in cases of transnasal and in 14% in case of external surgery in a series of emergency interventions by not very experienced ENT surgeons (fellows) may serve as a validation of the mentioned rules [590].

The necessary extent of surgery depends on the extension of the inflammatory disease and the abscess. Endoscopic surgery is preferred [19], [581]. A sufficient drainage of the paranasal sinuses involved in the acute rhinosinusitis should be achieved as well as the removal of the lamina papyracea that is necessary for sufficient abscess drainage [591], [592]. In individual cases a more advanced resection of the lamina papyracea may be necessary and the endonasal insertion of a silicone tube for better drainage of a superior or lateral abscess may be useful [593]. Even medially located abscesses of the orbital roof can be treated endonasally. The periorbit may remain intact in these cases.

Unless the abscess cavity can be visualized and controlled safely and completely, a bidirectional procedures with careful pressure from exterior on the bulb can be helpful while a blunt instrument exposes the abscess cavity under endonasal endoscopic control [594]. This technique is also recommended in intraorbital abscesses [594].

Abscesses that cannot be treated safely with an endonasal approach, in particular (supero) lateral ones [570], [584] require (additional) drainage by an external route [581], [589], [595].

Some authors advocate an external (single) abscess drainage as being useful in small children to avoid unfavorable healing results because of poor conditions of endonasal surgery (narrow anatomical conditions and severe bleeding in acute inflammation and finally increased scarring) [581], [596].

Rather often, orbital and endocranial complications occur simultaneously in the same patient [587], [588], [597], [598].

Indications for CT diagnostics (with contrast agent) or MRI are the following [53], [62], [581], [596], [599]:

- Deterioration or missing improvement within 24 hours
- Patients whose vision cannot be exactly determined
- Patients with exophthalmos, impaired eye movement, diplopia, loss of vision
- Patients with signs of intracranial complication (MRT is the method of choice).

A tendency of preferring MRI following technical improvements of the systems and increased availability can be expected.

Recurrent orbital cellulitis may serve as a clinical hint for an anatomical variation with disturbed drainage in the middle meatus that should be corrected surgically [600]. About 10% of the children with orbital complications have been treated surgically during the following 2 years because of persisting CRS (8 of 9 patients) or because of recurrence of a subperiostal abscess (1 of 9 patients). The rate did not depend on a primarily successful antibiotic or surgical therapy [601].

#### 4.1.2 Infections of the cranial bone, local spread of infection

Pott’s Puffy tumor is characterized by a frontal subperiostal abscess with concomitant osteomyelitis in (acute) frontal sinusitis [589]. It is still not clear if it is a true osteomyelitis or only a small inflammatory perforation.

Beside the intravenous antibiotic therapy, surgical treatment is indicated. Whereas formerly an external osteoplastic surgery was performed [426], [602], [603], [604], [605], there are nowadays more and more case reports on successful therapy with merely endonasal endoscopic frontal sinus surgeries, if needed with transcutaneous puncture [602], [603], [604], [605]. Concomitant or basic chronic rhinosinusitis and obstruction of the frontal sinus drainage should be treated (simultaneously) endoscopically.

Attention should be paid to intracranial complications that occur rather frequently [589], [606].

#### 4.1.3 Intracranial complications

Intracranial complications of acute rhinosinusitis preferably occur in young men/adolescents [607], [608], [609]. Most frequently those complications are subdural empyema (33%), cranial abscess (27%), meningitis (24%), epidural abscess (21%), cerebritis, or cavernous sinus thrombosis [607]. Even today, a mortality of 0–19% or long-term morbidity of 8–33% must be expected [19].

The individual therapy scheme is established and coordinated on an interdisciplinary basis and consists at least of an intravenous antibiotic therapy and in most cases of an neurosurgical abscess drainage.

The question if, when, and which surgery of the paranasal sinuses should be performed is not definitely clarified [19], [608]. The statement that the simultaneous surgical therapy of the paranasal sinuses and the intracranial disease is a milestone in the therapy of the intracranial complication of acute rhinosinusitis [607], is not confirmed with certainty by the literature [598]. In this retrospective study, 3 of 9 patients required revision craniotomy after initial craniotomy without sinus surgery in comparison to 5 of 26 patients after initial craniotomy with sinus surgery [598]. Some authors recommend an early simultaneous and more aggressive surgical procedure [597], [610]. However, they refer to non-controlled case series. Other authors recommend more reluctant indication and limited intervention [608], [609]. An indication is given when the paranasal sinus causing the complication is directly connected with the intracranial collection of fluid, in recurrences of intracranial complication after neurosurgical drainage or persisting CRS after healing of the intracranial complication [598], [608].

In summary, the present literature does not allow justifying a routine indication of emergency sinus surgery. In contrast it must be verified in every case if individual factors (e.g. anatomical abnormalities, skull base defects, underlying CRSwNP, osteoma that obstructs drainage) exist that seem to make endonasal endoscopic sinus surgery appropriate.

It is necessary to check if a skull base defect or a dural lesion might have caused an ascending intracranial infection. This aspect has been analyzed and reported most frequently for meningitis [611], but in general it is applicable for each form of intracranial infection.

**Figure 10:**
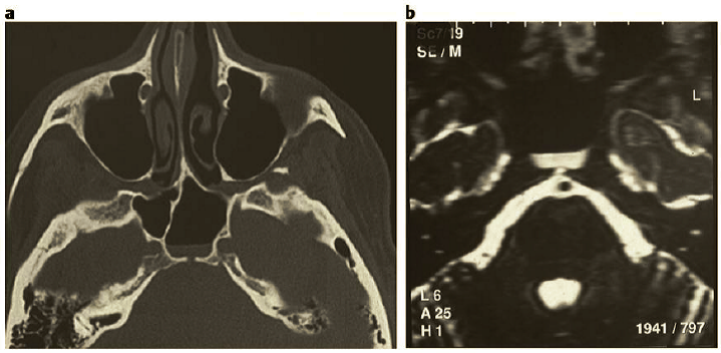
Female patient with condition after pneumococci meningitis and bony defect of the posterior wall of the sphenoid sinus (CT scan) as well as liquor passage into the sphenoid sinus (MRI). a) axial CT scan. b) MRI (CISS sequence).

**Figure 11:**
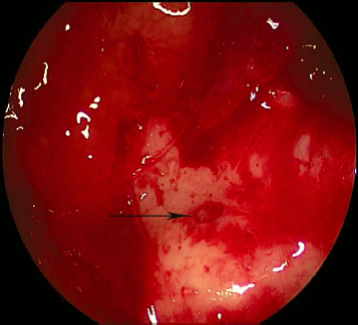
Female patient with pneumococci meningitis and acute sphenoid sinusitis and intact skull base in thin layer CT scan. Intraoperatively, a bony defect measuring 1–2 mm is obvious at the posterior wall of the sphenoid sinus/anterior wall of the pituitary.

Meningitis caused by pneumococci or *Hemophilus influenza* or recurrent bacterial meningitis is often based on a defect of the skull base and the dura unless there is immunodeficiency [611], [612], [613]. This defect may have developed by trauma (not always remembered), previous surgery, or spontaneously or it may be an occult deformity [614], [615], [616]. For diagnosis, a high-resolution CT scan of the skull base is indicated, possibly also MRI with CISS sequences (Figure 10 (Fig. 10), [617]). However, despite the presence of a defect, those scans may be inconspicuous (Figure 11 (Fig. 11)). Additionally, fluorescein application is recommended in suspicious cases or – after weighing up benefit and risks – fluorescein application and endoscopic depiction of the skull base may be performed to securely exclude a defect.

In any case, confirmation of a skull base lesion, dura defect, or CSF leak requires duraplasty!

In case of cavernous sinus thrombosis, a combined therapy of endonasal (sphenoid) sinus surgery, intravenous antibiotic therapy, application of cortisone, and anticoagulation is recommended [19].

#### 4.1.4 Persisting complete opacification of frontal, sphenoid, posterior ethmoid sinuses

Up to now there are no definite rules regarding the procedure if complete opacification of the frontal, sphenoid, or posterior ethmoid sinus appear in the imaging examination performed because of other indications. Valuable data regarding the spontaneous course are not reported in the literature. Control MRI seems to be reasonable in symptom-free patients after 3 months. The application of a cortisone spray can be discussed. During this time, a temporary, inflammation-related lesion should regress. In case of persisting findings in the sense of fluid collection, surgery seems to be indicated to prevent possible inflammatory complications.

### 4.2 Recurrent acute rhinosinusitis (RARS)

The development and incidence of viral upper airway infections (“common cold”, “rhinitis”) cannot be influenced by sinus surgery. Those upper airway infections are mostly associated with concomitant sinusitis of which the symptoms are not in the focus.

An indication of sinus surgery is given when the concomitant sinusitis causes relevant symptoms dominating the disease and thus leading to an advanced total morbidity due to

- Frequent occurrence (>4 times per year; [618], [619]),
- Very relevant symptoms and/or
- Long duration of the disease in the individual case.

A narrow ethmoid, the presence of infraorbital cells [203], or allergic rhinitis [620] favor the development of RARS.

The aim of a surgical intervention is the removal of drainage obstacles in the affected paranasal sinus segment with preservation of marginal mucosa that is inconspicuous in the interval.

The effectiveness of endonasal endoscopic sinus surgery in the therapy of RARS is proven: the number of lost work days due to illness and medical consultations and the intake of antibiotics is significantly reduced. The incidence of acute inflammation episodes is reduced to less than the half [621], [622], [623], [624], [625], [626], [627], [628].

### 4.3 Aerosinusitis, barosinusitis

Also recurrent aero- and barosinusitis with typical headaches or facial pains mostly occurring in flight descent can be avoided or healed most probably by endonasal endoscopic sinus surgery (84–100%) [629], [630], [631], [632], [633]. The postoperative ability to work of flying personnel remains long-term [629].

It is recommended that the surgical approach is adapted to the clinical complaints [629]. Only the affected paranasal sinuses should undergo surgery, e.g. as partial uncinectomy with moderate enlargement of the maxillary sinus fenestration in case of involvement of the maxillary sinus or as frontal sinus drainage type IIa in case of frontal sinus involvement.

### 4.4 Choanal atresia

Endonasal endoscopic surgery of choanal atresia is established as successful therapy. Even in preterm infants with very low body weight it allows precise performance of surgery with exact visualization, which facilitates the complete removal of the atresia and the development and transposition of mucosal flaps.

Due to the multitude of open questions, a recent Cochrane review could not draw the conclusion of a final assessment [634]. On the one hand the open questions could not be definitely answered because of the rareness of the disease and the complexity of the manifestations, on the other hand, numerous aspects reported in literature turned out to be antiquated due to improved technical standards.

The enormous number of case series published in the literature describes retrospectively different techniques with different application in children of different ages with uni- or bilateral atresia. The mixture of first and revision interventions for bony, membranous or combined atresia makes the evaluation difficult as well [635].

Important questions are: Which extent of resection is necessary to achieve permanent success? Are there postoperative options that lead to a better outcome? Is the application of Mitomycin C useful or not? If yes, which dosage and duration of the application? Are there long-term side effects that are currently not known? Are stents helpful? If yes, which type of stent and for how long? If yes, does its benefit justify a possibly increased morbidity?

Despite the multitude of present publications, these and other questions are not definitely answered. Thus it seems to be reasonable to choose therapeutic strategies that at least are in the frame of usual success rates and that lead to a minimization of short- or long-term morbidity.

The following aspects of therapeutic strategy seem to be sufficiently proven and useful regarding therapy of choanal atresia:

The intervention should only be performed by a surgeon who is very experienced in endonasal surgery.

Even in cases of bilateral choanal atresia, a routinely performed emergency intervention “at night” or “at the weekend” is neither necessary nor reasonable. There are reports about bilateral choanal atresia in children and also in adults [636], [637], [638], [639]. 

Generally, postoperative scarring leading to reduction in size of the new choana occurs in an individual extent. The specific collagen remodeling process has to be considered as we we have usually slightly concave wound surfaces.

Favorable preconditions for minimization of scarring are generally:

- Minimized operative trauma with creation of possibly small wound surfaces requiring epithelization so that cutting or punching instruments are appropriate.
- Creation of opposed mucosal flaps to cover critical wound surfaces [640], [641], [642], [643], [644], [645], [646].
- The application of slightly curved shaver drills for removal of bone in addition to bone punches has to be preferred to the classical drilling system because of a better view and avoidance of an overheated surgical site.
- Resection of the atresia plate until the anatomical limits of the nasal floor, the lateral nasal wall, and the roof of the choanae or the caudal part of the anterior wall of the sphenoid sinus are reached.
- Resection of the posterior part of the septum to create a larger three-dimensional passage into the nasopharynx because the width of the posterior nasal area on the side of the choanal atresia is significantly reduced in comparison to the healthy side. The lower edge of the middle turbinate serves as landmark for the upper resection limit [645], [647].

Because of the differences regarding the patient populations, the surgical techniques, and the study methods, the recurrence rates amount to 7–42% for unilateral and 8–65% for bilateral choanal atresia [635], [648], [649], [650], [651], [652]. A historical literature review up to 1985 revealed rates of 40–80% [653].

The application of flap techniques for coverage of wound surfaces, the resection of the posterior part of the septum and the avoidance of stents in a total of 76 patients led to a success rate of >90% applying a single intervention (47/50 unilateral, 23/26 bilateral) [640], [642], [644], [646], [647], [654]. The use of mucosal flaps that are stabilized with a Teflon quill led to a positive outcome in all 18 cases even without resection of the posterior septum [641].

A meta-analysis with 238 cases performed in 2008 came to the conclusion that independently from the applied method (simple perforation, complete excision of the atresia, mucosal flap, stent) a success rate of 85% prevails. Only previous surgery is suspected to be a negative risk factor [655]. One case of death was described because of postoperative bleeding (0.4%) and low-grade complications in 14.2% of the cases (mucosal bleeding, granulations, small synechiae, septal perforation, nasal crusting).

The meta-analysis did not include all publications with application of mucosal flaps. Moreover, any analysis should consider the insertion of stents in addition to mentioning the application of local mucsal flaps due to their specific effects on the outcome. Without stents, there is a revision rate of 6.4% (see above), with stents it amounts to 13.7% [655].

The very high success rate of 96% remains an outstanding result that was achieved with exclusive puncture, dilatation and stenting of bilateral choanal atresia [656]. The positive effect may at least partly be attributed to the stent remaining for three months. By means of a combination of mucosal flaps and short-term stenting of 5–7 days, a very good success rate of 96.9% and 86.4%, respectively, was achieved in a big case series (32 unilateral and 22 bilateral cases) [657].

Balloon dilatation was applied in addition to resection of the atresia and the insertion of stents [658], [659], [660]. In 2 studies, the balloon was used during resection of the atresia plate and the short-term insertion of a stent – the success rate, however, was only 0% for initial surgery (N=11, [659], [660]). To achieve sufficiently wide choanae, 3.8 and 3.6 procedures were necessary on average.

In the majority of the reports, the application of Mitomycin C seems to bring no benefit [635], [649], [650], [661]. Other authors observed less granulations, recurrent stenoses, and revision surgeries, in comparison, however, to patients treated with stenting [652].

The majority of single publications and review articles do not see a benefit in the insertion of stents [648], [649], [650], [652], [661], [662], [663], [664], [665]. In contrast, there are even new disadvantages. Granulations, early dropping out and dislocation, crusting, skin lesions at the nasal entrance are well-known risks [664], [666]. Side effects of antibiotics that are usually applied during the time the stent is inside [667] and the problem of repeatedly necessary anesthesia to remove and change the stents are additional factors. Generally the effect and effectiveness of the stent depend on the duration of the application [464]. Its effectiveness regarding an avoidance of scarring stenosis increases with the duration of insertion [666].

A significant risk factor for restenosis is a very young age, which is automatically associated with bilateral choanal atresia and narrow anatomical conditions and possibly also with additional deformities [649], [651], [652], [661]. The learning curve of the surgeons seems to be an additional relevant factor [651]. 

Following recent improvements in the postoperative treatment after endoscopic sinus surgery (see chapter on postoperative care) the below-mentioned procedures seem to be appropriate and justified without relevant strain:

- Endoscopic surgery is characterized by minimally bleeding if it is performed carefully. If needed, persisting bleedings are intraoperatively coagulated so that nasal packing for hemostasis can be avoided.
- Nasal packing can be useful because it avoids the development of clots in the surgery site which may lead to granulations and scarring. Thus, nasal packing could be justified for (half) a day.
- Occlusion of the nose, if tolerated, reduces crusting that leads to nasal obstruction and to dislocation of the mucosal flap when it is removed.
- In combination of occlusion with nasal rinsing by the parents, the nose is cleaned and moistened and the conditions for rapid spontaneous epithelization are optimized.
- Follow-up concepts described in the literature with one or more second look cleanings under general anesthesia seem to be too invasive with regard to known harmful effects of anesthesia to the pediatric brain in newborns and infants.
- Endoscopy and precise instrumental cleaning on the examination chair is nearly impossible in babies or infants.
- For routine antibiotic therapy beyond mere perioperative prophylaxis, there are no sufficient reasons [655] even if often longer-lasting antibiosis is performed.
- Local application of cortisone with intranasal drops for one week seem to be reasonable to reduce the tendency of granulation and it is sufficiently safe regarding undesired side effects.

In summary, there are convincing data to advise to endoscopic surgery with creation of counter-rotating mucosal flaps, resection of the posterior nasal septum (1 cm to one third), and avoiding of stents. For postoperative care, occlusion is recommended if tolerated, and also intensive nasal rinsing as well as short-term application of topical steroids.

The general reflections to avoid anesthesia with its immanent risks are currently discussed and new knowledge is gained:

Anesthesia in pediatric patients can negatively influence the cognitive and behavioral development (meta-analysis: [668]. Especially repeated anesthesia should be avoided (meta-analysis; hazard ratio 1.75, CI 1.31–2.33: [669]). In contrast, a position paper of the working group on pediatric anesthesia and neuro-anesthesia of the German Society of Anesthesiology and Intensive Care Medicine formulates:

“There are nearly no hints that competently performed and clinically well supervised anesthesia with modern shortly effective anesthetics was associated with negative consequences such as cognitive retardation or learning disability” [670], [671].

#### 4.4.1 Choanal polyp

Antrochoanal polyps (ACP) are benign lesions that develop from the mucosa of the maxillary sinus, grow through the natural or secondary ostium into the nasal cavity, reach the choanae, and leads in particular to nasal obstruction. More rarely, choanal polyps may originate in the sphenoid or ethmoid sinuses.

Therapy of choice is the surgical removal including the base of the polyps. The simple abrasion is associated with a high recurrence rate. After removal of the intranasal part, performance of uncinectomy and enlargement of the natural maxillary sinus ostium, the base of the polyp has to be located. This requires the application of angular optics and special angled instruments with which often the anterior wall, the palatine, alveolar, or prelacrimal recesses are not seen or reached [291] Not rarely, this leads to residual ACP parts or hidden second or third cysts that may be the origin of recurrences [672].

If the secure visualization and removal of the base of the ACP is not possible via endoscopy of the middle meatus, a complementary approach via the canine fossa [673], a prelacrimal or postlacrimal approach [293] must be considered. A usual approach via the inferior nasal meatus often does not provide sufficient overview of the whole maxillary sinus. This concerns mainly ACP that have a broad base or develop from several points, or cases with inflammatory component that make differentiation between thickened mucosa and the actual ACP difficult [672], [673].

The recurrence rates amount to totally 0–20% [674], [675], [676], [677], [678], [679], [680], [681], [682], [683], [684], [685]. Despite certain methodological objections regarding a comparison, the recurrence rates in cases of additional procedure via the canine fossa are constantly lower (0–8%) than in procedures exclusively via via the middle meatus.

If after primary endonasal endoscopic complete removal of ACP it is not definitely obvious despite extended middle meatal antrostomy grade 4, there are two remaining options:

- Terminating the intervention with an a-priori uncertainty regarding the complete removal and acceptance of an increased risk of recurrence
- Additional access via the canine fossa or prelacrimal approach. Possible complications are a lesion of branches of the infraorbital nerve, of the teeth, or of the growth areas of the maxilla in children, or of the nasolacrimal duct.

The according procedure should have been part of patient’s informed consent preoperatively.

In rare cases, the choanal polyp originates from the sphenoid sinus which requires the removal of the polypous base in the area of the sphenoid sinus ostium or in the sphenoid sinus in analogy to the antrochoanal polyp [686], [687].

#### 4.4.2 Dentogenic maxillary sinusitis

Reasons for dentogenic maxillary sinusitis are often preapical and periodontal abscesses based on caries or periodontal diseases and foreign bodies due to dental measures (dental roots, dental filling material, instruments, dental implantations, sinus augmentation), partly associated with oroantral fistulas. Most frequently, the molars are affected, followed by the premolars [688].

The incidence of dentogenic sinusitis seems to increase during the last years [689]. Especially with advanced involvement of the maxillary sinus and unilateral disease, a dental genesis must be considered [690] as well as in cases of unilateral disease combined with putrid smell [680]. The ENT specialist must be aware of the fact that a dental origin is often overlooked by radiologists as well as by dentists so that a so-called inconspicuous dental or radiological examination cannot exclude dental genesis [691], [692], [693]. 

In the context of diagnostics, an ENT specific and dental examination including radiological assessment by means of CT scan/CBT [691] are required.

Regarding the sequence of treatment, first drug therapy (antibiotics) should be applied considering the circumstance that anaerobic bacteria are also included [694]. Parallel, a dental therapy should be performed. Persisting pains (mostly purulent secretion, putrid smell, pains) and pathological findings (purulent secretion, obstruction of the osteomeatal unit) should lead to sinus surgery [695], [696]. In cases of foreign body impaction, there is always the indication to removal. Because of the high success rate with simultaneous low complication rate, endonasal endoscopic surgery is the therapy of first choice, followed by transoral surgery according to the Caldwell-Luc technique [697]. In cases of oroantral fistulas, the combined endonasal and oral surgery may be necessary [698].

**Figure 12:**
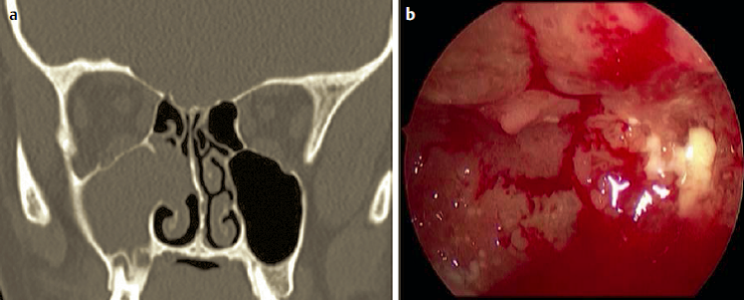
Chronic maxillary sinusitis of the right side. The causative preapical abscess was not identified neither by the dentist nor by the radiologist (CT scan). The papillomatous thickened mucosa can and should be preserved (intraoperative endoscopy, 45° optics).

Partial uncinectomy, rinsing of the maxillary sinus, and if needed (moderate) enlargement of the natural maxillary sinus ostium and removal of an infraorbital cell is sufficient in most cases of mere obstruction of the maxillary sinus drainage [699]. Thickened mucosa, partly appearing as a papilloma, can and should be preserved because after removal of the obstruction, accompanying antibiotic therapy, and appropriate dental measures, this situation improves (Figure 12 (Fig. 12)). The removal of foreign bodies or the removal of a dentogenic bony cyst require enlarged accesses.

Often opacification of the frontal sinus (43%) and ethmoid sinus (65%) is visible that is individually treated [693].

#### 4.4.3 Duraplasty

Independent from the etiology, the detection of a fronto-basal CSF fistula leads to indication of surgical closure [700]. Because of the high risk of intracranial infection (as well as its high morbidity and mortality rate) possibly rapid closure of the defect should be performed in fronto-basal lesions of the dura. I suspected cases the condition should be clarified [488], [701], [702].

The average risk to develop meningitis in case of an accident-related lesion of the dura without specific treatment is calculated in a different way: 0.1–0.2 infectious events per month [703], 0.3 events per year [704], 10% risk per year [705]. For the long-term total risk, there are date varying from 10 to 85% [704], [705], [706], [707], [708], [709], [710], [711], [712], [713], [714], [715]. Within the first year, the risk is highest [704].

In summary, there is a high risk to acquire meningitis after untreated rhino-basal lesions of the dura. Apparently it is even higher after accidents and resulting pneumatocephalus compared to spontaneous CSF with intracranial hypertension [704], [707]. Even episodes of late meningitis are possible in cases of spontaneously ceasing liquorrhea, intervals of up to 48 years are reported [616], [713] (see also chapter on indications for surgery in case of intracranial complications).

Because of the high success rate of endonasal endoscopic duraplasty with simultaneously low morbidity in comparison to the incidence and morbidity/mortality of meningitis or other intracranial infection, conservative therapy (confinement to bed, positioning with elevated bedhead, avoiding an increase of intracranial pressure, normalization of an increased intracranial pressure, if needed lumbar drainage in cases of persistence of 3–7 days) [711], [714] cannot be recommended in cases of rhino-basal lesions of the dura. An exception is a temporary rhino-liquorrhea without proved fracture if the findings are considered as being a consequence of a tearing of a filum olfactorium [700].

The preoperative diagnostics should include high-resolution computed tomography, in single cases completed by MRI (e.g. presentation of menigoceles, encephaloceles; even CSF fistulas; important parameters are T2 weighting, 3D-CISS and FLAIR sequences; [53], [62]). 

The clear intraoperative location and exposition of the defect is crucial [274], [448], [700], [715], [716], [717]. In case of recurrences, the possibility must be taken into consideration that several or newly occurred defects are present [448], [716].

Hence, for pre-/intraoperative location of the defect and intraoperative verification of the sufficiently performed duraplasty, endoscopy with application of a blue light and complementary blocking filters after previous lumbar application of fluorescein solution is recommended and indicated as safe procedure with a specificity of 100% and a sensitivity of 74–96% [165], [448], [700], [702], [718], [719], [720]. As fluorescein solution is not an officially admitted form of application (off-label use), a specific informed consent of the patient is obligatory [718]. Partly up to 50 mg fluorescein were applied [721]. Central nervous complications have been described for suboccipital application [719], [720], after optimizing several factors (dosis) no more complications occurred [720]. The only complication reported hereafter was not specified with regard to the applied quantity [722]. It seems to be reasonable to minimize the applied quantities of fluorescein. So a lower dose of max. 10 mg fluorescein is recommended where no complications and side effects are described beyond those of the procedure of lumbar puncture itself with apparently identical diagnostic accuracy [718]. The original 10% solution (1 ml) can be diluted with 10 ml liquor to 1%. It seems to be more useful to order a 1% solution to be produced in time by the pharmacy ready for use in the morning and to apply a maximum of of 1 ml of a 1% solution (0.1 ml/10 kg). A position with lowered head for 2 hours is recommended because the fluorescein solution has a higher density than liquor.

Topically applied fluorescein is reported to be similar helpful. Staining is achieved by the excaping liquor “washing out” the color locally. There are only few literature reports on that topic [723], [724].

In addition to endoscopic duraplasty, it is currently recommended to immediately vaccine patients with meningitis in cases of suspected or proven lesion of the dura against the most frequent relevant bacterial germs (*Pneumococci, Meningococci, Haemophilus influenzae*) [169]. Hereby vaccination against *Pneumococci* is the most important one and according to official statements of the German authorities (STIKO) it is officially indicated for CSF fistula (http://www.rki.de/DE/Content/Kommissionen/STIKO/Empfehlungen/Impfempfehlungen_node.html).

After any seeming uneventful sinus surgery, strong and/or persisting headaches, already on the day of surgery, are suspect with regard to lesion of the dura and intracranial complications. Even a severe discomfort, nausea, and vomiting that on one hand often occur related to anesthesia, has to be regarded as a putative signal for a lesion of the dura and intracranial complications [725]. 

Endonasal duraplasty is considered as therapy of choice to close nearly every defect of the dura of the anterior skull base [165], [700], [715], [726], [727], [728], [729], including therapy of meningoencephaloceles [730], [731]. Beside the equivalent or even superior success rates in comparison of extranasal approaches, the advantages of the transnasal approach are a reduction of the surgery-related morbidity and mortality, the preservation of olfaction, and cosmetic aspects [727], [730]. Even defects of the posterior wall of the frontal sinus can often be closed via a frontal sinus drainage type IIa or type III [732], [733], [734], [735]. Extranasal approaches are applied when the liquor fistula cannot be completely exposed endonasally or when an extranasal access is primarily applied, e.g. in oncologic surgery or traumatology [700].

Small dura defects after trauma, accidental lesion during sinus surgery, or spontaneously occurred, can be closed with a success rate of 90% at initial surgery and in up to 97% including revision surgery [165], [727], [736].

The complication rate regarding meningitis, subdural hematoma, and intracranial abscess amount to less than 1% each [727] or 0.03% [165]. The results do not depend on the applied technique of duraplasty [165], [700], [727].

In cases of large skull base defects after tumor resection, the success rate amounts to 88–92% [737], [738]. The application of vascular pedicled flaps leads to better results than using free grafting (93% vs. 84%, [737]), whereas this is true in particular for dura lesions with high liquor flow (94% vs. 82%) [738]. The results are at least comparably good than the ones after external duraplasty [730], [739].

Depending on the size, location, and origin of the defect, a simple “onlay” or “underlay” technique, combined “onlay-underlay” technique, or multi-layer procedures with different autologous (fat, mucosa, connective tissue, muscle, cartilage, bone) or allogeneic materials, free or vascularized transplantations are applied [448], [700]. An exhaustive description of the different materials and flaps is given in the complementary review on rhino-neurosurgery in this issue [4].

An important criterion for the choice of techniques and materials is the experience of the surgeon and the availability of materials [700], [727].

The larger the dura defect is, the higher the liquor flow and the more risk factors are present (e.g. previous irradiation), the more “stable” duraplasty should be. This is especially true for large defects after tumor surgery, duraplasty of the sphenoid sinus, and in cases of increased cranial pressure.

Vascularized grafts have become the method of choice in cases of large defects [718], [726], [729], [738], [740]. Multi-layer duraplasty and special techniques are appropriate for large defects and high-flow dura lesions.

- Three-layer duraplasty is performed intracranially intradurally (e.g. fatty tissue), intracranially extradurally (connective tissue), and extracranially (vascularized flaps) [700], [741], [742], [743] or only intracranially intradurally (fatty tissue and connective tissue) and extracranially [744].
- Fascial grafts can be placed on the defect in an overlapping way and fixed in a water-tight fashion by means of a sufficiently stable material (e.g. bone) put in the defect like a gasket (“gasket seal” technique [745]).
- A piece of fatty tissue measuring 1–1.5 cm may be taken from the earlobe. It may then be equipped by a vicryl suture passing through the fat and inserted into the defect. By means of the suture, the fat may be pressed into the defect (“bath plug” technique [274], [746], [747]).
- Furthermore, obliteration has been described for the sphenoid sinus [700], [715], [748].
- Duraplasty of the sphenoid sinus is particularly difficult because of the high liquor pressure, the sometimes very close location near the internal carotid artery as well as the optic nerve. Additional difficulties may be due to liquor fistulae in the lateral wall of the sphenoid sinus with relevant pneumatization or in the so-called Sternberg’s canal [749], [750]. In this context, the transpterygoid transsphenoid approach has proven to be appropriate [349], [751], [752], [753], [754].
- Concerning vascularized flaps, the following options may be mentioned: intranasal flaps, pedicled at the sphenopalatine artery, the arteries of the inferior and middle turbinates, the anterior ethmoid artery, the superior labial artery, and the artery of the incisive canal as well as regional flaps pedicled at the supraorbital/supratrochlear artery, the superficial temporal artery, the facial artery, and the major palatine artery [729], [737], [755], [756], [757] (see also the review on rhino-neurosurgery in this issue). Complications with long-lasting crusting, nasal secretion, and reduced or lost olfaction are frequent side-effects of the application of nasoseptal and other vascularized flaps [737]. For reduction of the morbidity, the septal mucosa of the opposite side can be placed from dorsal to the donor side after usually necessary resection of the posterior half of the septum (“reverse flap”) [274], [758], [759].
- The “onlay” technique is recommended when the “underlay” technique bears a high risk of injury of nerves or vessels.
- A new technique is the endoscope-assisted injection of fibrin glue into postoperative recurrences of dura fistulae [760].
- In case of duraplasty in the area of the frontal sinus, the anterior roof of the ethmoid sinus, or the anterior lamina cribrosa, attention must be paid that postoperatively the frontal sinus drainage is not impaired. Some authors even insert prophylactic stents [166].
- Liquor fistulae and meningoceles in the area of so-called Sternberg’s canal (lateral cranio-pharyngeal canal) are found in the lateral wall of the sphenoid sinus medial to the maxillary nerve and are the result of a missing bony fusion of the pre-basal sphenoid with the greater wing of the sphenoid [741], [750], [754], [761]. Not all described cases had the dura defect in a location medial to the maxillary. Etiologically different are lesions in the lateral recess of the sphenoid sinus lateral to the maxillary nerve [749].
- Encephaloceles are coagulated to the level of the bony skull base defect and resected [166], [720].

A routinely performed lumbar drainage is not indicated taking into account the specific increase of system-related complication rates. In cases of postoperative CSF leak, however, it seems to be reasonable to insert lumbar drainage which will contribute to spontaneous healing in 40%. Persistence of CSF leaks irrespective to lumbar drains are in need of surgical revision. Evidence-based protocols are not published [165], [738], [762], [763]. Is the liquor pressure is higher, the indication is made more generously. An increased pressure can also be reduced by application of acetazolamide [764].

If autologous mucosal transplantations are performed, a shrinking of about 20% must be taken into account. The transplantation should be larger than the defect by one quarter or 4 mm [448], [765].

Different materials show different healing properties. Mucosal transplantations and collagen matrices heal more quickly and with less crusting than acellular dermis [766]. Even in vitro it could be revealed that collagen materials are very well epithelized in contrast to cartilage or poly-p-dioxanone [767]. This fact has to be considered when using cartilage for covering larger defects [768], [769].

Crusts can impair a rapid and unhindered epithelization and cause nasal obstruction. The necessary local postoperative care is unpleasant for the patient and bears the risk of removing or dislocating the onlay dura transplantation [766].

For stabilization of a duraplasty, gelatine, cellulose, and different types of packings are used among others [448], [700]. Besides stabilization of the transplantation, the insertion of oxygenized cellulose also achieves an induction of granulation tissue that supports the successful closure of a fronto-basal liquor fistula. Regular or absorbable nasal packings are used according to the individual preference of the surgeon in 60–86% [464], [721], [727] and remain often for 3–5 days [727]. In the context of rhino-neurosurgery, regularly balloon catheters may be inserted for stabilization of the duraplasty for 10–14 days [744], [770].

Investigations on the necessity and effectiveness of nasal packing are not present. A sufficient stability of the non-vascularized duraplasty materials by beginning new vascular ingrowth and incorporation by connective tissue seems to be achieved after around one week, comparable to the physiology of general wound healing [765].

While antibiotic prophylaxis is not indicated in cases of uncomplicated CSF leak/dura lesion [168], [140], the perioperative application of antibiotics is generally recommended in the context of duraplasty. It is performed as long as nasal packing or lumbar drainage are in situ and should be sufficiently effective against *Staph. aureus* [163], [165], [166]. There is no clear evidence that confirms the benefit of a long-term application of antibiotics going beyond this time [165]. Reports of uncomplicated endonasal duraplasty with application of nasal packing without antibiotics exist [167]. 

Postoperatively, the following measures are recommended depending on the type and extent of duraplasty [448] – without definite proof for their necessity [720]:

- Bed rest for one or several days
- High positioning of the head of the bed (e.g. 45°)
- Avoidance of an increase of the intracranial pressure by pressing, coughing, sneezing, heavy lifting
- Follow-up examination after duraplasty primarily occurs endoscopically. In single cases, imaging can be indicated for secure exclusion of the development of a mucocele [714]. Single authors recommend routine control with application of fluorescein [718], [748] which can be considered at least in larger or difficult to manage dural defects. In order to wait for a nearly completed wound healing, those control examinations should be performed not before 6 months after surgery [389].
- The long-term experience of a specialized center may help to establish specific algorithms regarding the appropriate choice of duraplasty technique [729].

#### 4.4.4 Epistaxis – coagulation of the sphenopalatine artery

Nosebleeds concerns more than 60% of the population at least once in lifetime [771], [772] and in 1 of 200 cases it is the reason to contact the emergency unit, with specific affection of elderly people and preference of the winter [773]. Inpatient admission occurs in 6% of the cases. Emergency visits take place in 1.7 of 1,000 patients per year.

Depending on the location, origin, and intensity of the nosebleeds, a therapy is performed by means of acid etching, electro-coagulation, insertion of nasal packing, surgical hemostasis, or embolization.

The surgical hemostasis, that is subject of analysis in this context, includes the occlusion of the sphenopalatine artery (ASP) in cases of posterior epistaxis. 

The term of “sphenopalatine artery ligation” originates from the time of transantral presentation and ligation of the maxillary artery and is no longer appropriate, however in the English literature it is still mentioned and furthermore it is used in our regular coding system.

According to an evaluation of the literature, endonasal occlusion of the sphenopalatine artery has a higher success rate in comparison to antral ligation or nasal packing [774], [775], [776], [777] or a comparable success rate with a lower complication rate [778]. Nowadays, mostly electro-coagulation (more exactly: bipolar, but also monopolar coagulation), more rarely clipping is performed. Coagulation achieves better results than clipping [779]. It is the most effective surgical therapy with the highest cost efficiency [780].

The effectiveness of both procedures, embolization and coagulation of ASP, is similar. Success rates of 85–90% are mentioned, measured in a bleeding-free interval of at least 4–6 weeks [781], [782], [783], [784]. At least in the USA, the expenses of embolization are higher [783], [784], [785], [786].

It depends on different factors which procedures is primarily applied in case of emergency: local availability of the resources, comorbidities, consumption of anticoagulants, patient’s preference, and costs. The advantages of coagulation of ASP are a lower rate of severe complications (see below), a precise location of the site of bleeding, the location of another source of bleeding than ASP (e.g. anterior ethmoid artery) with single-step treatment. Embolization can be performed under local anesthesia, allows the identification of vascular anomalies, and causes an unimportant trauma of the nasal mucosa [785].

The complication rates regarding mortality, stroke, blindness, or blood transfusion are considered as being rather low equally [783]. After embolization, low-grade complications occur in around 20% of the cases, and severe complications in 2% [781], [782], [787]. Minor complications were described as self-limiting and consist of temporo-facial pains or a sensation of numbness, headaches, swellings, pains when chewing, or trismus. Severe complications are hemiplegia, paresis of the facial nerve, skin necrosis, or blindness [781], [788], [789].

Complications after surgical occlusion of the ASP are crusting and nasal dryness (34%), numbness of the palate (13%), acute sinusitis (3%) reduced lacrimation (3%), or septal perforation (3%) [775], [790]. Intranasal hypoesthesia could be objectified in 19%, however, the patients did not complain about it [791]. After an average of 6.7 years, 10% of the patients underwent revision surgery, 23% suffered from minor complications, most frequently persisting crusts [792].

Generally different types of invasiveness are possible. The least invasive type is the transmucosal coagulation of the branches to the inferior and middle turbinates in the area of the posterior attachment of the middle turbinate as well as transnasal coagulation of the posterior nasal artery at the anterior wall of the sphenoid sinus. Uncontrolled and wide-field coagulation bears a higher risk of thermal damage of the vidian nerve, the sphenopalatine ganglion, or the branches for lacrimation, the major palatine nerve etc., for example if monopolar coagulation is performed.

The sphenopalatine artery with its branches can be identified more invasively and precisely via an incision of about 1 cm anterior to the posterior attachment of the middle turbinate in the middle meatus. After U-shaped, dorsally pedicled, or vertical incision, the mucosa of the lateral nasal wall is lifted subperiostally and the crista ethmoidalis of the palatine bone is identified, whereby a suction elevator is particularly useful. The crista ethmoidalis is a crucial landmark for the foramen sphenopalatinum [793]. If the crista ethmoidalis is removed, a significantly better exposure of the branches of the ASP is possible. A difference in the invasiveness results if the branches to the inferior and middle turbinate that usually become obvious are not only coagulated in a controlled way but also intersected in order to exactly expose and coagulate further dorsally located branches (most invasive type of intervention). The individual anatomy of the foramen sphenopalatinum with a frequent split-up of the artery into numerous (up to 10) branches [794], [795], [796] justifies the most invasive variation, that is usually reserved to revision interventions.

At the same time this means that the middle turbinate is destabilized and the wound surfaces are enlarged, which is often not desirable in patients who receive several anticoagulants. A bilateral approach in cases of unclear source of the bleeding bears the risk of necrosis of the posterior septum [797].

The standard uncinectomy, identification and enlargment of the maxillary sinus ostium [798] are not necessary. The reasons for failed coagulation of the ASP may be: re-opening of closed vessels, bleeding from branches of the sphenopalatine artery that are not (yet) adressed, bleeding from branches of the anterior ethmoid artery (AEA) or (which is extremely rare) from the internal carotid artery [780].

The AEA can easily be identified and coagulated in the context of paranasal sinus surgery [799], [800]. Peripheral branches (anterior superior, lateral, or medial), however, can easily be overlooked during endoscopic hemostasis in cases of epistaxis because they are already passed when the 0° endoscope is inserted into the nose and can no longer be found if current bleeding is missing. They have to be identified in a controlled way.

Acute massive bleeding should be treated rapidly by sufficient posterior nasal packing [780]. An early final intervention by coagulation of the ASP or embolization is recommended [786].

#### 4.4.5 Focus?

Often the question of existence (releganve) and therapy of a “sinogenic focus” is asked. Hereby, acute sinusitis is in the focus. As chronic rhinosinusitis is usually no primary bacterial disease, the idea of focus in immunocompetent patients with chronic rhinosinusitis may be discussed only reluctantly [801]. Data quality and knowledge based on the literature is very poor. Generally, there are single cases of immunocompetent children and adults where bacteremia is diagnosed due to acute sinusitis. Sinogenic sepsis in immunocompetent patients is a very rare event besides cases with artificial respiration. In immunosuppressed patients the possibility of sinogenic fungal infection must be taken into consideration [801].

A rational approach is to perform conservative therapy if patients have complaints (!) and (!) an endoscopic examination has been performed revealing corresponding (purulent nasal secretion). If the complaints and the findings persist, it could be useful to perform CT scan and to perform surgery of the paranasal sinuses improving drainage of the compartments that are affected based on the CT scan [801]. In the interdisciplinary dialogue it must be clear that conventional radiography should not (!) be done to let ENT-surgeons rule out any sinogenic focus [801].

- Psoriasis with its acropustulosis subtype of pustulosis palmaris et plantaris is understood as systemic inflammatory disease (Guideline on Therapy of Psoriasis vulgaris, Nr. 013-001). Reliable data on possible (causal) relations between psoriasis and rhinosinusitis do not exist. A single cohort study from Taiwan could show an increased risk of psoriasis in patients with CRSsNP [802]. Triggering of psoriasis exacerbations by acute infections is possible (Guideline on Therapy of Psoriasis vulgaris, Nr. 013-001). From a pragmatic clinical point of view it seems to be reasonable to think about surgical procedure if patients report about recurrent or regular psoriasis exacerbations induced by acute rhinosinusitis. It may be a matter of discussion, if and how surgery can improve this situation. For example, uncinectomy and if needed enlargement of the natural maxillary sinus ostium of the affected side may be a useful and sufficient procedure in cases, where protracted pain is reported in context of common upper airway infections with longer-lasting purulent secretion from the affected maxillary sinus leading to subsequent exacerbations of the psoriasis disease.
- In cases of chronic urticaria, possible associations with rhinosinusitis are even more vague. Convincing hints for the relevance of sinogenic factors are not given in literature.
- With regard to the relevance of foci for people scheduled for heart surgery, acute sinusitis should be looked for. Chronic rhinosinusitis does not seem to be valued as focus [801]. “Wiping out” of chronic rhinosinusitis before heart surgery is usually neither necessary nor possible.
- Bland maxillary sinus cysts do neither represent chronic rhinosinusitis nor a focus that would require intervention for example before heart surgery.
- In contrast, uveitis may develop based on acute sinusitis.

In summary, according to current knowledge, specific ENT-examinations evaluating the need for endoscopic sinus surgery for improvement of distant other diseases may only be helpful in certain rare cases. ENT-Interventions may be discussed, if an obvious correlated occurrence of acute bacterial rhinosinusitis and an exacerbation of another disease is observed, provided conservative therapy was not successful or is not considered.

Patients subjected to artifical respiraton in intensive care units frequently develop nosocomial sinusitis [803], [804], [805], [806]. It can be the origin of unclear fever or sepsis ([807]: in 16% it is the only origin, in 30% partially). Risk factors are nasotracheal intubation, nasogastric tube, nasal packing, sedation, and a low Glasgow coma scale value [804], [808].

If other origins cannot be found and endoscopically visible purulent nasal secretion is revealed, CT scan of the paranasal sinuses is indicated. Positive findings in favor of the diagnosis “sinugenic fever” in these patients may be: complete opacification of the maxillary sinus or an air-fluid level [804]. Some studies report a good correlation between the detection of germs from the middle meatus and maxillary sinus puncture [809], other do not agree [810]. Purulent nasal secretion in the middle meatus, however, is a good predictor for the presence of acute sinusitis [811].

Positive radiological findings in intensive care patients with fever do not necessarily mean that a suspected sinusitis is the reason for fever [812]. Negative endoscopy has as specificity of 86% that negative maxillary sinus rinsing will be found [812].

As appropriate therapy, the removal of ipsilateral located respiration tube and a smear-based regimen of antibiotics are considered [804]. On the other hand, it seems to be reasonable to generously indicate a minimally invasive surgical intervention in doubtful cases and to perform in appropriate cases as the primary measure, because a rapid effect may be achieved with simultaneously positive influence on the pulmonary situation [805]. The partial resection of the uncinate recess and a enlargement of the maxillary sinus ostium with suction and rinsing of the purulent secretion is possible as a “bedside procedure” and does not obligatorily require transportation of the patient to the operating theater [801]. Specific, evidence-based recommendations for patients with obvious paranasal sinus pathologies being “on the list” for organ transplantations are not given in literature [801]. Currently the value of surgical therapy cannot be exactly determined. According to the present literature, conservative treatment of a diagnosed sinusitis before transplantation seems to be an appropriate way.

Also, evidence is low for patients suffering from cystic fibrosis, whether sinus surgery helps avoiding the re-colonization and infection of the lungs in patients with cystic fibrosis and lung transplantation and positively influences the survival rate. While one publication reports a negative result [813], an improved outcome could be shown when additional intensified local therapy was performed [814].

#### 4.4.6 Cysts of the maxillary sinuses

Isolated cysts of the maxillary sinuses do not represent an indication for surgery unless they cause specific symptoms. This is also true in the context of septal surgery or surgery of the inferior turbinates. In 97% of the cases, isolated cysts are free of symptoms in the further course. In case of incidental diagnosis, even control examinations are not necessary [815].

#### 4.4.7 Mucoceles

Today transnasal endoscopic marsupialization is the therapy of choice for paranasal sinus mucoceles [816], [817]. Advances in endoscopic sinus surgery significantly reduce the necessity of external surgery of maxillary and frontal sinus mucoceles [307], [818], [819] as well as the long-term insertion of stents [307], [820].

A systematic review of the literature and meta-analysis shows that frontal and frontoethmoidal mucoceles are treated more and more by means of endonasal endoscopic surgery [821]. With obviously comparable rates of recurrences and severe complications, the rate of low-grade complications is lower in endonasal procedures [821]. The recurrence rate amounts to totally <5% (0–9%) [821], [822], [823]. In another publication differing from those data and reporting a relevantly higher recurrence rate of 23%, the high percentage of patients with CRSwNP should be noticed. Furthermore, surgery of frontal or frontoethmoidal mucoceles did not include frontal sinus drainage type III but a not clearly defined endonasal opening of the frontal sinus was performed with insertion of a silicone drainage in case of a narrow anatomy [824]. By means of frontal sinus drainage type III, even difficult constellations revealing osteoneogenesis or medial prolapse of the orbit soft tissue can be controlled [242], [825]. Unfavorable anatomy, lateral location or relevant scars and osteoneogenesis may even today be indications for an external or combined approach in cases of frontal sinus mucoceles [817], [821], [822]. Intracranial growth is rare and may require an additional external procedure depending on the extension and a possible dura lesion [826].

Maxillary sinus mucoceles occur more rarely because Caldwell-Luc surgery, which is a frequent cause of postoperative maxillary sinus mucoceles, is only rarely performed nowadays. In case of regular anatomy, marsupialization should be performed without any problem via the middle meatus and be preferred to an approach via the inferior meatus [827].

An anterior or relevantly lateral location of maxillary sinus mucoceles, a thick membrane of the mucocele or thick bones in the access require alternative surgical strategies. The long-term insertion of stents in laterally located mucoceles [307] is no longer considered as being appropriate. Navigation control can facilitate the rapid and safe identification also of small mucoceles. Curved drilling systems allow acomplete bony marsupialization. The creation of medially pedicled mucosal flaps can avoid recurrences based on uncontrolled scarring [828]. Protection of the mucosa is also possible when drilling devices are used.

The pre-lacrimal approach allows consequent exploration of the whole maxillary sinus from anterior and opening and marsupialization of nearly every mucocele.

Sphenoid sinus mucoceles represent only around 2% of paranasal sinus mucoceles [829], [830], but because of the incidence of ocular symptoms (up to 85% [829]) and the risk of blindness they are regarded as emergency cases. Neurological failures of the cranial nerves III, IV, and VI (diplopia) are more often regressive than visual loss [831].

A recovery of vision is generally more probably if the visual loss has developed slowly, was not long-lasting and less severe [831], [832], [833]. Even if the visual nerve has a potential of regeneration, prediction of the individual outcome is not possible [834]. Thus, acute visual loss in cases of mucoceles of the paranasal sinuses, especially of the sphenoid sinus and the posterior ethmoid, should always lead to emergency endonasal endoscopic surgery with marsupialization of the mucocele.

#### 4.4.8 Sinus surgery in children

It is particularly difficult to answer the question of adequate therapy of pediatric chronic rhinosinusitis because the symptoms overlap enormously with other frequent diseases (allergic rhinitis, adenoiditis/increased adenoids, upper airway infections) so that clear differentiation is not possible [19]. The quality of life is significantly impaired [835].

In most cases, a surgical approach in pediatric patients is only justified after intensive drug therapy. It is a problem that the present evidence of the effectiveness of drugs is limited and the so-called “maximum drug therapy” even in pediatric CRS is not clearly defined [19], [836], [837].

Topical nasal steroids and accompanying nasal rinsing with saline solution are considered as therapy of first choice in pediatric CRS [19]. A short-term course of oral antibiotics, e.g. for several weeks, is considered as not being sufficiently evidence-based due to the EPOS paper, neither is the intravenous application [19]. In contrast, the recommendation of possibly smear-based antibiotic therapy for 3–6 weeks to avoid surgery is given in many other publications [836], [837], [838], [839], [840], [841], [842] as well as the accordingly applied practice [841]. Combining those different evaluations, it seems to be reasonable and necessary to confirm the presence of relevant purulent secretion and proof the individual infection by smears before starting antibiotic therapy.

Accompanying important diseases such as allergic rhinitis, gastro-esophageal reflux or immunodeficiency should be treated accordingly with drugs [19], [836].

For the therapeutic decision it is important to observe the predominant symptom: muco-purulent nasal secretion (permanently or recurrently), nasal obstruction, headaches, or impaired olfaction.

The evaluation of the surgical therapy in pediatric chronic rhinosinusitis is difficult to and needs differentiation.

According to several systematic analyses, the endoscopic sinus surgery in pediatric patients is generally considered as being safe and effective [559], [842], [843]:

- The complication rate is low (0.6% of severe complications; 1.4–2.0% of low-grade complications [559], [842], [843]).
- The success rate (improvement of the symptoms, improved quality of life) remains constantly high over a longer period of time with 71–100% (88%; [559], [842], [843]).
- Functional endoscopic sinus surgery can improve a disturbed mucociliary clearance [844].
- Recurrence rate amount to 13% [845], while most frequently scarring in the middle meatus and the maxillary sinus ostium were found.

Included studies and investigations, however, usually refer a very heterogeneous patient population and different surgical approaches, partly with second-look interventions.

Hence, on one hand there is no clear evidence from prospective comparative studies when and to what extent and based on what kind of complaints surgery should be performed [836], [846].

On the other hand, there is broad consensus in literature regarding the following general rules of endonasal endoscopic sinus surgery in pediatric patients [19], [836], [837], [847]:

- After failed drug therapy of CRSsNP, first adenoidectomy is performed in order to remove possible biofilms. Bacterial findings from adenoid tissue clearly correlate with the ones from the middle meatus of the paranasal sinuses [838], [848] – however, not always [849]. Children with CRS have significantly more often biofilms in their adenoids than for example children with sleep apnea [850]. The success rate of exclusive adenoidectomy amounts to 50–70% [19], [851]. The additional, optionally recommended balloon dilatation of the maxillary sinus ostium combined with rinsing the sinus [852], [853], [854] must be considered critically. The author stated financial disclosures to the company distributing the balloons. At the same time there are general objections regarding balloon dilatation of the maxillary sinus (see chapter on balloon dilatation). Bronchial asthma, Lund-Mackay score of ≥5 and ages <7 seem to be indicators for poorer success rates of exclusive adenoidectomy and may serve as a positive argument for necessary interventions of the paranasal sinuses or revisions [845], [855], [856]. As the first step and depending on the individual findings, it seems reasonable to perform partial uncinectomy with identification of the maxillary sinus ostium, rinsing the maxillary sinus, and taking a swab for microbiological examination. In cases of more severe disease, for example uncinectomy, enlargement of the maxillary sinus ostium and anterior ethmoid surgery can be performed. Anatomical variations impairing the regular drainage of the paranasal sinuses should be corrected [846].
- In case of recurrences and/or more advanced disease, more extensive interventions up to “pansinus operation” must be discussed.
- Regarding CRSwNP, the same surgical concepts may apply for children as for adults.
- Antrochoanal polyps, mucoceles, or fungal sinusitis [857] generally call for surgical therapy [842].

The existing uncertainty concerning diagnosis, type and time of appropriate therapy require an individual analysis and decision making as well as intensive communication with the child and the parents.

Parents should be informed about the negative influence of passive smoking on the surgical outcome [858], [859], [860], [861].

Spontaneous regression of spheno-choanal polyps is possible [862].

Second-look surgery for postoperative examination is reserved to very special cases [863], [864].

In general, the growth of the facial bones of children is not impaired by endonasal endoscopic sinus surgery [517], [842], [865]. This seems also to be true for canine fossa approaches [319]. Nonetheless, depending on the extent of surgery, secondary hypoplasia of the operated paranasal sinuses is possible in single cases, however, without any consequence for e.g. visible symmetry of the child’s face [448], [866].

#### 4.4.9 Cystic fibrosis, primary ciliary dyskinesia

Endonasal endoscopic sinus surgery in cases of cystic fibrosis leads to significant improvement of sinonasal complaints and endoscopic findings [867]. There is no

improvement of lung function [867], [868], [869], [870]. The long-term recurrence rate of polyposis in cystic fibrosis amounts to 42–100% [842], [871], [872].

While some authors recommend a conservative surgical approach because of the expected high recurrence rate [873], [874], others plead for a more radical procedure with partial resection of the middle turbinate, creation of a large opening to the maxillary sinus (modified medial maxillectomy [875]) and to the frontal sinus (frontal sinus drainage type III [876], [877]), especially in cases of revisions [876], [878]. Beyond short-term success, the reported postoperative follow-up intervals do not suffice to evaluate if a more radical procedure actually leads to better long-term results.

The more aggressive procedure should allow a more complete removal of pathological tissue, facilitate local (antibiotic) therapy, and ensure passive drainage of the paranasal sinuses in cases of disturbed mucociliary clearance.

Postoperatively, nasal rinsing, topical nasal steroids, Dornase alfa, and long-lasting topical antibiotic therapy (Colistin, Tobramycin) are applied while also in this context necessary confirmations by literature reports are missing [867], [871], [868], [879], [880].

If preoperative sinus surgery helps avoiding re-colonization and infection of the lungs and positively influences the survival rate, is not finally clarified. One article in literature reports on negetive results [813], another one describes better results after intensive local therapy [814].

In cases of primary ciliary dyskinesia that often leads to chronic rhinosinusitis and to nasal polyposis in up to 30% no evidence-based statements on the effectiveness of endonasal endoscopic sinus surgery are possible because of missing studies [881]. According to the current literature, it remains unclear if sinus surgery is helpful [882]. Generally it could be discussed whether a possible surgical strategy could be adapted after failed drug therapy of irreversibly disturbed mucociliary clearance with resulting secretory stasis and its consequences [883].

#### 4.4.10 Neurectomy of the vidian nerve

Neurectomy of the vidian nerve is considered as a final therapeutic option in cases of therapy-resistant non-allergic and allergic rhinitis with the leading symptom of severely increased nasal secretion [884]. Nasal obstruction is only little influenced [885].

Precondition of effective surgery is the safe anatomical orientation with clear transection/coagulation of the nerve paying careful attention not to confuse it with e.g. the posterior pharyngeal nerve in the palatovaginal canal.

After first successful endoscopic neurectomies of the vidian nerve [886], [887], [888], meanwhile bigger case series have been published [884], [889], [890], [891], [892], whereas the technique is different in details. One variation is the presentation of the crista ethmoidalis and the sphenopalatine foramen, the transection of the branches of the sphenopalatine artery and the removal of the sphenoid process of the os palatinum [890]. The content of the pterygopalatine fossa is lateralized, subsequently, first the posterior pharyngeal nerve running in the palatovaginal canal appears and only few millimeters laterally the nerve of the pterygoid canal (vidian nerve) runs in a channel or canal in dorsal direction. The latter is completely transected and coagulated. The spheno-palatine artery may be preserved [892], [893].

Depending on the pneumatization of the sphenoid sinus and in order to protect the spheno-palatine artery, some authors recommend the intrasphenoid neurectomy in cases where the vidian nerve is prominent on the sphenoid sinus floor (type 1 and 2) and a special transsphenoid neurectomy whrere the nerve is impacted in the bone (type 3). The transection of the nerve is performed by means of a curved instrument or laser [884], [892], [894], [895], [896]. Alternatively, the vidian can be exposed via the superior nasal meatus [888].

A significant improvement of the symptoms is stated by 50–90% of the patients [884], [886], [888], [890], [892], [893], [897], while the results are obviously stable over several years [890], [893], [897]. Recurrences are explained by re-innervations that may also result from neighboring neural areas [898].

Data on side effects such as dry eyes, dry nose, and sensibility disorders in the area of the lips and the palate vary enormously. Immediately after successful transection of the vidian nerve a reduced lacrimation is observed [885], [886], [893], [899]. Clinically dry eyes are mentioned in 12–73% [884], [890], [892], [897] that usually disappear within few weeks to six months [900], [901] and only rarely persist (2.5% more than 6 months [892]). Bilateral keratopathy as a consequence of bilateral surgery occurs very rarely [902]. Temporarily a dry nose and crusting is found in 15–28% [884], [890] that only rarely persists. Sensory deficits in the area of the lips and palate are given with 3–22% [890], [891], [892], [897]. After 1–12 months they are regressive.

In summary, the data quality regarding the results of endoscopic neurectomy of the vidian nerve is not yet satisfactory because the assessment of the results and the follow-up intervals are very heterogeneous. Despite those limitations, neurectomy of the vidian nerve can be considered as safe surgery for therapy-refractory allergic and non-allergic rhinitis performed by an experienced sinus surgeon. As the majority of the patients are long-term satisfied or even very satisfied and the side effects are mostly temporary and can be met by conservative therapy, the respective type of surgery may be recommended as ultima ratio.

The reflection and the hypothesis that a more distal transection of post-ganglionic nerves in the area of the lateral nasal wall below the sphenopalatine foramen achieves safe results and few side effects [903], needs detailed confirmation in literature.

#### 4.4.11 Fungus ball

Fungus balls require surgical therapy with complete removal of all concretions and establishing an unimpaired ventilation and drainage of the affected paranasal sinus(es) [904], [905], [906], [907]. Local or systemic antimycotic therapy is not necessary because the fungus ball represents an extramucosal fungal disease [904], [905], [906]. Most frequently, the maxillary sinus is affected, more rarely the sphenoid sinus, even more rarely the frontal and ethmoid sinuses [904], [905], [906], [907], [908].

The surgical removal is performed endonasally, an external approach is no longer justified apart from very special cases [905], [907].

Rates of recurrence or residual disease amount to 3–7% [908], they are only higher for the more rarely affected frontal sinus. 82% of those patients live without any complaints, 96% are satisfied [908].

The main difficulty and the primary scope of surgery is the complete removal of the fungus ball under direct view. If the maxillary sinus is involved, the fungus ball is removed by means of a middle meatal antrostomy with application of angular optics, curved suction devices, angled instruments, and rinsing with pressure. In case of unclear situations that cannot be overlooked, an additional approach via the inferior turbinate [909], the canine fossa [910], the pre-lacrimal access, or the so-called gaze technique, which means pushing forward the fungus ball by gaze inserted into the maxillary sinus, may be applied [304], [911].

Regarding the frontal sinus, a drainage type IIa is performed. If the fungus ball cannot be removed safely, an extended frontal sinus drainage (type IIb or type III) are considered, if needed an external approach via minitrepanation at the anterior medial wall. Regarding the sphenoid sinus, the paraseptal approach is expected to be least invasive and most rapid [906].

#### 4.4.12 Respiratory epithelial adenomatoid hamartoma (REAH)

First described in 1995 by Wenig and Heffner [912], meanwhile more than 200 cases of REAH are reported in the literature [913], [914]. Hamartomas are pseudotumorous malformations that are characterized by pathologic differentiation or dislocation of regular tissue components with consecutive local overgrowth. REAH is a tumorous mass found in the nasal cavity, the nasopharynx, or the paranasal sinuses that is histologically described as proliferation of glands in the stroma covered by multi-row ciliary epithelium. Atypia is not found. In contrast to this, typically squamous epithelium invaginates in the underlying stroma in cases of inverted papillomas representing real tumors.

REAH can occur isolated or in combination with chronic polypoid rhinosinusitis, it can appear as unilateral or bilateral disease. Two third of the patients are male, an isolated location in the olfactory region is observed in about 20%. Widening of the olfactory region in the CT is a typical finding [915] that must let think of REAH especially when it is seen isolated. There is no enhancement in CT scan with contrast medium. MRI shows a hyperintense heterogeneous mass in T2 images. The T1 image reveals a hypo- or isointense mass in comparison to the brain with low-grade enhancement after contrast medium application.

Regarding differential diagnosis, inverted papillomas or low-grade adenocarcinomas of the paranasal sinuses must be excluded in order to avoid surgical overtreatment. In a few cases, also a coincidence of these diseases was described. It may even mask encephaloceles and esthesioneuroblastomas and in extreme cases it may extend through the olfactory region intracranially.

In the majority of the cases, the diagnosis was made histologically after surgery. It is important to consider also REAH in case of isolated polyposis in the area of the olfactory region, the nasal septum, the nasal cavity including the middle meatus or the nasopharynx. A biopsy should be the first measure. An atypic, rather berry-like appearance and a darker coloration compared to classical polyposis, and more solid consistency should lead to the idea of this differential diagnosis.

Therapeutically, surgical removal is indicated. After complete resection, the recurrence rate amounts to around 1%. The clinical significance of REAH that is diagnosed in the context of surgical therapy of CRSwNP, remains unclear. Prospective data whether there is a difference to patients without REAH, are not published [916].

#### 4.4.13 Silent sinus syndrome

Usually this diagnosis is made due to incidental findings, facial asymmetry, enophthalmos, inferor malposition of the eyeball or double vision without any clinically evident sinonasal inflammation [816], [917], [918], [919], [920]. Enophthalmos and inferior malposition of the eyeball are 3 mm on average [917], [920]. In the initial phase, obviously more often a sensation of pressure/pain of the maxilla occurs because of the increased negative pressure in the maxillary sinus [921], [922].

As a negative pressure in the maxillary sinus following obstruction of the natural drainage is considered as causative, therapy consists of endonasal endoscopic uncinectomy with enlargement of the natural ostium [816], [900], [920]. Wait-and-see strategy is not recommended because the deformity increases [919].

**Figure 13:**
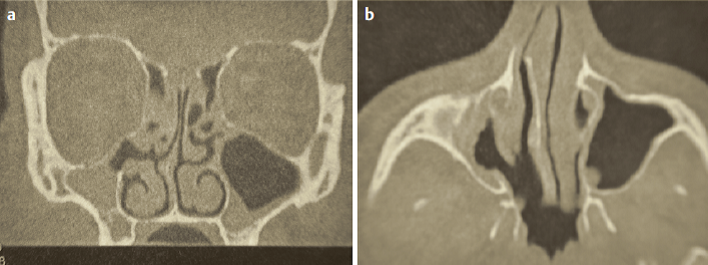
Silent sinus syndrome of the right maxillary sinus with a small maxillary sinus, lowered floor of the orbit and major retraction of the uncinate process that is directly close to the lamina papyracea

The altered anatomy with significant lateralization of the uncinate process and the lower position of the roof of the maxillary sinus have to be taken into account intraoperatively [923], [924] (Figure 13 (Fig. 13)). In single cases it is recommended to create the drainage opening via the inferior nasal meatus [816].

The lower position of the eyeball regresses by 1–2 mm after surgery [925], the enophthalmos by 2 mm (0.5–4 mm [901]). Thus it is reasonable to wait one year postoperatively and to correct the lower position of the bulb in a second intervention if needed [816], [901], [920].

A silent sinus syndrome of the ethmoid or frontal sinuses occurs much more rarely [926], [927]. Apparently it is based on the same pathological mechanism and should be treated by endonasal endoscopic surgery.

#### 4.4.14 “Sinogenic” headaches and facial pains

Often, patients present with recurrent or persisting pressure or pain in the frontal, periorbital, or midfacial region and trace it back to a mostly chronic sinusitis by own decision making or by medical consultations.

Up to now, the International Headache Society did not consider chronic rhinosinusitis as origin of headaches or midfacial pains as being sufficiently validated with the exceptions of pain correlated to an acute exacerbation of chronic rhinosinusitis [928].

Scientifically, a possible relation between sinonasal diseases and headaches was neglected [929]. More recent publications confirm an increased comorbidity of headaches or migraine and CRS [930], [931]. Different theories (immunologic switching, peripheral extension of a sensitization, allodynia) try to explain how a chronic stimulation of the peripheral trigeminal system may trigger or amplify migraine, and in cases of genetic disposition even might favor the emergence [929].

Also in the current International Classification of Headache Disorders [932], recent studies are mentioned that describe a correlation between persisting headaches and paranasal sinus pathologies. Up to now it is not sufficiently assessed that for example a condition after previous surgery with accumulation of mucus due to scarring may cause headaches and that paranasal sinus surgery may improve headaches [933], [934]. However, it must be mentioned that even patients with purulent nasal secretion do not suffer from headaches or facial pains in >80% [935], [936].

In order to accept a correlation of headaches with chronic or recurrent rhinosinusitis or other inflammatory diseases of the paranasal sinuses in the individual patient, the following conditions are required in combination with further symptoms [932]: 

A. Any headache fulfilling criterion C

B. Clinical, nasal endoscopic and/or imaging evidence of current or past infection or other inflammatory process within the paranasal sinuses

C. Evidence of causation demonstrated by at least two of the following:

1. Headache has developed in temporal relation to the onset of chronic rhinosinusitis.
2. Headache waxes and wanes in parallel with the degree of sinus congestion, drainage, and other symptoms of chronic rhinosinusitis.
3. Headache is exacerbated by pressure applied over the paranasal sinuses.
4. In the case of a unilateral rhinosinusitis, headache is localized ipsilateral to it.

There are no single markers that indicate accurately definitive sinogenic headaches.

Generally, patients with chronic pain and/or sensation of pressure in the midface, frontal and/or temporal region, should be examined by ENT specialists and neurologists [937]. 

The background is that large studies could show that patients with “sinogenic” headaches have in >80% of the cases a neurological origin of those headaches, mostly migraine or tension headache [929], [937]. 80% of those patients – revealing inconspicuous findings of the paranasal sinuses – respond well to a probatory therapy with triptans [937], [938], [939]. Patients suffering from migraine report in up to >50% nasal symptoms like nasal obstruction or runny nose [936], [937]. In contrast to facial pressure, facial pain rather indicate a neurological origin, as well as pulsating quality of the pain and a simultaneous sensitivity to light [940]. In cases of parallel olfactory disorders and postnasal secretion, however, a clear association to chronic rhinosinusitis may be anticipated.

Regarding the question of indication for surgery for persisting headaches and facial pain, the following procedure seems to be reasonable according to evidence in current literature:

- Precise anamnesis of headache
- Comprehensive ENT- and neurological examination, if needed also examination by a dentist/maxillofacial surgeon
- If nasal endoscopy and CT scan remain without pathologic findings, there is no indication for any type of sinonasal surgery [936]
- Clinically often negative pressure headache is regarded as an indicating symptom. This type of headache, however, may only be stated if the patient reports on (repeated episodes of) localized pain occur during landing with an airplane or resurfacing after diving (aerosinusitis, [629]).
- If endoscopic findings and/or CT findings correlate with the headache (e.g. purulent secretion, edema or polyposis; condition after previous surgery with e.g. irregular scarring) and complaints and findings persist after drug therapy (see above), an individually adapted endoscopic sinus surgery is indicated.

The existence and therapy of the so-called contact headache is controversially discussed [937], [941], [942]. A contact point is defined as an anatomical site where two opposite mucosal surfaces within the nasal cavity directly touch each other (especially septal spur, inferior turbinate, medialized middle or superior turbinate).

The endoscopic presentation of a so-called contact point and the reduction of the pains by topical anesthesia are no sufficient diagnostic criteria [929], [942]. Short-term surgical success irrespective to a negative test with anesthesia has been reported in literature and may be explained by cognitive dissonance and neuroplasticity [941].

Patients with endonasal contact points who otherwise have inconspicuous findings and CT scans of the paranasal sinuses and who do not respond to neurological migraine therapy, may be offered surgery. The benefit and the risks should be discussed intensively and it must explicitly be mentioned that surgical success cannot be guaranteed [937], [941], [942].

In case of tension headache and special types of midfacial segment pain [943], a specific process of the central nerve system (sensitization) may be assumed, e.g. by long-lasting nociceptive impulses (peripheral neural lesions, inflammation, trauma, surgery) that may lead (with other cofactors) to suppresssion of supraspinal inhibitory neuronal action. Any additional surgical intervention is likely to enhance this sensitization even if short-term pain reduction may be expected immediately postoperatively [943]. Thus, surgery on the paranasal sinuses by way of trial must be must be indicated very reluctantly (see above). They might be performed in close cooperation with a cooperating neurologist.

#### 4.4.15 Stenosis of the lacrimal system – dacryocystorhinostomy

Endonasal endoscopic dacryocystorhinostomy (DCR) is established as surgical method in cases of postsaccal dacryostenosis. Depending on the surgical technique in comparison to the so-called gold standard, the external surgery of the lacrimal system according to Toti, it leads to equivalent results with a success rate of 87% [944]. The quality of life is significantly improved [945].

The application of drilling systems (mechanical DCR) leads to better surgical results in comparison to laser-assisted DCR (success rate of 77%; relative risk of 0.85; [944], [946], [947]), even if the results in the last years have considerably improved [948]. Duration of surgery is reduced in laser-assisted DCR (19 vs. 60 min. in cases of external DCR [949]). For endonasal mechanical DCR, durations are set with 25–30 min. on the average [950].

Benefits of endonasal DCR are the avoidance of external scars, the preservation of the integrity of the lacrimal pump, a lower surgery-related morbidity, and the possibility of simultaneous correction of intranasal diseases or anatomical variations [951]. After external DCR, a visible external scar is reported in 19.3%, a cosmetic impairment in 10.3% [952]. 

To a high percentage, the uncinate process and the agger nasi cells are superimposing the lacrimal sac or an ipsilateral septal deviation which interferes with the DCR is found [953], [954]. Furthermore it must be observed that the major part of the lacrimal sac reaches on average 8 mm above the attachment of the middle turbinate in cranial direction [955].

- Silicone stenting is not recommended after primary endonasal DCR according to several systematic review articles because the results without stenting are at least equivalent [956], [957], [958], [959]. Stenting-related complications (endonasal crusting, granulations, infection, lesion of the canaliculus or lacrimal punctum, irritation of the cornea, discomfort of the patient) can be avoided [958]. Currently stenting is only recommended with (additional) presaccal/functional stenosis [960], [961].
- For functional dacryostenosis, significantly poorer results of simple DCR are reported (66 vs. 96% [960]). Stenting in cases of doubtful patency of the canalis communis during intraoperative examination can improve the results [960]. Preoperative exploration, dacryocystography, and scintigraphy are not able to predict this situation [960].
- While a meta-analysis revealed a failure rate in primary external DCR (relative risk of 0.51) and endonasal revision DCR (relative risk of 0.43) that was significantly lower due to the local application of MMC, no influence could be detected in cases of primary endonasal DCT [962]. Another meta-analysis that included more studies (less strict inclusion criteria including two non-randomized controlled studies) showed a minimally higher success rate of primary endonasal DCR (relative risk of 1.09; 95% CI 1.00–1.18), a higher success rate of endonasal revision DCR (relative risk of 1.21; 95% CI 1.02–1.45) and an increased endonasal opening of the lacrimal sac after 3, 6, and 12 months because of local application of MMC. After 12 months it was no longer significant [959]. The applied dose amounted to 0.2–0.5 mg/ml, the duration of application was mostly 3–5 min. (range of 2–15) [959], [963]. Specific side effects have not been reported [959].
- After application of mucosal flaps, the neo-ostium shrinks during the first 4 weeks by 20–25% and remains stable for the next 12 months [964].
- Important surgery determinants for the success of the intervention are the creation of big fenestrations and the use of mucosal flaps to cover bare bone edge-to-edge [274], [965], [966], [967], [968], [969], [970], [971].
- Furthermore the necessity of local postoperative care is reduced in DCR performed with mucosal flaps [965].
- A blind sac in the nasolacrimal duct in caudal direction must be avoided [961], [972].
- The success rates depend on the experience of the surgeon [973].
- Postoperative care after endonasal DCR is performed by means of eye-drops containing steroids and antibiotics for 2 weeks, topical nasal steroids, nasal rinsing with saline solution, and weekly performed instrumental cleaning. Routinely applied systemic antibiotics are not necessary [972]. An ipsilateral occlusion of the nasal cavity is reasonable (see chapter on postoperative care).

Recurrences of postsaccal dacryostenoses can be operated more easily by an endonasal rather than an external approach [959], [974], [975], [976], [977]. The endonasal approach allows a better identification and correction of the local anatomy responsible for failure [974]. Reasons for DCR revisions were: false location of the lacrimal sac during primary surgery, too small bony fenestration, insufficient opening of the lacrimal sac, granulations, scarring or osteoneogenesis at the neo-ostium, residual ethmoid cells causing local obstruction [978], [979]. The results are slighter worse than those of primary DCR (revision results of 80–85% [977], 69–100% [980]). 

Endocanaliicular laser-assisted revision is not recommended with regard to clearly lower success rates in comparison to external revision [981]. The use of laser leads to delayed wound healing while there is not clear advantage of a particular laser system [946].

Endonasal endoscopic DCR as immediate therapy in case of acute dacryocystitis with empyema of the lacrimal sac avoids the risk of cutaneous fistula and is expected to minimize the risk of orbital complications. It achieves a high success rate of up to 96.5% [971], [982]. Even in children the results of endonasal DCR are comparably good with a success rate of about 90% compared to external DCR [983].

In summary, today the endonasal endoscopic DCR applying conventional instruments is the surgical therapy of choice in cases of postsaccal dacryostenosis, primarily and in case of recurrences. It is crucial that a large bony fenestration of the lacrimal sac is created, that anatomical structures impairing lacrimal drainage are removed, that exposed bone and wound surfaces are avoided (by using mucosal flaps). The application of Mitomycin C may be discussed in exceptional cases (recurrence situation) as well as the insertion of silicone stents (presaccal stenosis).

#### 4.4.16 Tumors of the nose and paranasal sinuses

The following chapter may complement extensive literature reports on all relevant sinunasal tumors [9], [720], focusing on some of the most important clinical aspects of endosnasal endoscopic surgery and the most frequent occurring tumor entities.

Today, numerous benign and malignant tumors can be successfully treated by endonasal endoscopic surgery with at least equivalent results concerning complete resection of the tumor compared to traditional external procedures [9], [720] (Table 4 (Tab. 4)).

The step-by-step removal of a tumor (piecemeal resection or tumor disassembly vs. en-bloc surgery) does not compromise the oncologic result if a clear R0 resection is achieved [9].

Important features of endonasal surgery are are a good visualization (preferably and necessarily 4-hands technique, resepctively), sufficient hemostasis, safe reconstruction of the defect, and experience in the treatment of vascular complications [9]. The experience of the surgical team is another important factor for the choice of the approach. Depending on the extent of the tumor, the cooperation with neurosurgeons (see the complementary review on rhino-neurosurgery [4]), ophthalmologists, and maxillofacial surgeons is desirable or even necessary.

The primary rule and target is the complete and curative resection of any malignant (or benign) tumor. This concept should not be subjected to dogmatic reflections.

Usually, a patient is focused on his disease and is primarily interested in the possibly curative treatment, followed by aspects of function and post-therapeutic morbidity as well as finally aesthetic reflections. Any patient will sum up all the aspects mentioned when he chooses therapy and also the surgical approach following intensive counselling.

According to the current status, for the majority of sinonasal malignomas of stage T1 and T2 and for some malignomas staged T3, the extended endonasal endoscopic surgery is a useful alternative compared to open surgical procedures. Some other tumors of of stage T4+, may be subjected to “debulking” [9], [984]. The limits of endoscopic surgery are currently not yet defined. 

Generally the following regions are considered as being difficult to treat by endonasal endoscopic surgery and thus often represent an exclusion criterion for isolated endoscopic approach [9]:

- Lacrimal system
- Orbita – extension beyond the periorbit into the orbital fat
- Frontal sinus – significant involvement of the mucosa and each bone affection
- Maxillary sinus – involvement of the bony walls apart from the medial one
- Significant extension into the pterygopalatine and infratemporal fossa
- Extension in caudal direction through the nasal floor and the maxillary sinus with involvement of the hard palate, superior alveoli, and maxillary teeth
- Infiltration of the bony nasal skeleton
- Intracranial – significant involvement of the dura, infiltration of the superior sagittal sinus, infiltration of brain tissue
- Nasopharynx – significant involvement.

The single tumor entities are described intensively in other publications [9], [720], current complementary information is found for single tumors:

Adenoid-cystic carcinoma [985], adenocarcinoma [986], [987], [988], [989], [990], acinar cell carcinoma [991], chondrosarcoma [992], squamous cell carcinoma [993], malignant melanoma [994], esthesio-neuroblastoma [995].

#### 4.4.17 Benign tumors – osteoma

Osteomas are the most frequently occurring benign tumors of the paranasal sinuses [720]. Nearly 50% of all osteomas do not show any growth [996], [997]. The others reveal a median growth rate of 0.8–1 mm/year (range 0.117 mm/year to 6 mm/year; 95% confidence interval: 0.0004–0.230; [996], [997], [998]). Up to now, malignant transformation has not been described. The symptoms develop because of their expansion in intraorbital or intracranial direction as well as obstruction of the drainage pathways of single paranasal sinuses (most frequently the frontal sinus). Those are among others: diplopia, epiphora, facial deformity, blindness, intracranial complications, chronic rhinosinusitis or recurrent acute rhinosinusitis, development of mucoceles. Apparently, an osteoma per se does not cause pain [996], [998] but the inflammation or mucus accumulation because of drainage obstruction does [9].

Small asymptomatic osteomas usually do not require treatment. Control CT scan to determine the growth rate seems to be indicated in intervals of 2 years [996].

Most symptomatic osteomas are found in the frontal sinus and the frontal recess [999], [1000]. Recent classifications of frontal sinus osteomas [1001], [1002] try to define indications of surgery and to compare the results. Indications of surgery can be reasonably summarized: rapid tumor growth (>1 mm per year seems to be an appropriate value) and symptomatic patients (see above). It may be expected that the preoperative symptoms disappear postoperatively in around 90% of the cases [999].

Depending on tumor characteristics (attachment and extension), anatomy of the paranasal sinuses, experience of the surgeon, individual factors of the patient (comorbidities, anticipated duration of surgery) and the expectable surgery-related morbidity, an endonasal, an external, or a combined approach is chosen [9], [1003], [1004], [1005], [1006]. 

The smaller the anterior-posterior diameter of the frontal sinus, the narrower the anterior ethmoid sinus, the more lateral, cranial or anterior the osteoma reaches, the bigger the area of origin in the frontal sinus and the more the osteoma grows in intracranial and intraorbital direction, the more appropriate an external approach seems to be, preferably performed in the sense of osteoplastic surgery via a coronal incision or a frontal skin fold [426], [818] or a suitable orbital access.

Classical findings for indicating an external procedure are the following: extension in lateral direction to a sagittal level through the lamina papyracea, adhesion at the posterior or anterior wall of the frontal sinus, intracranial extension, anterior-posterior diameter of the frontal sinus <1 cm, >50% or complete obliteration of the frontal sinus, advanced protrusion in intraorbital direction [999], [1001], [1005], [1007], [1008], [1009], [1010].

If the surgeon is very experienced, even very large tumors penetrating the whole frontal sinus, sometimes also with insertion in front or superiorly can be removed endonasally via frontal sinus drainage type III [grade III-IV osteomas according to [1001]; [999], [1004], [1009], [1010], [1011], [1012]. Precondition is a suitable anatomy whereby the interorbital distance [1011] and the ratio of anterior-posterior diameter of the frontal sinus opening to the total size of the frontal sinus [1012] are of high relevance. Improved instruments and devices, e.g. powerful curved drills, help to shift the limits of endonasal surgery step-by-step [1011], [1012]. Obviously the most important limitation is the growth in frontal direction into the anterior wall with the probable necessity of reconstruction [1011].

Advantages of an external approach in cases of frontal sinus osteoma are a better overview and mostly shorter durations of surgery. The endoscopic approach better allows to control and verify a free endonasal drainage pathway at the end of the operation [999]. As external approach, osteoplastic frontal sinus surgery is recommended [426], [720], [818] whereas it must be individually discussed if preservation of the drainage or obliteration or cranialization [426], [1013], [1014] should be performed. In the individual case, any dogmatism with regard to certain types of approach is inappropriate. Decision making has to be performed on an individual basis [1012]. 

By means of suitable drills, the tumor is centrally excavated step-by-step until the remaining capsule of the exophytic part can be removed (cavitation technique, [1007]). The often relatively small area of attachment should be drilled until normal bone or underlying soft tissue (dura, periorbita, mucosa) appears. It must be considered that postoperative scarring leads to increased shrinking of the frontal “neo-ostium” because of the large bony wound surface. If the tumor is completely removed, covering the bare bone with mucosal transplantants or temporary insertion of silicone sheets must be discussed. 

Because of the benign nature of the tumor and its unknown or often missing growth, leaving small residues is preferred to a complete resection if it is associated with a significant increase of the morbidity. After resection of intraorbital osteomas, a reconstruction of the lamina papyracea is not necessary if the periorbit remains intact or has only small-sized lesions [1012], [1015]. Only in cases of larger, however, not exactly quantified defects, reconstruction is indicated to avoid diplopia. It is performed for example by means of fascia lata, which is fixed by sutures, or a nasoseptal flap [1015]. Postoperative controls should be performed endoscopically with the question of residuum/recurrence or development of mucoceles. If the findings remain unclear even after use of a flexible endoscope, CT scan/CBT seem to be indicated one year postoperatively provided that therapeutic cosequences may have to be discussed.

An **osteofibroma** as differential diagnosis of osteoma, is characterized by a tumor capsule that has to be removed because recurrences can be expected with high probability [720], [1016].

#### 4.4.18 Benign tumors – inverted papilloma

Inverted papillomas show some particularities that must be resepected in the context of type and technique of surgical therapy.

- The inverted papilloma is primarily malignant in about 7% (synchronously) so that the suspected presence of an inverted papilloma should always lead to surgery unless important patient-related factors object to it.
- Secondary metachronous malignant transformation is described in about 4%, in case of recurrences in 11%. The time of carcinoma development amounts 52 months on the average [1017]. In combination with the special growth behavior, the inverted papilloma requires a consequent surgical procedure to minimize the risk of recurrences. The inverted papilloma penetrates in a finger-shaped form into the mucosa and the underlying bone or neighboring soft tissue. Thus, the subperiostal removal of the tumor is the method of choice [9], [1018], [1019], [1020], [1021], [1022], [1023], [1024]. This means that the bone (the CT scan often shows thickened bone at the sides of attachment) is either resected at the attachment (e.g. lamina papyracea, middle turbinate, ethmoid lamella) or drilled until clearly normal bone (completely white) appears (maxillary sinus, skull base). Dura and periorbit should possibly be preserved because they serve as an effective barrier against penetration [1023].
- Furthermore it is recommended to resect, comparably to malignant tumors, the surrounding normally appearing mucosa with margins of at least 1 cm around the attachment of the tumor because microscopic tumor cell groups may be present in the margins [1025], [1026]. Recent investigations, however, could not confirm this aspect [1027].
- The incidental occurrence of inverted papillomas in “normal” polyps amounts to 0.0–0.92% (see chapter on histological examination). In those cases a topographic mapping is not possible. It seems to be reasonable not to perform immediate revision surgery because on one hand this would be unnecessarily radical, on the other hand it would probably not be radical enough at the unknown site of origin (bone treatment). It is recommended to wait for finishing wound healing and to perform for example after 3 months careful endoscopy [1003]. If endoscopy of the sinuses is clearly possible, a diagnosis regarding residual inverted papilloma or inconspicuous mucosa could usually be made. In doubtful cases, MRI is appropriate as an additional diagnostic tool. The determination of the SCC antigen (squamous cell carcinoma antigen) turned out to be a good parameter of the disease course and is also indicated for unclear papilloma recurrences [1028], [1029], [1030], [1031]. The ratio of SCCA2/SCCA1 can be understood as hint to a malignant transformation [1029].

In general, the recurrence rate is given with 10–20% with the endonasal endoscopic procedure having a lower recurrence rate than the external approach [9], [1017], [1026], [1032], [1033]. Advantages of the endoscopic technique are the avoidance of external scars and facial swelling and a lower postoperative morbidity (pain, numbness). Intraoperatively, modern HD video-endoscopy allows a better view on the tumor and the margins of the healthy mucosa [720].

Specialized centers report about recurrence rates below 10% and draw the conclusion that it is due to the subperiostal surgical technique [1021], [1022], [1034]. First results of a pre-lacrimal approach show recurrence rates of 0–10% (0/7 [1035], 1/10 [304] ) if the maxillary sinus is involved.

There is no general clearly defined algorithm for the surgical therapy of inverted papillomas [1020], only individually adapted surgical strategies that are based on the subperiostal surgery technique [9], [1018], [1019], [1020], [1021], [1022], [1023], [1024]. The access is usually performed primarily via the endonasal route under endoscopical guidance [1034].

**Figure 14:**
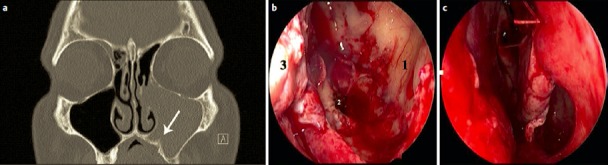
Inverted papilloma of the left maxillary sinus (Krouse stage III). a) Complete opacification of the maxillary sinus by exophytic tumor mass with typical hyperostosis at the small-sized origin at the base of the maxillary sinus (white arrow). b) After resection of the exophytic tumor mass the region of origin is exposed via a prelacrimal approach. Removal of mucosa with 1–1.5 cm safety margins and removal of the bone lying under the tumorattachment. Additional coagulation of an artery of the underlying mucosa of the hard palate. 1 = anterior wall of the maxillary sinus, 2 = region of the removed tumor-affected bone with coagulation after arterial bleeding, 3 = medialized inferior turbinate with nasolacrimal duct. c) Condition after repositioning and fixation of the inferior turbinate. The maxillary sinus can be well accessed via medial maxillectomy for endoscopic control.

The surgical principle consists of tumor-adapted piecemeal resection of the exophytic tumor mass with precise identification of the site of origin. The tumor is resected subperiostally with safety margins (1–1.5 cm seem to be appropriate). The underlying bone is removed or drilled in order to remove tumor parts penetrating into the bone (Figure 14 (Fig. 14)). Frozen sections allow identifying the tumor entity, dignity, and verify complete tumor resection. The blockwise histological examination of defined specimens containing resected tumor and mucosa should allow precise topographic mapping with regard to malignant parts in the final histology.

For this purpose, a surgical access is needed allowing the subperiostal preparation and the drilling of the underlying bone. A non-endoscopic endonasal approach is considered as being obsolete because of the high recurrence rate [720], [1032], [1036]. An external or combined approach is chosen if the tumor cannot be completely removed by endoscopy while clearance of the site of tumor origin is the crucial point. The ongoing refinement of endoscopical techniques has allowed extension of the range of indications for endonasal resections. [1032].

The traditional approach in cases of inverted papilloma of the maxillary sinus via a transoral sublabial access or a midfacial degloving or lateral rhinotomy are due to the fact that the tumor could not be sufficiently explored via the middle and even inferior meatus nor it could be removed. Beside disadvantages such as visible scars and the risk of injury of the infraorbital nerve, also the Caldwell-Luc approach bears the problem that the anterior wall and the floor of the maxillary sinus cannot be well exposed.

Since the introduction of endoscopic medial maxillectomy and its further refinements, today the maxillary sinus can be completely overseen by endonasal endoscopy. The use of angled instruments and devices, especially shavers and drills allows manipulation in all recesses of the maxillary sinus, apart from exceptional anatomic variations. An external access to the maxillary sinus for better control of the tumor, as still recommended some years ago [1025], is no longer justified with regard to new developments of surgical techniques (medial maxillectomy, transseptal approach, prelacrimal approach; see chapter on the type of surgery – maxillary sinus). Only in rare cases of particular anatomical variations (major protrusion of the infraorbital nerve into the maxillary sinus and hereof laterally attached tumor), a complementary minimally invasive external approach may be required. 

Depending on the individual tumor growth and regional anatomy, a tumor extent into the frontal sinus requires frontal sinus drainage type IIa, IIb, or III [1021], [1023]. A supraorbital expansion may require coagulation and transection of the anterior ethmoid artery. A relevant involvement of the frontal sinus and the orbital roof are limitations of the endonasal approach and usually make an external procedure necessary, e.g. via an osteoplastic approach [1021], [1025], [1032], [1034]. Obliteration of the frontal sinus should be avoided in order to allow endoscopic control examinations [1032].

An extensive affection of the inferior-lateral sphenoid sinus may require a trans-pterygoid approach.

Control examinations are recommended for at least 3 years, in analogy to the follow-up of malignant tumors [9], [1037]. Duration and intervals of the control examinations should reasonably depend on the tumor itself and the types of surgery applied. The following factors justify longer durations of follow-up in shorter intervals despite R0 resection at the time of first intervention:

- Extensive tumor growth, recurrences, uncertain complete resection, combined approach, or histological hints of aggressive growth (hyperkeratosis, squamous cell metaplasia, high mitotic index, carcinoma in situ, multicenter growth).

The local control is performed primarily by endoscopy every 3–6 months during the first 2 years, afterwards every 6–12 months. If the tumor region cannot be assessed, MRI is initiated every 6–24 months [9], [1021], [1022], [1037].

The most important long-term complications are development of mucoceles, pain, and dry nose.

Even if most recurrences of inverted papillomas occur within 2 years, recurrences after more than 5–10 years are not uncommon [1017] so that long-term controls are recommended also because of possible secondary malignant transformation. Probably recurrences are rather residues [720], [1038]. After incomplete resection, the risk of malignant transformation increases [1001].

A crucial factor to achieve complete resection is the diligence of the surgeon [9], [720]. The resection of an inverted papilloma should be performed by an endoscopically experienced ENT surgeon [1019] because it becomes usually obvious only during surgery carried out with extraordinary precision, where the base of the tumor is located and which therapeutic measures should be reasonably taken (extended endonasal approach, in single cases additional external access, therapy of complications etc.). Surgery should only be performed if the preconditions regarding the surgical technique and devices are given to continue with the endonasally started intervention via the middle meatus in the sense of extended surgery so that the tumor can be completely identified and removed. With regard to the high recurrence rate of inverted papillomas in case of incomplete resection and the possible primary and secondary malignancy, a primary procedure with exclusive use of headlight or microscope for a suspected tumor can no longer be justified.

#### 4.4.19 Benign tumors – juvenile angiofibroma of the nasopharynx

Juvenile nasopharyngeal angiofibroma (=JNA) is characterized by a locally aggressive and destructive growth originating from the sphenopalatine foramen and the basis sphenoid to expand in direction of the nasopharynx, paranasal sinuses, orbit, skull base, and endocranium.

Currently, the surgical removal is the therapy of choice. The endonasal endoscopic approach is meanwhile considered as approach of choice for most patients [1039] especially in case of smaller tumors and extension into the infratemporal fossa, orbit, parasellar region [1040]. Specialized centers report on complete resection of advanced and intracranial tumors [1040], [1041]. An external approach is frequently (additionally) applied when the internal carotid artery, the optic nerve, or the dura are relevantly affected by the tumor growth [1040]. More advanced tumors are still a particular surgical challenge, irrespective of the approach [720].

With the further development and improvement of surgical techniques, devices, and instruments, the differential indication for endonasal or external approaches is shifted more and more to endonasal endoscopic procedures [1042].

A systematic review of the literature in order to compare exclusively endoscopic, endoscopically assisted or open resection of JNA revealed the lowest recurrence rate and the lowest blood loss for the exclusively endoscopic approach [1043]. The recurrence rate amounts to around 10% [1039].

- To perform successful surgery, the specific features of the individual JNA must be considered [1044], [1045].
- Sense and necessity of preoperative embolization are discussed controversially [1044]. A recent meta-analysis on endoscopic therapy of JNA revealed a minimal blood loss when embolization is applied, however, because of missing prospective comparative studies a clear recommendation could not be given [1039].
- Important surgical measures are the reduction of intraoperative bleeding by targeted early occlusion of afferent vessels and the reduction of the tumor by means of coagulating devices or laser systems in order to minimize substantial bleeding from vessels near the surface [1044].
- A precise preoperative imaging must assess the possible multi-lobular expansion into different directions that is often observed [1044]. Intraoperative navigation is recommended [417], [1046].
- An important surgical aspect is the necessity of subperiostal removal and intensive drilling of the basis sphenoid and the vidian canal beyond the visible infiltration in order to reduce the recurrence rate [274], [720], [1040], [1047].

It seems to be very useful to centralize the treatment of this tumor entity in specialized institutions to not only optimize the therapeutic outcomes (low recurrence rate, low intra- and postoperative morbidity, less long-term effects) but to promote essential research especially of this tumor.

#### 4.4.20 Benign tumors – fibrous dysplasia

Because of the rarity of the disease, only few data are present on the treatment of fibrous dysplasia. Current common consensus is not to submit asymptomatic patients to prophylactic surgery [1048]. Even prophylactic decompression of the optic nerve is not recommended [1048], [1049]. As fibrous dysplasia can also increase after completed skeletal growth, regular controls are appropriate [1048]. In case of symptomatic patients, the aggressiveness of the surgical therapy must be discussed individually considering the associated morbidity.

#### 4.4.21 Tumors of the orbita

During the last years, endoscopic surgery of orbital tumors has developed in addition to transfacial, transorbital, and neurosurgical approaches [1015], [1050], [1051], [1052], [1053], [1054].

Among other factors it requires high experience regarding endonasal endoscopic sinus surgery and reasonably the application of the 4-hands technique.

The endonasal approach is especially useful in cases of medial intraconal tumors, tumors of the orbital apex, and tumors directly located at the paranasal sinuses [1055]. A detailed analysis of the intraorbital anatomy is essential [1055], [1056]. Special techniques allow the enlargement of the manipulation possibilities by displacement of the medial rectus muscle [1054].

## 5 Technical equipment

Performing surgery requires an adequate technical equipment taking into account the defined objective of surgery. According to the current standard, those are among others:

- Endoscopes as optical devices (0° optics and 30° or 45° optics, possibly also 70° optics)
- HD video endoscopy if surgery is performed via a monitor
- Broad range of conventional mechanical (micro-)instruments. The selection must take into account the multitude of anatomical variations in order to allow the intended removal of the tissue [1057], [1058]
- Optionally shaver, navigation, balloon dilatation, laser, radiofrequency, and others.

The rapid advances of technical development causes the situation that after writing this manuscript, already new devices are available that are not described here. For daily routine, technical equipment is relevant that helps (better) achieving the surgical aim in a reliable way. The focus of the following paragraphs will be to present the currently known benefit of technical devices based on the available evidence. The user always has to check critically if benefit-risk-cost analysis is positive. 

### 5.1 Conventional mechanical instruments – through cutting instruments

Conventional mechanical instruments have been consequently further developed by constructing increasingly delicate and cutting instruments and such working around the corner. Aspects regarding processing and hygiene (disassembly, possibility of flushing) represent special features that that have to be considered.

Theoretically and practically, it is useful to remove tissue most precisely and specifically. For this purpose cutting forceps and punches are appropriate. It is avoided that incidentally pieces of healthy mucosa are removed and too much bare bone is exposed. This is especially true for the frontal recess and the opening of the frontal sinus with the risk of increased scarring and stenosis caused hereby.

Irrespective of this fact, up to now the statement was given that based on one single study a scientific evidence of increased effectiveness of cutting instruments was not given [1059], [1060], [1061] (see also chapter on microdebrider). After an average of 12 years and in accordance to previous analysis, there was no difference of the sides regarding single symptoms, the total endoscopic appearance, the CT findings, and the revision rate. Only synechia developed significantly less on the side where the cutting instruments had been applied [1059] without having an influence on ventilation and drainage and the surgical outcome. The prevalence and location of synechiae was different after 1 and after 12 years, because intercurrent some synechiae were no longer present and also new ones arose.

Among other aspects, it must be mentioned critically that no cutting instruments were applied for frontal sinus surgery (surgery was performed from 1997–1998) and that a mixture of different indications was given (CRSwNP, CRSsNP, RARS) with different pathophysiology and surgical strategy.

Additionally to the actual interpretation, the conclusion may be drawn that cutting instruments instruments probably lead to less synechia and scarring. This holds true also for surgery of the frontal sinus. 

This justifies the recommendation to apply cutting instruments in particularly sensitive areas of sinus surgery. The precision of tissue removal is higher. 

After precise cutting out of bony lamellas wound surfaces are restricted to the base of the bony lamella. However, a reliable longer-lasting functionality of the cutting action of the instruments is needed which causes significant costs for constant instrument management and repair. These functional and economic aspects require future scientific analysis.

### 5.2 Balloon dilatation

Balloon dilatation of the paranasal sinuses (also called balloon sinuplasty, even if “a kind of plasty“ of the sinus itself is not performed) was introduced in the market in 2005. Central part of the procedure is the insertion of a balloon catheter with dilatation of the ventilation and drainage of the maxillary, frontal, and sphenoid sinuses [1062], [1063], [1064].

It is an innovation in sinus surgery that was most intensively discussed during the last years – not only because this method has been promoted by broad presentation in the media and patients placed high expectations in this technique [1065], [1066], [1067], [1068], but also because up to now the actual benefit of balloon dilatation for therapy of sinonasal diseases is not sufficiently clarified despite a multitude of existing publications [1064], [1069], [1070].

Central problems of evaluating balloon dilatation concern for example the methods of the investigations: no clearly mentioned and defined indication [1062], [1071], [1072], [1073], [1074], [1075], retrospective studies [1067], [1072], [1076], [1077], [1078], mixture of different diseases [627], [853], [1067], [1074], [1078], [1079], [1080], [1081], mixture of exclusively performed balloon dilatation and hybrid interventions or other therapies [852], [853], [1062], [1071], [1072], [1074], [1076], [1079], [1081], [1082], [1083], [1084]. Sometimes critical comments were given that focused on the methods of the published articles on balloon dilatation [1085], [1086].

Many publications were written by authors who had a financial relationship to the manufacturer of balloon catheters or who were members of its staff [627], [854], [1062], [1063], [1071], [1072], [1073], [1074], [1079], [1080], [1087], [1088], [1089], [1090], [1091], [1092], [1093], [1094], [1095], [1096], [1097], [1098]. 

Generally, balloon dilatation seems to be indicated,

- if there is a sufficient anatomical access to the drainage pathway of the sinus to be dilated (middle meatus, frontal recess, sphenoethmoid recess).
- if an opening exists to insert the guide wire.
- if the ostium or the drainage area can be dilated.
- if the disease requires the improvement of ventilation and drainage as relevant part of the treatment.

Balloon dilatation is indisputably a very safe procedure, the complication rate is given with 0.01% related to the patients. Single case reports mention septal hematoma during dilatation of the sphenoid sinus, dura lesions [1064], [1066], [1099], orbital lesions (lamina papyracea), [1064], [1066], [1100], [1101], intraoperative cardiac arrest [1102].

Numerous case series, retro- and prospective studies with very different patients, report consistently high success rates [1062], [1071], [1072], [1073], [1074], [1080], [1103]:

- Dilatation of the single sinuses is possibly partly in much more than 90%.
- The accesses remain open in 80% to >90% according to endoscopic and CT findings.
- An improvement of the symptoms is achieved in 85–95%, even persisting for more than 2 years.
- The quality of life increases significantly after surgery.
- The incidence of acute infections, the consumption of drugs, and absences at work decrease.
- The revision rate amounts short-term to 1–2% and increases within 2 years to up to 9% (patient-related) [1073].
- Technical failures occur in 1.7–3.4% of the cases [1062], [1074].

Comparably good results are reported about transantral balloon dilatation of the maxillary sinus ostium and the ethmoid infundibulum in cases of circumscribed therapy-refractory chronic sinusitis of the maxillary sinus and sometimes the anterior ethmoid [627], [1087], [1088], [1093], [1094], [1096], [1097]: technical success in >90%, open access to the maxillary sinus in 96% according to CT scans after 3 months, even after 1–2 years persisting improvement of the quality of life, reduction of acute infections, drug consumption, and absences at work with increase of productivity. 

This intervention can be performed under local anesthesia in more than 90% [627], [1094].

A special complication of the transoral technique may be a persisting numbness in the area of the infraorbital nerve in up to 7% [627], [1094]. The revision rate after 1 year amounted to 4–6% and after 2 years to 7% [627], [1095], [1097].

After exclusive balloon dilatation, patients have less pain in comparison to endoscopic sinus surgery, less postoperative bleeding events, and return to daily routine earlier, mostly within 48 hours [1067], [1071], [1073], [1088]. Instrumental cleaning as postoperative care is not required by the majority of the patients (86–92%) [1088], [1089]. The average need for cleaning is reported to be 0.1–0.2 [1088], [1089] or 0.4–0.8 per patient [627], [1071], [1073] over all patients or 1.1 per patient [1089] for those who required postoperative care. Patients after FESS or hybrid interventions need one additional postoperative care [627], [1071], [1088].

More and more reports are published regarding the application of local anesthesia and outpatient surgery [627], [1080], [1089], [1091], [1104], [1105].

- In >80-90%, balloon dilatation can be performed under local anesthesia.
- In >80%, patients describe it as at least tolerable.
- The average pain intensity on a VAS ranging from 0–10 amounts to 2.7–5.8 [627], [1088], [1089].

#### Application in children

Nearly exclusively, data of one center are present [852], [853], [854], [1106] regarding children with so-called therapy-refractory chronic rhinosinusitis that do not allow final assessment [1070] apart from the fact that balloon dilatation is a safe procedure. Complications did not occur. In cases of normal maxillary sinuses the procedure is technically possible in about 90%; in cases of maxillary sinus hypoplasia and generally for the frontal and sphenoid sinus only in around 60% [853], [1106]. In addition to adenotomy, balloon dilatation of the maxillary sinus ostium with rinsing achieves better results as expected [854] (see also chapter on sinus surgery in children). Another retrospective study did not reveal a significant difference of the success rates, measuring single or total complaints in comparison to exclusive endoscopic sinus surgery or in combination with balloon dilatation in children with therapy-refractory chronic rhinosinusitis [1077]. The children who had been treated with additional balloon dilatation needed postoperatively less antibiotics and had a better nasal breathing. Children with preoperative headaches benefit more from exclusive surgery of the paranasal sinuses.

##### Application in diseases of the frontal sinus

A multicenter study reports about a failure rate of 12% regarding dilatation of the frontal sinus [1076]. After frontal sinus dilatation by means of balloon dilatation 38% of the patients reported about persisting headaches [1107]. 71% of the accesses were open, further 17% required recurrent cortisone application. Only 48% showed an improvement in the CT scan (!), in case of existing risk factors such as Samter’s triad only 36% [1079]. Hereby, balloon dilatation was applied in the context of hybrid surgery.

CRSwNP is no indication for balloon dilatation of the frontal sinus [1064].

Small case series or single case reports describe the successful application in cases of:

- Silent sinus syndrome ([1108] – follow-up of 3 months; [1109] – follow-up of 1 week to 4 months),
- intensive care patients [1110],
- frontal sinus mucoceles [1105], [1111],
- acute frontal sinusitis [1112],
- Pott’s puffy tumor [1113],
- sinogenic headaches [1082],
- frontal sinusitis, treated with retrograde balloon dilatatio via minigrapanation [1114].

An extension of the transantral technique with 2 trepanation openings in the anterior wall of the maxillary sinus and dilatation of the posterior ethmoid is described [1115]. This does not seem to be necessarily less invasive than an endoscopic transnasal approach.

The continuance of fractured and loose bone lamellas of the basal lamella of the ethmoid bulla and the basal lamella of the middle turbinate is not clarified. Examinations of cadavers revealed that during balloon dilatation the anterior wall of the ethmoid bulla fractures in 56%, while the frontoethmoid cells do not fracture [1116]. The space achieved by frontal sinus drainage type I or type IIa surgery was significantly larger than the one achieved by balloon dilatation.

Balloon dilatation of the maxillary sinus led to a “via falsa” in the area of the posterior posterior fontanel in all 10 cases [1117] and in one case to submucous positioning during balloon dilatation of the frontal sinus. The application of a 5 mm balloon dilated the ostia from 1.7 to 3.6 mm.

Anatomical calculations were performed to optimize the technique of balloon dilatation [1118].

Reflections on radioprotection during ballooning procedures are no longer valid due to the introduction of the LUMA technique [1078], [1119] and navigated guide wires. Nevertheless, the measured irradiation exposition is very low for the patients (eye, total dose) and the ENT surgeon (hand, total dose, breast) and it is significantly below the critical threshold dose or the natural radiation exposure, however, it must be observed nonetheless [1119], [1120], [1121], [1122]. New developments concern a flexible tip, multifunctional dilatation products (probe, rinsing, and ballooning) [1123], the additional application of rinsing systems [1124], and navigable guiding catheters [1125] or guide wires.

Literature gives support to the postulation, that **any surgical innovations of the 21^st^ century must be evaluated critically, thoroughly, and scientifically** [1069].

Analysis according to evidence-based criteria come to the conclusion that there is no sufficient evidence for the effectiveness of balloon dilatation in cases of CRS, especially in comparison to usual endoscopic sinus surgery [1126], [1127]. The quality of most of the studies achieves only level 4 or grade C [1064], [1100]. The few, partly prospective comparative investigations [1067], [1084], [1128] also show significant methodical weaknesses so that definite conclusions cannot be drawn.

Special attention should be paid to the study performed in Graz, Austria, of 45 consecutive patients with therapy-refractory CRS and exclusion of eosinophilic CRS. In 2/3 of the cases, balloon dilatation with or without complementary conventional surgery (hybrid surgery) led to failure according to the study criteria so that the study was interrupted. The impact of previous interventions or so-called osteitic alterations was not confirmed [1129]. An angle of >90° between the cells of the frontal recess and the actual frontal sinus was associated with a higher failure rate. The cited study criteria were criticized by others – as they were regarded as responsible for the increased rate of interrupted dilatations, i.e. for failures [1085].

Indisputable is that the traumatization of the tissue is relatively lower in cases of balloon dilatation – a microtrauma caused by dilatation, however, must generally be expected regarding the intended fracture of bone lamellas and smaller mucosal bleedings after the procedure. Up to now, there is no (sufficient) evaluation regarding

- The incidence to which extent inflammatory wound healing reaction occurs (persisting inflammations, scarring, and osteoneogenesis, [1130]).
- The extent of which the dilated ostia shrink during the course of time.
- The measurable value of balloon dilatation in the context of hybrid surgery. Most balloon dilatations are performed as hybrid intervention [1072], [1131].
- The comparison if balloon dilatation in the context of hybrid technique is more atraumatic and successful than the consequent application of known and established surgical techniques of frontal sinus drainage type IIa [248], [268], [353], [358].
- The incidence of mucoceles occuring in the long term [1132].

The application of balloon dilatation must be critically questioned for revision surgeries because the reasons for “recurrence” are very different. The possibilities of balloon dilatations with regard to the underlying disease and the main complaints have to be individually analyzed taking into consideration the anatomy (width of the ethmoid sinus, anterior-posterior diameter of the frontal sinus opening) and the postoperative status (preserved uncinate process, preserved anterior ethmoid cells, lateralized middle turbinate, synechiae of the middle meatus, missed ostium).

The recommendation of routine application and indication of balloon dilatation instead of the above described Measures of endoscopic sinus surgery cannot be justified according to the currently published scientific data.

The fact that balloon dilatation can be indicated in particular cases (aerosinusitis, isolated sinusitis, single paranasal sinuses, intensive care patients, “simple” revision situations because of circumscribed membrane-like scarring), and in particular in the context of frontal sinus disease [1064], [1112], [1129], [1133].

The exact value of balloon dilatation in the context of surgical therapy of CRS is unclear. Especially for eosinophilic CRS and CRSwNP a concept of exclusive dilatation of the ventilation and drainage pathways contradicts to current knowledge on the pathophysiology [204], [205], [209], [1134].

Currently the statement of the guideline on “Rhinosinusitis” [3] is still valid:

“According to the German Society of Oto-Rhino-Laryngology, Head & Neck Surgery [1133], there is only a small spectrum of indications”. “With the precondition of an appropriate micro-anatomy, a dilatation of the frontal sinus access can be indicated in case of isolated recurrent acute or chronic frontal sinusitis. In some cases, also hybrid interventions in combination with conventional surgery of the ethmoid sinus and complementary balloon dilatation of the frontal sinus can be justified if the dilatation is expected to contribute substantially to securing an uneventful healing of the paranasal sinus system. Similar interventions in the area of the sphenoid sinus cannot yet be assessed, the dilatation of maxillary sinus ostia in routine cases is seen most critically.” The procedure is not generally covered by the general insurance system.

A self-dilating system based on osmosis should have the effect of further minimization of the trauma by dilating the maxillary sinus ostium over 60 minutes [1135]. It has to be mentioned that it is possible to fracture and penetration the lamina papyracea near the ostium.

### 5.3 Virtual endoscopy, 3D endoscopy

Virtual endoscopy can support the anatomical understanding as well as the diagnostics of a determined disease regarding the type and extent. In particular, endoscopy can be performed from the inside to the outside. Despite significant improvements, the quality of the images and the hereby retrieved information currently do not suffice to draw relevant diagnostic and therapeutic conclusions that may also be gained by already existing imaging techniques by means of flexible of rigid endoscopy as well as CT scan, CBT, or MRI [1136], [1137], [1138], [1139], [1140], [1141]. Thus the cost-benefit relation seems to be unfavorable up to now.

Usual 2D endoscopy with the monocular endoscope has the disadvantage of lower depth of field, hand-eye coordination, and poorer estimation of sizes and distances. For endoscopic surgery of the paranasal sinuses, this disadvantage is not only compensated at least partly by experience, anatomical knowledge, and haptic feedback, but also by continuously moving the endoscope [1142]. 3D endoscopy is expected to improve stereoscopic view. Currently either a 2 canal technology via camera or video chip or the shutter-technology are applied [1143]. Technique related side effects are possible. Apparently the newest generation of 3D endoscopes for endonasal sinus and skull base surgery has only minimal technique related side effects such as headaches, nausea, or sensitivity disorders of the eyes and shows first promising results [1143], [1144]. From a clinical point of view, no differences were found regarding blood loss, complications, or duration of the hospitalization [1145], [1146], [1147], [1148]. Limitations are currently still for example the narrow field of vision, a central darkness, and deterioration of the image in cases of impurities of the lens [1143], [1144], [1149] that make working with the 70° optics difficult [1150]. Cadaver dissections provided better results when the 3D endoscope was used in comparison to 2D endoscopy. Other studies could not reveal a difference between experienced surgeons and an improvement in certain parts of surgical manouvers in beginners [1150]. First experiences from the field of endoscopic skull base surgery show that surgery with exclusive guidance by 3D endoscopy is possible without increase in the number of complications [1148], [1151], the habituation to the new system occurs rapidly, and a precise anatomical orientation is provided [1152]. The presentation of colors by the system, however, seemed to be different, the 4-hands technique was difficult or impossible due to the autofocus function [1152]. Advantages are seen in the endoscopic rhino-neurosurgery where the available space is sufficiently wide. To which extent improved learning curves result in the training process for classical functional endoscopic sinus surgery and if surgical outcomes become better, must be proven.

As an additional and new feature of surgical navigation systems, procedures have been developed by which important target structures are preoperatively marked and intraoperatively included in the monitor image (“overlay endoscopy”) [1153]. Thus a color-coded spatial orientation taking not yet visible structures into account in the video-image is becoming possible. Modern navigation systems provide this option.

The change from conventional camera systems to immediate electronic image conversion at the tip of the endoscope (“chip-on-the-tip technology”) as well as endoscopy with completely variable angles of view are further technical developments. In this context, reliable data on daily relevance are not present.

### 5.4 Navigation

Navigation systems have the potential to improve anatomical orientation and are meant to reduce the complication rate as well as to increase the completeness of surgery. In the long-term, an improved quality of life for the patients and a reduction of revision surgeries should result.

The general preciseness of optic systems is better than the one of electromagnetic ones [1154], [1155]. The deviation under clinical conditions (target registration error) amounts to about 2 mm [448], [1155], [1156], [1157] and has to be differentiated from the frequently given in vitro deviation (fiducial registration error). At the beginning of each intervention and also during the course of surgery, the preciseness has to be verified.

For optic systems, the navigated instrument must be in direct visual contact with the system camera. In case of electromagnetic systems, this problem is not relevant, however, disturbances of the magnetic field can occur due to influence of other devices/instruments. Combined systems are increasingly offered. Risk zones and anatomical landmarks can be defined and visualized laid over the endoscopic image (“augmented reality”; the “overlay endoscopy” is one aspect of “augmented reality”, however, both terms are often used as synonyms) [1157]. Collision warning systems give optical and acoustical signals depending on the distance of the previously defined target structures [1158], [1159]. Even according measurements of the respective distance are displayed. The development of flexible navigated instruments can help reducing efforts and costs of navigation.

The navigation is susceptible to failures and not without fail, its preciseness can change also during surgery or depending on the location [183], [198]. Hence, always the current precision of the navigation system must be critically questioned so that regardless of its application profound anatomical knowledge and surgical expertise as well as a high degree of thoroughness are essential [198], [448], [1156].

A benefit-cost analysis revealed the increased costs of surgery due to the implementation of navigation. The higher time requirement because of preparatory efforts is possibly compensated by the advantages of improved anatomical orientation. The increased feeling of safety may mislead surgeons to proceed more aggressively [1160]. A systematic review including 6 studies did not demonstrate that the complication rate was reduced or the success rate was increased [1160]. The use of navigation was regarded as a type of surgery which probably has a benefit in selected cases. A similar conclusion was drawn in a meta-analysis of 8 studies [1161]. Based on a meta-analysis of 14 studies, the application of a navigation system led to a reduction of the rate of all complications (risk ratio 0.66; 95% CI 0.47–0.94), especially of severe complications (RR 0.48; 95% CI 0.28–0.82). Regarding the completeness of surgery, the necessity of revision surgery, and postoperative outcome, there was no significant difference [417]. Based on this analysis, a differentiated recommendation for the use of navigation systems was elaborated (Table 5 (Tab. 5)) that stated more precisely and improved the recommendations of the specific American Society [443]. The authors do not agree with some of the recommendation, e.g. in frontal sinus drainage type III, frontal sinus revision, pansinus operation, unless special complicating factors are present.

In summary, the use of navigation systems in routinely performed sinus surgery is still not necessary [417], [1160]. Independently, there are advantages regarding training, education, and teaching purposes [417], [448], [1162]. Up to now, an influence of the use of navigation systems on litigations could not be observed [1163].

### 5.5 Robotics

The general advantages of a robotic system are an improved endoscopic visualization (stability, 3D) and an increased precision combined with advanced mobility of the wrist. There are very promising first approaches [1164], [1165], [1166], [1167], [1168], [1169] [1170], [1171], [1172].

In summary, the current robotic systems are not sufficiently appropriate for surgery of the paranasal sinuses and the skull base because of manifold limitations [1164], [1170], [1173].

### 5.6 Shaver systems (microdebrider, powered instrumentation)

A shaver or microdebrider is an electrically powered, cylindrical suction-cutting device that sucks tissue continuously into a cylindrical tube and cuts it by oscillating or rotating knives in an interior cannula [193], [1174], [1175]. Numerous blades with different diameters, angular deflection, positioning of the canal opening, surface of the cutting inward cannula shall allow individualized, more precise, and effective tissue resection. New developments concern mechanisms of reducing obstruction in the suction canal that are very time consuming to remove, a bipolar coagulation function, and the navigation of the tip of the instrument [1176].

Targeted suction of tissue allows the precise removal of soft tissue (and thin bone structures), an incidental removal of larger mucosal areas can be avoided.

The targeted resection can be performed by means of curved blades, even around the corner. The appropriate shaver device allows a better continuous removal of polyps and exophytic tumor masses and a step-by-step approach to critical anatomical structures or the base or origin of lesions in comparison to conventional instruments. Another advantage is the continuous suction of blood and secretion from the surgery site so that mostly a good vision of the current field of interest is given – the bleeding itself, however, is not reduced.

The advantage of efficient and rapid removal of soft tissue which is sucked into the shaver canal, has one severe disadvantage in case of failure: if the tip of the instruments comes incidentally in contact with orbital or endocranial tissue, always a tissue resection occurs with subsequent substance defect. A quick reaction to avoid tissue resection is not possible. Regarding a rotation rate of 3,000/minute, the available time would be 1/50 s to react and definitely stop the device.

The missing tactile feedback during surgery may be considered as disadvantage or at least difference to working with established instruments.

So it is important to always have visual control of the opening of the tip of the shaver and to orient the instrument in that way that a maximum of safety and protection of critical structures is achieved [1177]. The shaver should reasonably be applied by surgeons who are experienced in conventional sinus surgery [1178].

If the integration of feedback systems and a navigated control that immediately stops the shaver when the safety limits of pre-defined resection areas are exceeded may compensate the mentioned general demerits of shaver systems, will be shown in the future [1155].

The statement was promoted that the shaver leads to better wound healing or less synechia in comparison to conventional, cold-cutting instruments. Up to now this assumption was not clearly confirmed. In 2 studies there was no effect [1179], [1180]. The anterior ethmoid and the antrostomy were comparable open. In 2 other studies, less synechiae were found on the shaver side after 4 weeks (14 vs. 22% [1165]) and after 18–60 months (6 vs. 14% [1181]).

In contrast to the assumption, after 6 months [1179] and after more than one year [1182], recurrences of polyposis were more often found on the side that was treated with the shaver, however, the shaver had always been used on the right side with a possible negative impact on the outcome.

The influence on the duration of surgery remains unclear: 2 randomized studies revealed a reduction of 37% in CRSwNP with comparable blood loss [1183] and of 11% [1181] on the shaver side. One randomized study found a reduction of 30% for the use of conventional instruments [1179], a non-randomized study revealed no difference with regard to duration of surgery and bleeding [1184].

In a controlled comparative study without randomization, significantly better results were found after 6 months in the shaver group regarding reduction of the symptoms [1184]. The application of the bipolar coagulation function led to a lower blood loss and a shorter duration of surgery in a controlled study enrolling 80 patients [1185].

In one investigation, patients were offered ambulant shaver polypectomy with a vacuum-powered device to palliate their complaints before regular sinus surgery. The application was successful in 87% and achieved a reduction of nasal obstruction of 43% [1186]. Surgery performed on an outpatient basis by means of shaver was possible in 80% of the patients with recurrent polyposis [1187] while 87% of the patients described the disturbance as rather low-grade, comparable to normal postoperative care.

Whereas the global complication rate regarding the application of shaver systems is similar in comparison of usual endoscopic sinus surgery and even big case series confirm this aspect [1188], the occurring complications are particular severe if the device is used inappropriately (resection of the medial rectus muscle with persisting diplopia, resection of the optic nerve or intracranial structures) [448], [1178], [1189], [1190], [1191], [1192], [1193], [1194].

In the majority of the cases, severe orbital and endocranial complications were not recognized intraoperatively [1177], [1192], [1195], [1196], [1197].

An extremely rare defective function of the electric instrument and unfavorable accompanying conditions (the body of the patient touches metal), may lead to an intraoperative electrical accident [1198].

According to the current state, the shaver is a useful additional instrument of sinus surgery that allows precise resection of mucosa, polyps [679], exophytic tumors [198], thin bone trabeculae etc. More difficult is the application for stronger ethmoid trabeculae, osteoneogenesis, or extensive scarring.

In case of exophytic benign and malignant tumors, the resection of the exophytic part may be helpful to identify the origin of the tumor [1199]. By means of a collecting container mounted to the device, the tissue could be seized for complete histological examination. A scientific evidence that confirms the superior precision, is not yet available. Benefit, risks, and costs have to be weighed out.

For effective removal of bones (e.g. in the context of extended frontal sinus, maxillary sinus, and ethmoid sinus surgery, at the skull base, for osteomas, dacryocystorhinostomy, decompression of the optic nerve) special drills can be used to remove the bone with varied aggressiveness. They are available with different angular deflections and sizes. For endonasal drilling, on the one hand usual drilling systems with long and delicate drilling handpieces and drills are applied. However, they only allow a straight working direction.

In comparison to traditional drilling systems, the drills of the shaver console have the advantage to dispose of distal rinsing and suction so that the surgery site can always be overlooked with the endoscope which increases the precision of bone removal and minimizes the risks. Overheating of the surgery site is avoided by continuous rinsing. The distal rinsing leads to a clear and precise endoscopic image. The deflection allows adaptation to the individual anatomy and drilling around the corner. No heat-related damage at the nasal entrance occurs.

Conventional drills with higher rotation (high-speed drills) are currently even more effective. Furthermore, wear and the necessity of possibly using several single-use drill heads for an individual patient cause costs that must not be neglected.

### 5.7 Laser

The various laser systems differ among others with regard to cutting action of tissue, coagulation, and carbonization, depending on the absorption properties and penetration depth [1155], [1200].

Optimal would be the removal of bone and soft tissue, precision of tissue resection of <1 mm, limited thermal depth effect, coagulation of vessels of >5 mm as well as fiberoptic application possibilities [1200].

Comparative investigations of endoscopic sinus surgery have not shown any advantage of laser application (Holmium:YAG laser [1200], KTP laser [1201]). It is not surprising that the application of laser leads to increased edema [1200], [1201], crusting, and a longer healing in comparison to conventional instruments because of thermal lesions [1200]. A problem in this context is the missing haptic feedback and the relevant mucosal damage with uncovered bone [1200], [1202].

Case series describe the application for recurrences of ethmoid sinus polyposis (KTP laser [1203], diode laser with intensified topical cortisone treatment (1,000 µg/day) [1204]), or in addition to surgery with conventional instruments (KTP laser, [1205]; Holmium:YAG laser [1202]). The Holmium:YAG laser is not suitable for nasal polyps [1205].

In summary, there is no sufficient evidence in the literature for rational laser application in endoscopic sinus surgery.

### 5.8 Other technical equipment

Radio frequency ablation (coblation) is used in transnasal tumor surgery using the hemostatic properties of this system and thus reducing the bleeding during resection of tumors that are mostly well supplied with blood [1176], [1206], [1207], [1208]. Also for revision surgeries in the context of CRSwNP or for endoscopic resection of encephaloceles the coblation technique led to a shorter duration of surgery and reduced bleeding [1209], [1210].

Coblation is a thermal procedure which may cause thermally induced side effects: increased pain, edema, damage of the mucociliary activity of macroscopically preserved mucosa, increased crusting [1209]. Up to now, the extent of thermal distribution during surgery is unclear. Application near the lamina papyracea should not be performed in order not to risk orbital thermal damage. Intraoperatively, whitening of the mucosa can be disturbing with regard to endoscopic orientation. It is also a disadvantage that bone lamellas cannot be removed. Regarding those numerous open questions and limitations, this procedure should not be applied in routine sinus surgery apart of special scientific investigations. 

Electrosurgical procedures may be helpful to perform mucosal incisions (nasoseptal or other mucosal flaps, DCR) inside the nose with reduced bleeding. A very small needle and low power should be applied (Thomas Kühnel, personal information).

Ultrasound guided aspiration works with the inverse piezoelectric effect which allows bone removal realized by continuous rinsing and suction. It causes less heat than conventional drills. Soft tissue is protected [1176], [1211]. The theoretical advantages of tissue selectivity are not sufficiently assessed up to now. Adequate data on heat development at the tip of the device are missing. There are descriptions about the reduction of the os turbinale of the inferior turbinate, ethmoid osteoma [1212], [1213], or the application in pituitary surgery [1214].

Intraoperative imaging is helpful to verify the completeness of surgery, especially in cases of tumors, but also in traumatology, orbital reconstruction, or inflammatory diseases. Further, the intraoperative navigation can be actualized [1215].

## 6 Results (outcome)

There are no generally acknowledged isolated parameters to define the success after endonasal sinus surgery [197]. The results can be assessed with regard to:

- patient satisfaction
- improvement of single symptoms
- endoscopic or CT/MRI findings
- general and disease specific quality of life
- recurrence rate
- rate of revision surgeries
- long-term patency of surgically created neo-ostia
- influence on drug consumption, costs of the disease, and absences at work
- complication rate ([448] and chapter on complications).

Systematic reviews and meta-analysis confirm the general safety and effectiveness of endonasal endoscopic sinus surgery for the therapy of CRS [19], [1216], [1217], [1218], [1219]. Another meta-analysis [1220] must be read critically because of methodical and textual weaknesses [21] – only 3 studies have been included and all of them did not call for an ineffective conservative trial prior to indication for surgery. One study remained unpublished [1220]. Generally it must be stated critically that the different patient populations were heterogeneous and the applied surgical techniques varied and the (recommended or necessary) drug therapies were not clearly defined.

The few published randomized, controlled studies show that the surgical therapy is at least as effective as drug therapy [1218], [1221]. In single studies, advantages become obvious regarding improved single nasal symptoms, endoscopic or postoperative CT findings [1218].

In a recent prospective comparative, non-randomized study, patients after endoscopic sinus surgery reported about a more significant improvement of their disease-specific quality of life than patients who had continued with drug therapy (odds ratio of 3.37). One third of the patients treated with drugs switched to the group undergoing surgery and observed a significant improvement postoperatively [1222].

From these studies, the conclusion can be drawn that endoscopic sinus surgery may be recommended and reserved to patients where drug therapy was not successful [19], [1218], [1221].

The significance of endonasal endoscopic sinus surgery in the treatment of CRS was revealed in a recent investigation on the effectiveness of maximal drug therapy of CRS.

After maximal drug therapy (systemic steroids for 3 weeks, topical steroids, nasal rinsing, and antibiotics for 3 weeks according to a smear test of purulent secretion), 50% of the patients reported about persisting complaints after an average of 6 months and underwent surgery in 86%. 38% of the patients were primarily symptom-free (64% of them had persisting opacification in CBT), 43% of them developed new complaints and 29% underwent surgery again. Thus a total of 50% underwent surgery. 14% of the total group were symptom-free without persisting opacification in the CT scan, in 12% another diagnosis than CRS was found [23]. This observation correlates with the results of the evaluation of a large database that showed that within 6 months 46.2% of all patients with known diagnosis of CRS and 34.3% of the patient with newly diagnosed CRS underwent surgery [1223].

Patients for whom surgery is planned after failed drug therapy, experience deterioration of their complaints, of the endoscopic findings, and an increase of absences at work during a waiting time of 7 months despite continued intensified drug therapy (nasal rinsing, cortisone spray, cortisone rinsing, antibiotics, systemic steroids, macrolides, antihistamines, leukotriene receptor antagonists, [21]).

Future studies will have to clarify if and which subgroups of CRS patients benefit more from an initial surgical therapy than from drug therapy, e.g. because the stage of an irreversible disease is achieved or in order to reduce or avoid the extension of the inflammatory process [208].

### 6.1 Patient satisfaction (total)

A large number of high-quality (level II–III) studies shows that treatment success in the sense of general improvement of the symptoms can be achieved in 75–95% of the cases by means of endoscopic sinus surgery after failed drug treatment [19], [933], [1216], [1217]. The effect size of the total improvement of the symptoms amounts to 1.19 (95% CI, 0.96–1.41).

Assessing the therapeutic effects, the following criteria are applied:

- An effect size of at least 0.8 means a strong effect [933], (https://en.wikipedia.org/wiki/Effect_size)
- An improvement of the VAS score is considered as clinically significant if an improvement of at least half of the initial value or an increase of 1.3 “points” (scale from 0–10) is observed [1224].

Two publications are worth being mentioned – one is retrospective, randomized, and controlled – where patients with symptoms of CRS after drug therapy and only low-grade findings in the CT scan (Lund-Mackay score of 0–6) stated an improvement in around 80% one year after endoscopic sinus surgery, comparable to the one of patients with more important symptoms (Lund-Mackay score of 7–24; [1225], [1226].

### 6.2 Improvement of single symptoms

Single nasal symptoms improve in >80% of the patients by 50–60%, while the results for nasal obstruction are more favorable than for reduced olfaction and postnasal secretion [395], [932], [1227], [1228]. The effect size for nasal obstruction amounts to 1.73, for facial pain and postnasal secretion to 1.19, for reduced olfaction 0.97, and for headaches 0.98 [934].

Fatigue and general physical pain as symptom frequently observed in patients with CRS also significantly improve after sinus surgery in CRS [934], [1229], while more relevant improvements can be expected in cases of more severe initial symptoms.

Regarding the symptom of headaches, there are contrary results and evaluations (see also chapter on sinogenic headaches). On the one hand, headaches are a frequent symptom that is often the most disturbing one [1224], and postoperatively a significant reduction of those headaches is observed [1230]. On the other hand, an improvement can also fail despite an improvement of all other symptoms of CRS [1224]. The reason may be that the headaches may have been not sinogenic, that the evaluation measure was not suitable, or that surgery could not improve sinogenic headaches [1224]. It was critically stated that there might be a financial relationship to the pharmaceutical industry of authors of relevant studies that come to the conclusion that a frontal headache was caused in the majority of the cases by migraine and other neurological types of headaches and that an appropriate drug therapy should be performed [934].

An improvement of olfaction is found less frequently in some more recent studies with 23–55% [1231], [1232], [1233] compared to earlier publications. In contrast, improvements were described in 79–87% of the patients [1234], [1235] and postoperative normosmia after preoperative hyposmia was observed in 70% [1235].

Generally, patients with anosmia and CRSwNP are more likely to experience a postoperative improvement of olfaction than patients with hyposmia and CRSsNP [1231], [1233], [1236], [1237], [1238], [1239], [1240]. These results indicate that a multifactorial pathophysiology can be suspected, e.g. an obstruction of the olfactory region and/or neuro-epithelial lesions. Normalization of olfaction is achieved more rarely. It is not possible to safely predict postoperative improvement [1240].

The possibility of deterioration of olfaction (hyposmia, anosmia) in up to 10% should be mentioned preoperatively [1232], [1234], [1235], [1236].

After frontal sinus drainage type III, an improvement of olfaction was observed in 57%, no change in 29%, and deterioration in 13% [1241].

The removal of polyps or parts of REAH from the olfactory region did not lead to an impaired olfaction. Previous interventions and partial resection of the middle turbinates were negative risk factors [1242].

Regarding the symptom of smelling, surgery and drug therapy in CRSwNP were superior to exclusive drug therapy in a prospective, non-randomized study [1243].

Endonasal endoscopic sinus surgery improves bronchial asthma (in 76% of the patients with 85% less asthma attacks), reduces the number of inpatient treatments (by 64%), and the drug consumption (oral steroids by 73%, topical steroids by 29%, bronchodilators by 36%). The pulmonary function parameters do not change significantly [1244].

Even if in single patients a low-grade improvement of the apnea-hypopnea index can be achieved, the endoscopic sinus surgery generally does not significantly influence obstructive sleep apnea [1245].

General sleep quality and sexual activity are positively influenced by endoscopic sinus surgery [1246], [1247].

### 6.3 Endoscopic or CT/MRI findings

Even if often a significant discrepancy between symptoms, endoscopic findings, and CT findings is observed [245], [469], [1219], [1248], endonasal endoscopic sinus surgery leads to a clear improvement of findings in the endoscopic aspect and radiologic imaging parallel to the improvement of complaints and the quality of life [1228], [1249], [1250], [1251].

### 6.4 General and disease-related quality of life

The quality of life of patients with CRS is clearly impaired and achieves values in according inventories that are poorer than those of patients with chronic diseases such as for example hypertonia or COPD [1252].

A series of studies shows that by means of endoscopic sinus surgery a general and disease-specific reduction of the quality of life can be significantly improved [235], [1219], [1253], [1254], [1255], [1256], [1257], [1258], [1259], while patients with CRSwNP reported about more relevant respective improvements [235], [1256].

Postoperatively, patients achieve nearly normal values [1253], [1255], [1260], [1261], [1262], [1263]. The achievement of normal values in inventories concerning the quality of life, however, does not mean that the symptoms have disappeared [1264]. For most of the patients, the results remain stable over the time [933], [1227], [1261], on the other hand the number of performed revision surgeries increases (see below; [235], [1265]). Assessments of the quality of life after 6 months correspond to those after 20 months [1266]. The total improvement of the quality of life amounts to 70–80%, about 80% of the patients report at least about an improvement by 50%, when solid criteria for the assessment of the success are used (improvement of ≥50% of the standard deviation of the basic score; [20]).

### 6.5 Recurrence and revision rate

Depending on the duration of postoperative care, the precision of the analysis, and the type of inflammation, the recurrence rate amounts to (4–)20–60% [3], [20], [235], [245], [323], [1216], [1217], [1247], [1251], [1265], [1267], [1268], [1269]. With time, this rate increases.

Revision surgery is indicated in about 20% within 5 years (4% after one year, 12% after 3 years; [236], more frequently in CRSwNP than in CRSsNP [235], [1227], [1265], [1267]).

The reason for revision surgery is often a disturbed drainage of the frontal recess or the frontal sinus neo-ostium caused by residual parts of the uncinate process and anterior ethmoid cells, a missed ostium of the maxillary sinus, a lateralized middle turbinate, scarring, osteoneogenesis, or recurrent polyposis [15], [16], [17], [18], [362], [363], [391], [469], [1270], [1271]. A major part of those intraoperative findings in revision surgeries is based on an insufficient surgical technique applied for initial surgery [363].

A missed ostium sequence can lead to recirculation through both ostia [469], [1271] with a predisposition to develop symptoms and infections [469]. Postoperatively it is not always clear if the missed ostium sequence occurred primarily at the time of previous surgery or secondarily by scarring. Patients with missed ostium sequence have more complaints than others [469].

Negative factors influencing the surgical outcome are [19], [1218]:

- Primary nature and extent of the disease [8], [249], [1218].
- Frontal sinus involvement, which increases the risk of recurrent polyposis or revision surgery to 1.4 or 1.6 [1265]. More extensive surgery with additional interventions on the frontal sinus could significantly reduce the rate of necessary revision surgeries (19.0 vs. 14.1%; [235]).
- Previous surgery: the success rates after revision surgery are lower than after initial surgery:
  - The success rates of general symptom assessment are reduced to about 70% [1272].
  - The probability of improved symptoms is twice as high after initial intervention than after revision [20].
  - The improvement of the quality of life after revision is lower, however, similar after each additional revision surgery [1273].
  - The relative risk of revision surgery after previous surgery amounted to 3.07 according to an extensive US database analysis [1274].
- Bronchial asthma [24], [323], [1227], [1265], [1275], even if the study results are partly inconsistent [1218].
- Intolerance of analgesics [24], [1219], [1249], [1276], [1277], [1278], [1279], [1280] and Samter’s triad (significantly more frequent revision surgery: 37% within 5 years and 89% within 10 years [1265]).
- Allergic fungal sinusitis [241], [1281].
- Evidence of staphylococcus infection with superantigens [1282], [1283], [1284], [1285], [1286], [1287], [1288], [1289] (see also complementary review by C. Bachert [201]).
- Evidence of biofilms [1290].
- So-called osteitis: A bone involvement with thickening of ethmoid trabeculae and walls of the paranasal sinuses correlates with the severity and extent of CRS or a condition after previous surgery [1291], [1292] and is associated with a poorer outcome [17], [1293], [1294], [1295], [1296], [1297]. Topical cortisone therapy by means of nasal rinsing can possibly compensate the negative influence [1298]. Suggested classification systems are not sufficiently validated [1291]. Currently it is unclear if it is a pathogenetic factor of the disease or mere a consequence [19], [1294], [1299] and which exact pathophysiological correlations exist [1291]. Recent histological results indicate that it is not an original inflammation of the bone but rather a process of tissue remodeling as reaction on mucosal inflammation or tissue trauma, so that the term of osteoneogenesis is more appropriate [1292]. It is recommended to remove areas of thickened bone whereby this is relatively easy or possible only in the ethmoid sinus and at the middle turbinate. On the other hand, the extent of necessary resection regarding optimal results is unclear [1291].
- Cystic fibrosis [869].
- Smoking: whereas smoking clearly contributes to CRS [1300], it is not sufficiently clarified if smoking or intensive smoking leads to postoperatively impaired quality of life and to more frequent and earlier revision surgery [22], [1301], [1302] or if it has no influence on postoperative complaints or long-term results [1284]. Smoking is no contraindication for endoscopic sinus surgery [1300].
- The influence of allergic rhinitis on the surgical outcome is currently not clearly defined. It is recommended to preoperatively optimize therapy of the allergy in order to improve the postoperative result [1218], [1303].
- Tissue eosinophilia and histological parameters: An increased tissue eosinophilia correlates with a poorer surgical result and increases the risk of recurrence of CRSwNP [20], [209], [285], [1269], [1304], [1305], [1306], [1307]. This aspect, however, was not confirmed by all studies [1308]. In patients with lower-grade tissue eosinophilia and reduced subepithelial edema, the postoperative improvement is two to four-times more likely [20]. Therapy with topical steroids improves the according postoperative results [209], [1309], [1310]. Even the number of goblet cells and thickening of the basal membrane are said to correlate with the severity of the disease and a poorer surgical outcome [1311], [1312].

Immunodeficiency is not a negative predictor, the results are comparable to those of patients with a normal immune system [19], [1313].

There is no convincing evidence that the gastro-esophageal reflux plays a causal role in the pathogenesis of CRS [1314], [1315] and influences recurrence and revision rates of endoscopic sinus surgery [1314], [1316]. Nonetheless, a gastro-esophageal reflux disease can contribute to the symptoms, especially postnasal secretion, for example via gastro-nasal reflux, which has to be considered in the context of drug therapy [469], [1314], [1315], [1316], [1317], [1318].

### 6.6 Long-term opening of the surgically created neo-ostia 

Maxillary sinus fenestration in the middle meatus (antrostomy) remains open in 85–98% [245], [249], [469], [1217], [1319]. The size of the surgically enlarged maxillary sinus ostium is reduced by wound healing processes within 12 weeks to 54% [282] of to 40% after 1–3 years [281] while the preserved maxillary sinus ostium has 80% of its original size after 1-3 years [281]. 

The ongoing progress of of endonasal endoscopic sinus surgery is shown especially in the results of frontal sinus surgery. Earlier publications could only find a postoperatively open frontal sinus neo-ostium in 30–40% by endoscopy. An open access was additionally confirmed by exploration with a probe or CT scan in 70–81%, regarding an improvement of the symptoms and clinical success rate of 83% [396], [1320], [1321].

More recent publications show that the frontal sinus neo-ostium after frontal sinus drainage type IIa remains open in 85–92% [361], [362], [391], [392], [393]. The size of the frontal sinus neo-ostium may reduce within 12 weeks to 31% because of wound healing processes [282] or to 65% within 6 months [394].

After frontal sinus drainage type I (that is not clearly defined in the surgical result, see chapter on frontal sinus surgery) and anterior ethmoidectomy in cases of chronic frontal sinusitis, a success was found in 88.5% of a heterogeneous patient population [360].

Regarding frontal sinus drainage type III, very different results are revealed (see also chapter on frontal sinus surgery):

- The patency rate varies between <70–97% [8], [240], [241], [243], [359], [372], [384].
- The revision rates range from 5–32% [240], [241], [242], [359], [397], [398], [399].
- The clinical success rates are more stable with around 80% of improvement [241], [243] , [244], [402] – they are similar to those of osteoplastic frontal sinus surgery [400].

Systematic investigations of the postoperative size of the sphenoid sinus neo-ostium are not present to the same amount as for the maxillary and frontal sinuses. The size of the sphenoid sinus neo-ostium is reduced within 12 weeks to 47% due to wound healing processes [282]. In a larger case series of isolated sphenoid sinus diseases, >90% of the neo-ostia were open postoperatively [1322].

### 6.7 Impact on the consumption of pharmaceuticals, disease-related costs and absences at work

The drug consumption decreases significantly after endonasal endoscopic sinus surgery:

- Antibiotics (weeks per year): reduction of 50–70% [621], [626], [1227], [1323] or 35% [622]
- Systemic steroids: 50–70% [1227], [1323]
- Topical steroids: 13% [1227]

The annual medical costs after endonasal sinus surgery amounted to only about 50% of those before surgery [626], [1274].

The loss of general productivity in patients suffering from therapy-refractory CRS are significant: 63.4 paid working days were calculated that were lost because of absences and reduced working capacity. About half an hour, patients spend every day for their disease which summarized to 21.2 days per year with total costs of 10,077.07 $ [1324]. Patients with CRS seek medical advice more frequently and thus cause higher medical costs than the healthy population [1325]. After failed maximal drug therapy, patients experience a deterioration of their complaints and an increased number of absences at work (more than double) during a waiting time of 7 months for surgery despite continued intensified drug therapy [21].

Disease-related absences (days) could be reduced from 1.9 to 0.4 in corresponding 3 month intervals by endoscopic sinus surgery [1323].

An economic model calculation under the assumption of a revision rate of 3% per year, costs caused by absences at work of 3%, and an inflation rate of 5%, revealed a break-even-point after 7 years [626]. Whereas a significant increase of the disease-related costs were observed as consequence of deterioration of the patient’s condition and the attempt of intensified drug therapy in the six months before surgery, the expenses decrease postoperatively to the level of the time before [1326]. Extending the observation period, a clear decrease of the disease-related costs is observed postoperatively in comparison to the year before surgery. However, the costs remain higher in comparison to the second year before surgery revealing the persisting chronic disease even after 4 years [1223].

Hence, endonasal endoscopic sinus surgery can be considered as economically successful, also on the long-term.

## 7 Postoperative care

The aim of postoperative care is to promote wound healing and early regeneration of the mucosa, to reduce local inflammation, and to minimize symptoms in the early postoperative phase [1327]. On the long-term, a persisting improvement of the quality of life and a minimization of revision surgery should result [1227], [1327].

Postoperative care after endonasal sinus surgery is integral part of the surgery [1247] and consists of instrumental cleaning, physical wound treatment (nasal rinsing, occlusion), local and systemic drug therapy [725], [1328], [1329], [1330], [1331] (Table 6 (Tab. 6)). 

Type and duration of treatment depend on

- the type of the underlying disease,
- performed surgery,
- patient-related factors,
- individual postoperative course.

### 7.1 Local instrumental postoperative care

Even if some studies questioned the value of postoperative care [1327], [1332], it is considered as sufficiently secured that the instrumental postoperative care improves the surgical outcome on the long-term (less adhesions, less complaints, higher quality of life). A local instrumental postoperative care should be performed [1333]. The first cleaning is performed according to several comparative studies most likely after one week. Further measures are taken individually, while weekly or longer intervals seem to be generally suitable [470], [1334], [1335], [1336], [1337]. This procedure is mostly international consensus [83], [199], [1227], [1228], [1327], [1329], [1330], [1331]. The performance of several cleanings of the paranasal sinuses within the first week is critically discussed [1330].

Only **endoscopically assisted** cleaning with application of appropriate instruments secures the necessary control of the neo-ostia and the removal of crusts, fibrin, coagulations, or beginning synechiae in the crucial areas of the paranasal sinuses. 

### 7.2 Local physical therapy (rinsing, occlusion)

The local physical and medical therapy is becoming more and more important [217], [1338], [1339], [1340]. An optimized access to the paranasal sinuses allows improved postoperative nasal rinsing [217], [218], [1341].

Concerning local therapy, a difference is made between vapors or drops (among others sprays, atomizers, nebulizers; so-called low-volume systems) and rinsing (the recommended dose is 200–250–250 ml per rinsing; so-called high-volume systems) [217]. Despite promising single studies of the protagonists of newly developed devices and medical products that intend to achieve a (better) deposition of drugs in the nose and paranasal sinuses, up to now inhalations and nebulizers are less effective for the postoperative care after sinus surgery in comparison to nasal rinsing [217], [1340], [1342], [1343] and thus may generally not be recommended [217].

Nasal rinsing with saline solution with the use of high volumes (about 250 ml) and compressible nasal douching devices are recommended from the first postoperative day on [217], [725], [1327], [1344]. It is plausible and recommended to rinse more often in the beginning (3–6 times), later-on less frequently (1–3 times). Generally isotonic or slightly hypertonic solutions and special salts are suitable. Emser Salz^®^ solution improves nasal symptoms and the quality of life on a long-term in comparison to no rinsing [1345]. 

Care must be taken that the users are well informed by physicians about the adequate application and about necessary cleaning of the nasal douche [1346], [1347].

**Figure 15:**
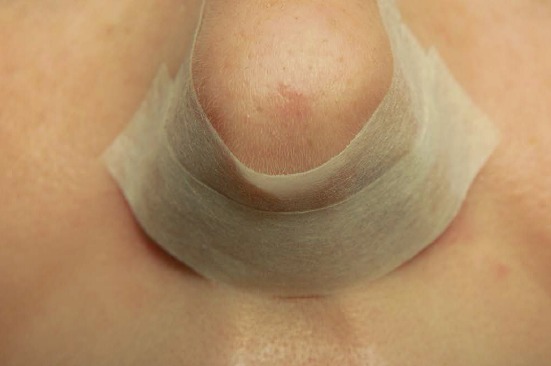
Occlusion of the nose for optimized wound healing

Occlusion of the nose is recommended because occlusion of wound surfaces maintains a humid wound environment and favorably influences wound healing [389], [1348], [1349], [1350]. Epithelization is accelerated, scarring is reduced, less postoperative pain and infections occur. The advantages of occlusion of the nose with accelerated epithelization, reduced crusting, and easier instrumental cleaning are known since many years [242], [296], [389], [390], [459]. For extended endonasal surgery, it is mostly integrated part of modern therapeutic concepts [242], [296], [384] (Figure 15 (Fig. 15)). The patient suffers from less pain, for the treating physician the effort of necessary instrumental cleaning and processing is reduced. While in cases of traditional frontal sinus drainage type III the average time of crusting and bare bony wound areas amount to about 6 weeks and the time of mucosal swelling to about 13 weeks [243], the application of free mucosal transplantations and occlusion required only 3 postoperative examinations on the average [384].

After extended frontal sinus drainage type III, current surgical concept recommend the covering of bare bone with free mucosal transplantations or pedicled mucosal transposition flaps [383], [385], [386], [387], [388], which leads to a significantly better healing process [384]. The according follow-up concepts after frontal sinus drainage type III include occlusion of the nose for 2 weeks, nasal rinsing after 1 week, and the first instrumental cleaning after 2 weeks. The systematic therapy depends on the underlying disease (see below).

### 7.3 Local medical therapy

An objective of endonasal endoscopic sinus surgery, especially in the context of therapy-refractory CRS is to improve access to the target area and to allow intensified local therapy [198], [209], [1327].

### 7.4 Local medical therapy – corticosteroids

Topical nasal steroids promote wound healing after endonasal sinus surgery [389], [1351], [1352] and are the basis of local anti-inflammatory therapy by reducing complaints and the recurrence risk of CRSwNP [217], [1329], [1340], [1353].

The concepts and schedules as of when topical nasal steroids have to be applied postoperatively vary between starting immediately after surgery and 2–6 weeks postoperatively [242], [725], [1247], [1327]. It seems to be reasonable to start topical steroid application after the first instrumental cleaning after one week because then a mucosal contact of the cortisone solution is obviously possible. Immediately after surgery can also be useful if crusting can be avoided by occlusion and intensive nasal rinsing.

Since cortisone spray does not reach important areas inside the nose [217], [1343], [1354], more and more often – especially in extended or therapy-refractory cases – the application of topical corticosteroids is performed by means of nasal rinsing [1340]. Systematic examinations could show that the nasal rinsing fluid is retained in 3.1 ± 1.9% of postoperative nasal rinsing and thus during rinsing with 1 mg Budesonid solution 2x per day about 50 µg each remain in situ. This corresponds to (half of) the dose of cortisone spray application (2 x 1–2 x 2 puffs) and represents only a small part of a systemically relevant cortisone dose [209], [1355]. Several studies could not reveal systemic side effects [1356], [1357], [1358], even in cases of significantly higher cortisone doses (3 mg Fluticasone 2x per day for 6 weeks, [1359]). It must be considered that this application is (still up to now) off-label.

The local application of cortisone drops into the frontal recess improves the patency rate of the frontal sinus access by 16% [1360].

### 7.5 Local medical therapy – antibacterial drugs/substances

In the focus of local therapy is among others the fight against bacterial biofilms [1288], [1361], [1362], [1363].

Local antibiotic therapy by spray application or nebulizers is not recommended [1340]. Regarding nasal rinsing with antibiotics there are currently no recommendations [1340]. In special cases of cystic fibrosis, postoperative Tobramycin rinsing can reduce the incidence of revision surgery [880].

The current research revealed that the following therapeutic approaches are safe and effective against *Staph. aureus* in first in vitro and in vivo investigations: manuka honey/glyoxal in a concentration of 0.9–1.8 mg/ml [1364], colloidal silver [1365], bacteriophages [1366].

Rinsing with 0.05% Mupirocin solution causes short-term improvement of the symptoms and reduction of staphylococci [1367], [1368], [1369]. After the end of therapy, however, re-colonization with deterioration of the symptoms was found [1367], [1368].

A clear recommendation is given against postoperative vasoconstrictive nasal drops to avoid rhinitis medicamentosa [1329].

### 7.6 Systemic therapy

In cases of advanced chronic rhinosinusitis, a postoperative systemic drug therapy with antibiotics and/or cortisone is a therapeutic option that is frequently applied [1247], [1327].

An application of antibiotic for 2 weeks could lead to accelerated wound healing and reduced crusting and nasal secretion [1370]. The application of antibiotics should be planned based on smear tests [1371].

Pre- and postoperative cortisone application leads to improved wound healing and a better endoscopic image as well as less symptoms for at least 6 months ([139]; 30 mg prednisolone for 5 days preoperatively until 9 days postoperatively). Alternatively, 20 mg are given for 14 days (own current therapy scheme), 30, 20, and 10 mg for 4 days each [1327], 25, 12.5, and 5 mg for 1 week each [23], or 25, 12.5, and 12.5 mg (every two days) for 1 week each [274], [361]. In cases of allergic fungal sinusitis, also a short-term postoperative systemic cortisone application is recommended [1372] starting with 0.4–1 mg/kg, e.g. 30, 20, and 10 mg for 4 days each [1372].

A long-term improvement of the therapeutic outcome, however, is not proven; the expected effects must be weighed out against the side effects and costs in the individual case.

For further systemic therapy of CRS see the complementary review of C. Bachert in this issue [201].

## 8 Nasal packing

The term “nasal packing” summarizes many different materials that are inserted into the nose at the end of sinonasal surgery or in cases of epistaxis [464], [1373], [1374], [1375], [1376]. Several decades ago, it was usual intention to stop stronger bleeding by local pressure. Numerous indications have been established and numerous effects of nasal packing have been claimed:

- Promotion of physiological wound healing by creating a humid environment, acceleration of epithelization, reduction of granulation and scarring
- Influence on wound healing and the disease leading to surgery by releasing drugs from the packing material
- Effect of stent by taking space
- Barrier function
- Even today hemostasis, directly or indirectly by induction or enhancement of physiological clotting.

Generally nasal packing can be avoided if endonasal sinus surgery is performed with careful hemostasis [464], [1374], [1377], [1378], [1379]. The patient has to be informed about the possibility of occasional low-grade bleeding in the postoperative period which does not require therapy [1374], [1380]. Furthermore, drying out of the nose by unimpaired nasal breathing leads to potential negative impact on wound healing that always proceeds better in a humid environment [389], [464]. Increased crusting because of dryness cause possibly pressure and nasal obstruction. An additional impairment, sometimes even associated with pain or secondary bleeding, occurs by the necessity to remove those crusts. Randomized controlled studies after endoscopic sinus surgery, however, are actually missing.

On the other hand, nasal packing applied short-term (few hours to max. 24 hours) provides generally a higher security to avoid annoying or in single cases even endangering (blood loss, cardio-vascular risks, shock, obstruction of the larynx with the risk of suffocation, aspiration of blood) postoperative bleeding and so it is reasonable to be applied routinely if the appropriate material is present. In the UK, packing was used in about 75% of sinus surgeries [236], [532], in Thuringia in 95% [545] A clear recommendation is given in the following cases [464], [1374]:

- Patients with coagulation disorders who have to undergo sinus surgery
- In case of persisting (diffuse) bleeding at the end of surgery despite careful coagulation at visible bleeding sites
- In case of increased risk of postoperative bleeding.

Packing is usually well tolerated even for longer hospitalization if it is performed in a justified way and the patient is informed accordingly [464], [1374], [1381].

Significant and necessary aspects are the creation of a best possible environment to provide and sustain unimpaired wound healing, the consideration of increasing patient requirements, and the avoidance of mucosal lesions or other unfavorable side effects by nasal packing [464], [1374].

Occlusion of the wound cavity establishing a humid environment by nasal packing promotes physiological wound healing.

Based on those aspects, the hemostatic/absorbable materials did not meet the expectations from different points of view [464] (see also chapter on synechia in the middle meatus). The fate of those materials has never been assessed systematically, a postulated complete absorption has not been confirmed. In contrast, apparently nasal rinsing, suction, swallowing, and the mucociliary transportation led to the disappearance of the material. The extent of spontaneous degradation has not been quantified in vivo.

For some of the materials, undesired side effects in the sense of increased granulation, scarring, osteoneogenesis, or incorporation in the regrowing mucosa of the paranasal sinuses could be revealed [464], [1382], [1383], [1384]. Recent randomized controlled studies confirm impressively that nasal packing with smooth or gel-like surface or coating improve the comfort, cause less pain and lesser bleeding when they are removed [1385], [1386], [1387], [1388], [1389], [1390], [1391].

In comparison to not applying packing, there were no advantages regarding wound healing parameters or bleeding for the application of carboxymethyl cellulose, hyaluronic acid, gelatin (gelfoam), or merogel [477], [1392], [1393], [1394], [1395], [1396], [1397], [1398].

Single reports state advantages with less synechiae for the application of carboxymethyl cellulose (CMC) [478], hyaluronic acid [1399], and chitosan-dextran gel [486]. The low sensation of pressure in the midface in the context of CMC application can be considered as consequence of occlusion with less extensive crusting [1394].

Chitosan-dextran gel is the only packing from the field of “absorbable” materials up to now that could lead to consistently positive results in a series of prospective, controlled, partly randomized, blinded studies without observing undesired side effects:

- Epithelization is accelerated, the adhesion rate is reduced (sheep model; [487])
- Postoperative hemostasis is more rapidly achieved (sheep model; [485])
- The adhesion rate is reduced, postoperative hemostasis is relevantly more rapidly achieved [486]
- Neo-ostia to the maxillary, sphenoid and especially to the frontal sinus (the latter after 12 weeks) are significantly more persistent [282].

Comparable results are reported from abdominal surgery [1400]. Chitosan-dextran gel has also antimicrobial properties [1401]. In systematic examinations it turned out not to be toxic, it is biocompatible, and has no pro-inflammatory characteristics [1400].

Significant advantages are expected from Chitosan with its properties promoting blood coagulation and wound healing as well as working as microbicide in combination with tranexamic acid [1402].

In summary there are reasonable indications for postoperative insertion of nasal packing. Because of the different indications, various materials and products are necessary to meet the individual requirements appropriately [464]. Packing that will be removed after some time, will have to have a smooth surface in order to minimize mucosal traumatization during removal. “Absorbable” (biodegradable) materials should at least provide the confirmation that wound healing is not negatively influenced. Wound healing in humid milieu is achieved alternatively to insertion of nasal packing by occlusion of the nose (Figure 15 (Fig. 15)).

### 8.1 Stenting/spacers

The conceptual transition from modern types of nasal packing to stents and spacers is fluent. While in earlier times those were always made of materials that remained in situ for a longer time and had to be removed, today more and more absorbable stent materials are provided [464], [1376].

The general sense of inserting stents or spacers is the separation of the wound surfaces, the use and support of defined anatomical spaces, the reduction of necessary instrumental cleaning (saving of time, comfort), the provision of a sheet for epithelial migration, and the effect as occlusive wound dressing [464], [1403], [1404]. New systems are rather planned for middle- or long-term drug application ([484], [1405]; see chapter on synechiae in the middle meatus).

### 8.2 Maxillary sinus spacer

Maxillary sinus stents for its opening in the middle meatus are described [307], [1406] and led to good results, however, because of the high success rate of maxillary sinus fenestration and improved surgical techniques they are not necessary. In difficult (revision) cases with narrow conditions in the middle meatus, modifications of the surgical technique seem to be more appropriate (see chapter on maxillary sinus surgery) than insertion of a stent because the stent may cause specific morbidity which must not be neglected.

### 8.3 Frontal sinus spacer

Nearly exclusively, stents are used in newly created and widened frontal sinus accesses. Regularly, a mucosal lesion is caused in the wound canal during the primary procedure and persisting coagulations and fibrin deposition are found. The stent is expected to work as a epithelization sheet for uncovered bone, avoid the development of coagulation and fibrin deposition, and reduce granulation. While a short-term application is sufficient to avoid fibrin and coagulation deposition, a long-term insertion of several months is necessary to influence subepithelial scarring until its final stages [807], [1407].

As indications for the insertion of stents in the frontal sinus access, the following aspects are mentioned in the literature: an intraoperative diameter of less than 5 mm, bare bone with large surface or in the whole circumference of the neo-ostium, the presence of allergic pansinusitis or advanced polyposis, a lateralized or floppy middle turbinate, extensive scarring or osteoneogenesis after previous surgery, and revision surgery [464], [473], [1403], [1404], [1408], [1409], [1410]. The insertion of frontal sinus stents for a longer time (6 months) led to an improved patency rate of the operated frontal sinus [389], [464], [820], [1407]. Small case series report that frontal sinus stents have been left for up to 5 years with only few complications (spontaneous dislocation in 14%, complete obstruction in 5%, necessary revision in 1/11 cases) [1409], [1410].

The mentioned advantages must be weighed out against the following disadvantages: induction of chronic inflammation by the foreign body, crusting, risk of forgetting and leaving the foreign body, possibility of dislocation to the exterior or into the frontal sinus. With this background, there is the alternative recommendation to make the concept of stents dispensible by applying better surgical techniques (endoscopic frontal sinus drainage type IIa, type III). Beside prefabricated tube-shaped stents that have the disadvantage to adapt only insufficiently to the wound surfaces and may cause too much pressure on parts of the irregular wound surface, individually cut foils are applied [242], [411], [464], [473], [1411].

Cortisone releasing stents lead to an improved patency of the frontal sinus neo-ostium [1405]. The application of doxycycline releasing stents was effective as well [1412]. Both systems have not been pursued because of pharmacological, economic, and other reasons. A stent consisting of Chitosan glycerol phosphate turned out to be apparently inert for the mucosa in an animal model and was able to release incorporated dexamethasone over 15 days or to eradicate bacteria in the infected maxillary sinus by means of incorporated antibiotic solution [1413], [1414].

In summary, the insertion of a stent in the opening of the frontal sinus can be helpful in individual rare cases. It is recommended to use a soft silicone material that does not exercise undue pressure on the wound surface. The ideal duration of the stent is unclear. A shorter duration suffices to reduce early postoperative reactions (deposition of blood coagulation and fibrin) and to accelerate first wound healing processes acting also as a moist wound dressing. A longer duration is favorable and necessary in order to include the phase of scar remodeling with e.g. the risk of development of stenosis in case of concave-round wound surfaces. In the case of persisting increased local reaction (granulation, purulent secretion, crusting) an early removal is recommended.

## 9 Education, training

Regarding the issue of education, an extensive statement was given recently [448]. Currently the anatomical dissection is considered to be the best preparation to perform surgery even if evidence-based criteria have not clearly confirmed their effectiveness [1415]. Young residents still prefer dissections, ranking second and third are surgical assistance and the study of videos or anatomical books [1416]. The most important aspect, still before learning manual skills, is the secure three-dimensional anatomical orientation [1416], [1417]. It is unclear to which extent dissections, simulations, assistance during surgeries, or anatomical studies are necessary to acquire a solid minimum of knowledge and capacity. Further there is no report in literature, which kind of advantage a surgeon will gain by performance of a single cadaver dissection on the occasion of a surgery course when he starts doing surgery as a separate person afterwards. 

A defined curriculum to achieve sufficient expertise does currently not exist even if first evaluation systems for assessing the surgical performance in sinus surgeries are present [1418], [1419], [1420]. By use of these instruments, also monitoring of the training stage is possible.

A standardized step-by-step education program seems to be a good basis that endonasal sinus surgery by beginners does not lead to increased complication rates [1421] nor to poorer surgical outcomes [1422] due to continuous surveillance.

Convincing evidence as of which minimum number of surgeries a surgeon may be called an expert, are not present [1419]. According to earlier publications, the achievement of the status of fellow was supposed after 100 interventions of the paranasal sinuses [7]. A current performance analysis considers a sufficient expertise for maxillary sinus fenestration and anterior ethmoid sinus surgery as well as posterior ethmoid and sphenoid sinus surgery as given after 23 interventions, for the frontal sinus after 33 interventions. After 42 sinus surgeries, there was a 60% probability of sufficient surgical competence which was considered as being sufficient [1423]. It is estimated that experts of music and sports have to have completed at least 10 years or 10,000 hours of intensive practice in order to achieve a good level – there are no investigations if the same is true for surgical expertise [1424].

Many simulators of different type are described while virtual systems require further development and reduction of the costs [1419], [1425], [1426], [1427], [1428]. The effectiveness of FESS simulators was shown for beginners whereby not all systems are sufficiently validated (yet) [1419]. The first available simulator, ES3 manufactured by Lockheed Martin, is no longer produced [1429], [1340]. Scientific studies looking for the evidence of clinical benefit have not been started [1419]. It can be expected that it will be possible to develop a surgery simulator in that way that not only an improvement of surgically manual knowhow and skills are achieved but also a realistic preparation is possible and cadaver dissection will be pushed to the background [1430].

The rapidly developing technique of 3D printer technique, in this context based on CT and MRI data [1431] must be observed, with regard to reduced costs and a realistic presentation of the material properties.

General basic surgical skills should be acquired outside the operating theater and the beginning surgeon should only perform surgery when he fulfills predefined criteria [1432]. Exercises with cheap abstract simulators are always helpful to train manual skills and may be part of a rhinologic skills lab [1428], [1433], [1434], [1435]. For the clinical routine, a simple, useful, and cheap surgical preparation consist of performing the instrumental postoperative care endoscopically with great thoroughness.

## Abbreviations

ARS – acute rhinosinusitis

BD – balloon dilation

CRS – chronic rhinosinusitis

CRSsNP – chronic rhinosinusitis without (“sine”) nasal polyps

CRSwNP – chronic rhinosinusitis with nasal polyps

CFT – canine-fossa trephine (transoral puncture of the maxillary sinus)

FESS – functional endoscopic sinus surgery

IP – inverted papilloma

MOS – missed ostium sequence

MAA – middle meatal antrostomy (via the middle meatus)

RARS – recurrent acute rhinosinusitis

REAH – respiratory epithelial adenomatoid hamartoma

SCC (A) – squamous cell carcinoma (antigen) 

## Notes

### Competing interests

With regard to instruments, devices and medical products used in the endonasal sinus surgery, the authors declare having personal and economic relationships to the following companies (in alphabetical order): ALK Abello, Berufsverband der HNO-Ärzte, Fentex, Infectopharm, Karl Storz, Klosterfrau, Medtronic, Neuwirth Medical Products, Olympus, Pari, Pohl Boskamp, Polyganics, Siemens & Co., Spiggle & Theis, Sutter, Vostra.

## Figures and Tables

**Table 1:**
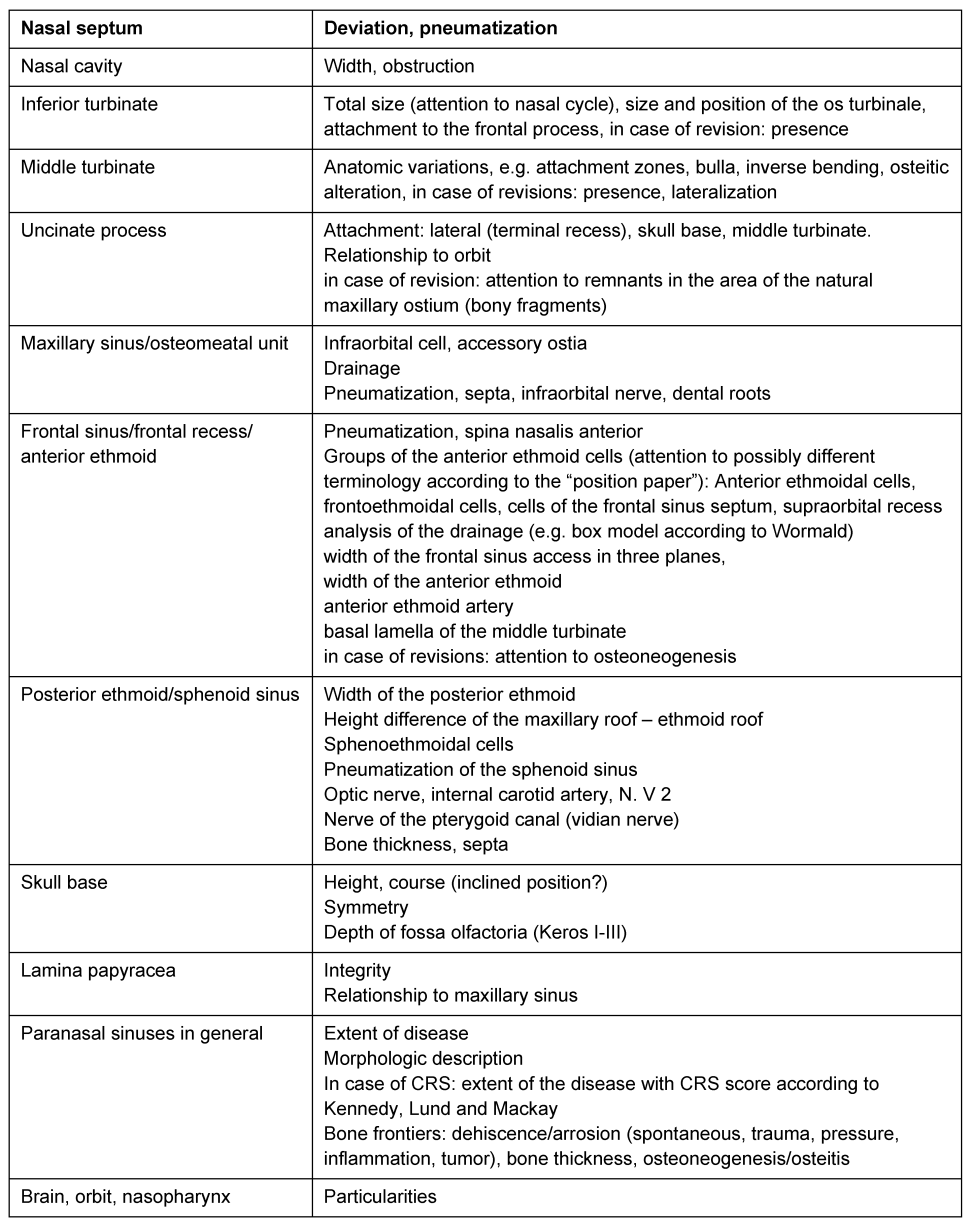
CT checklist before sinus surgery

**Table 2:**
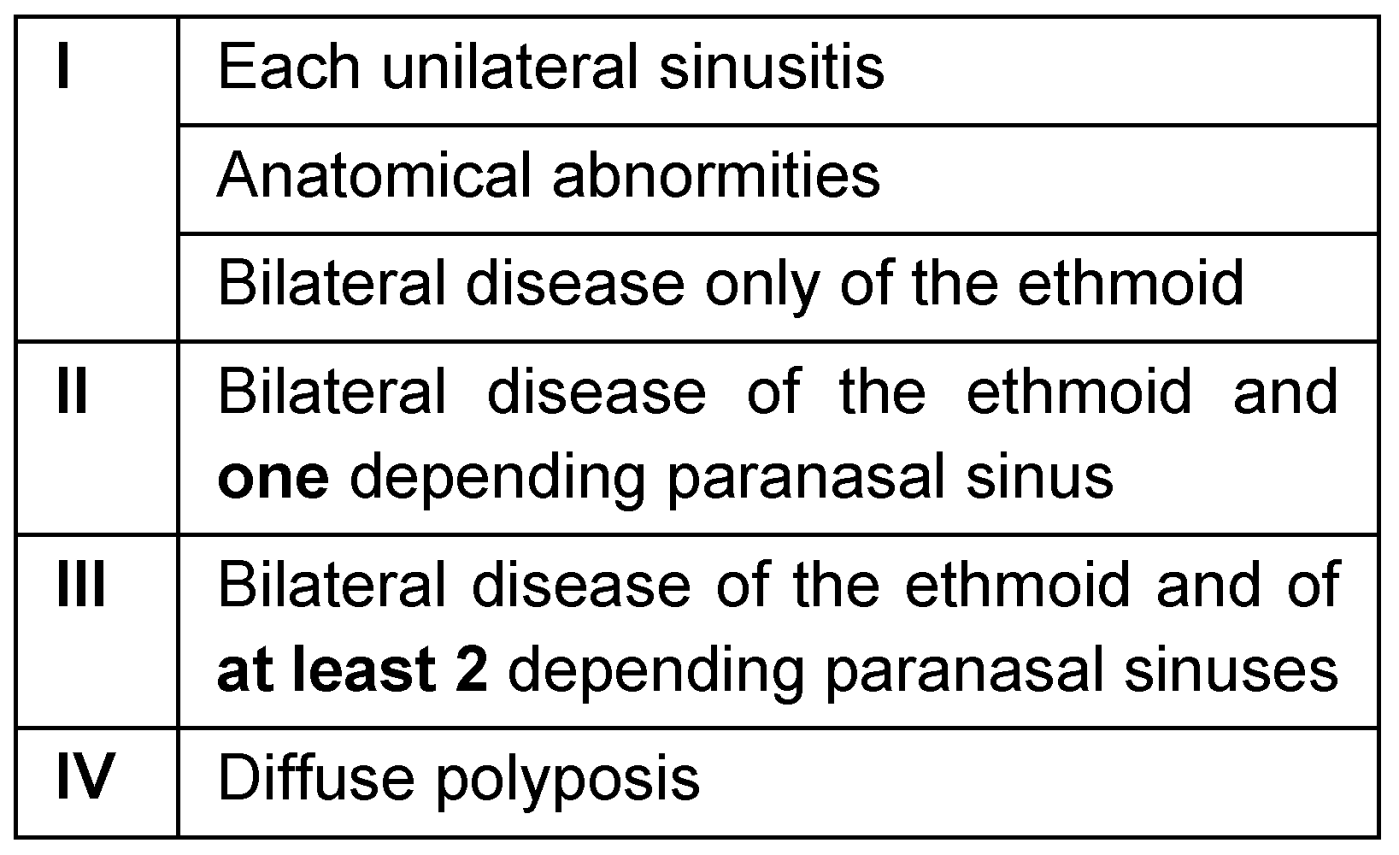
Staging classification of chronic rhinosinusitis according to Kennedy (based on CT scans)

**Table 3:**
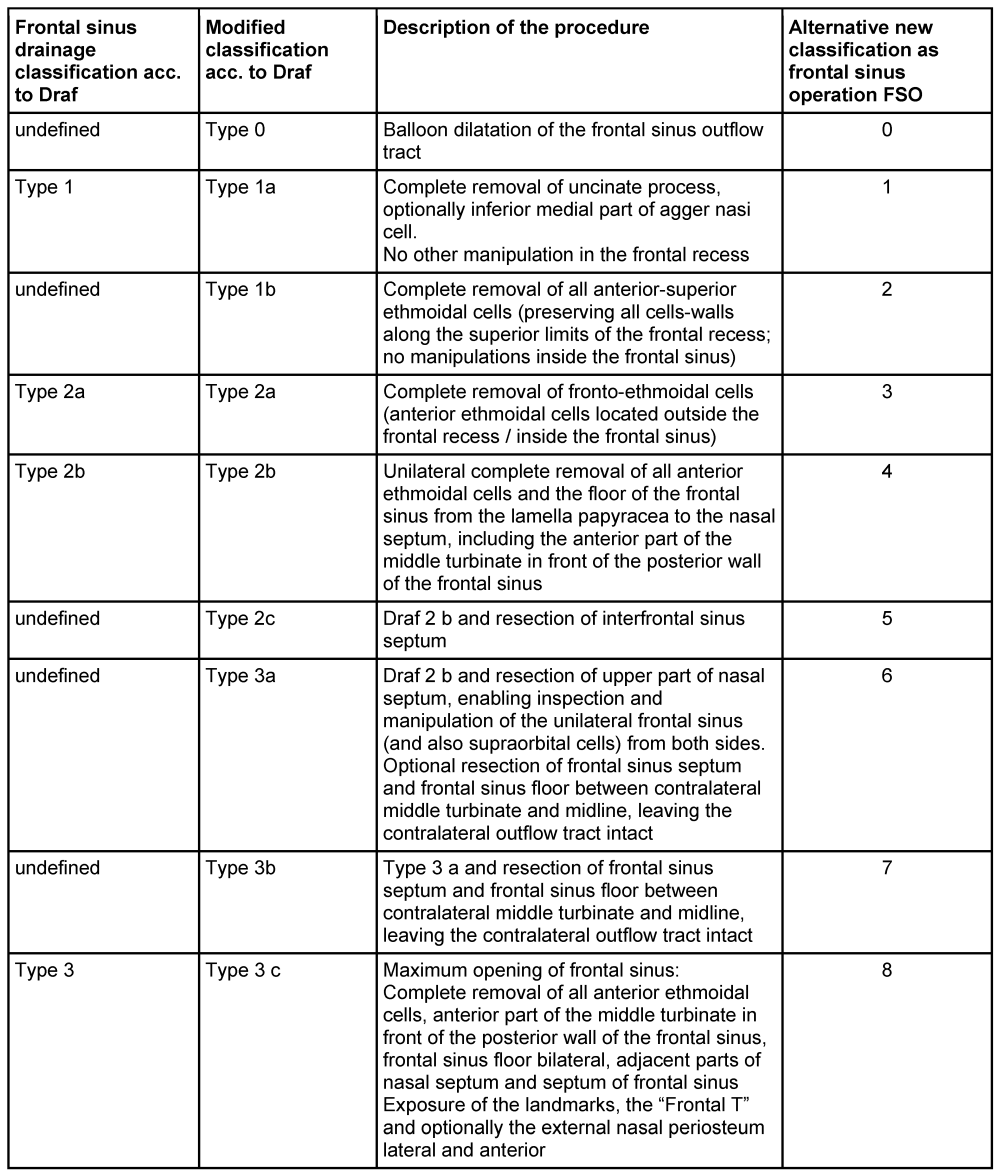
Classification of frontal sinus operations (FSO, modified classification according to Draf)

**Table 4:**
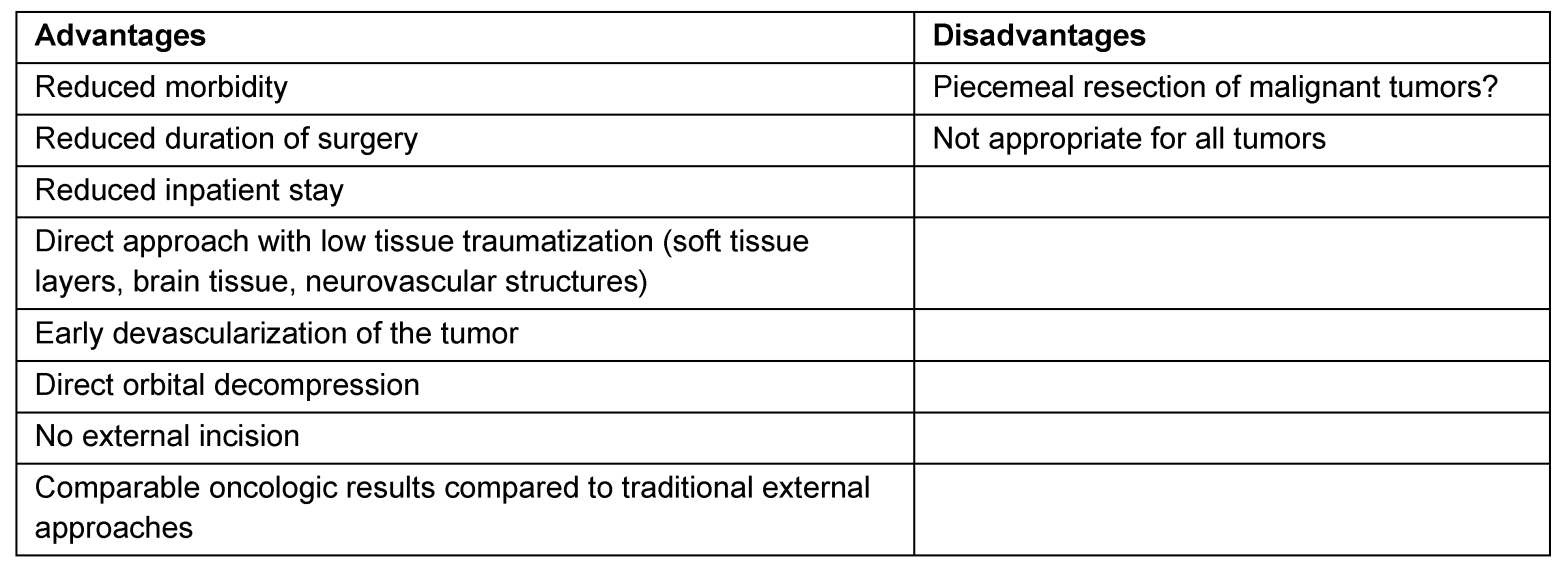
Advantages and disadvantages of endonasal endoscopic procedures for treatment of sinonasal tumors [9]

**Table 5:**
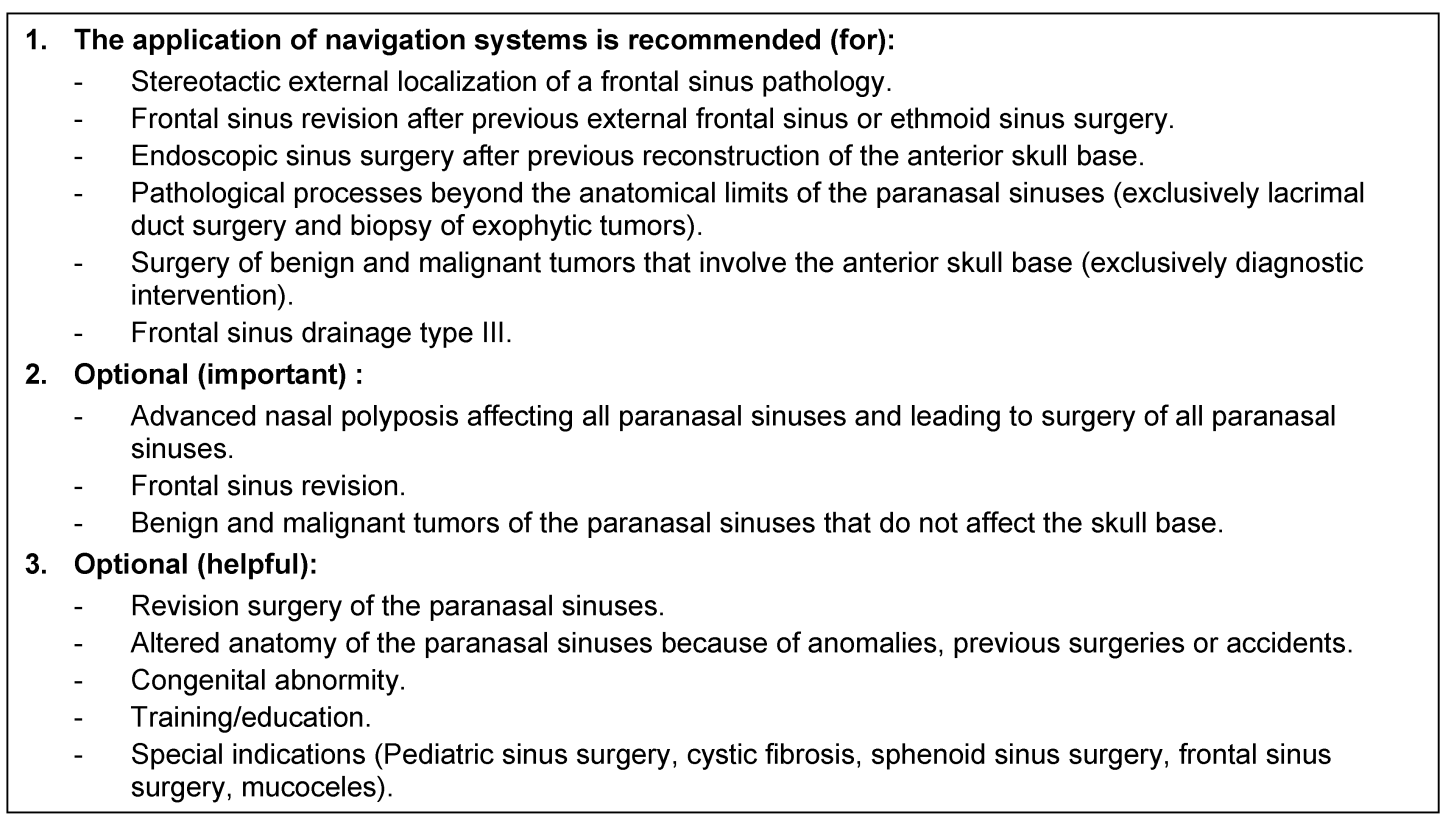
Recommendations for the use of navigation systems in the context of endonasal endoscopic sinus surgery according to the current literature [417]

**Table 6:**
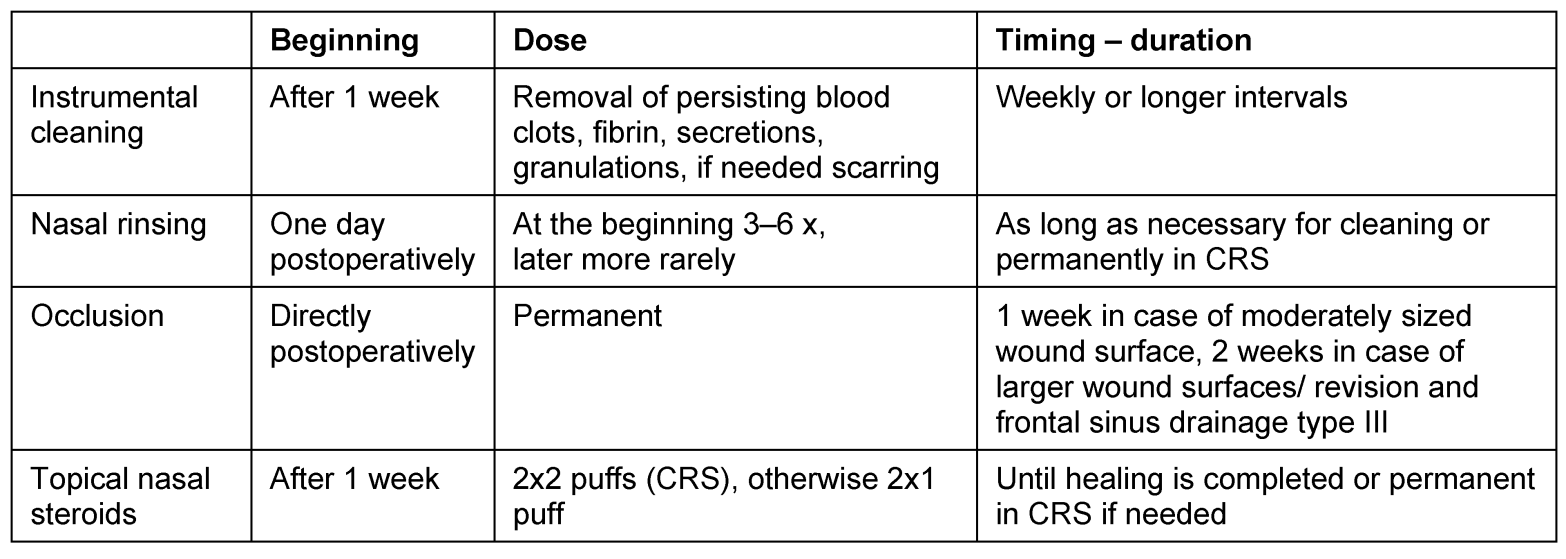
Recommendations on the basic standard postoperative care after endonasal endoscopic sinus surgery
